# Structures and Biologic Activity of Chitonoidosides I, J, K, K_1_ and L-Triterpene Di-, Tri- and Tetrasulfated Hexaosides from the Sea Cucumber *Psolus chitonoides*

**DOI:** 10.3390/md20060369

**Published:** 2022-05-30

**Authors:** Alexandra S. Silchenko, Sergey A. Avilov, Pelageya V. Andrijaschenko, Roman S. Popov, Ekaterina A. Chingizova, Pavel S. Dmitrenok, Anatoly I. Kalinovsky, Anton B. Rasin, Vladimir I. Kalinin

**Affiliations:** G.B. Elyakov Pacific Institute of Bioorganic Chemistry, Far Eastern Branch of the Russian Academy of Sciences, Pr. 100-letya Vladivostoka 159, 690022 Vladivostok, Russia; avilov_sa@piboc.dvo.ru (S.A.A.); andrijashchenko_pv@piboc.dvo.ru (P.V.A.); popov_rs@piboc.dvo.ru (R.S.P.); chingizova_ea@piboc.dvo.ru (E.A.C.); paveldmt@piboc.dvo.ru (P.S.D.); kaaniv@piboc.dvo.ru (A.I.K.); rasin_ab@piboc.dvo.ru (A.B.R.)

**Keywords:** *Psolus chitonoides*, triterpene glycosides, chitonoidosides, sea cucumber, cytotoxic activity

## Abstract

Five new triterpene di-, tri- and tetrasulfated hexaosides (chitonoidosides I (**1**), J (**2**), K (**3**), K_1_ (**4**) and L (**5**)) were isolated from the Far-Eastern sea cucumber *Psolus chitonoides*, collected near Bering Island (Commander Islands) from a depth of 100–150 m. The structural variability of the glycosides concerned both the aglycones (with 7(8)- or 9(11)-double bonds) and carbohydrate chains differing from each other by the third sugar residue (Xyl or sulfated by C-6 Glc) and/or by the fourth—terminal in the bottom semi-chain—residue (Glc or sulfated by C-6 MeGlc) as well as by the positions of a sulfate group at C-4 or C-6 in the sixth—terminal in the upper semi-chain—residue (MeGlc). Hemolytic activities of these compounds **1**–**5** against human erythrocytes as well as cytotoxicity against human cancer cell lines, HeLa, DLD-1 and HL-60, were studied. The hexaosides, chitonoidosides K (**3**) and L (**5**) with four sulfate groups, were the most active against tumor cells in all the tests. Noticeably, the sulfate group at C-4 of MeGlc6 did not decrease the membranolytic effect of **5** as compared with **3**, having the sulfate group at C-6 of MeGlc6. Erythrocytes were, as usual, more sensitive to the action of the studied glycosides than cancer cells, although the sensitivity of leukemia promyeloblast HL-60 cells was higher than that of other tumor cells. The glycosides **1** and **2** demonstrated some weaker action in relation to DLD-1 cells than against other tumor cell lines. Chitonoidoside K_1_ (**4**) with a hydroxyl at C 25 of the aglycone was not active in all the tests. The metabolic network formed by the carbohydrate chains of all the glycosides isolated from *P. chitonoides* as well as the aglycones biosynthetic transformations during their biosynthesis are discussed and illustrated with schemes.

## 1. Introduction

The history of investigation of chemical structures [1,2,3,4,5], biological activities [6,7,8,9,10,11], evolution [2,12,13,14], taxonomic specificity of triterpene glycosides from the sea cucumbers [2,3,15,16,17], their biosynthesis [18,19], ecological role and functions [8,20] as well as structure–activity relationships [21,22] is rather long. However, the recent researches demonstrate the further perspectives in this field due to the finding of new unusual structures, the deepening of understanding of biosynthetic process leading to the glycosides and the appearance of the other aspects concerning their origin and properties, including the modelling in silico mechanisms of interaction of the glycosides with biological targets [22].

The Far Eastern sea cucumber *Psolus chitonoides* contains a complicated multicomponent mixture of triterpene glycosides, mainly highly polar, the part of which was recently separated and a series of new glycosides, namely chitonoidosides A–H, have been isolated [23,24]. As a continuation of our studies on the glycosides from *P. chitonoides*, the structure elucidation of new chitonoidosides I (**1**), J (**2**), K (**3**), K_1_ (**4**) and L (**5**) is reported. The chemical structures of **1**–**5** were established by the analyses of the ^1^H, ^13^C NMR, 1D TOCSY and 2D NMR (^1^H, ^1^H-COSY, HMBC, HSQC and ROESY) spectra as well as HR-ESI mass spectra. All the original spectra are presented in Figures S1–S40 in the Supplementary data. The hemolytic activity against human erythrocytes, cytotoxic activities against human adenocarcinoma HeLa, colorectal adenocarcinoma DLD-1 and leukemia promyeloblast HL-60 cells were examined.

## 2. Results and Discussion

### 2.1. Structural Elucidation of the Glycosides

**Figure 1:**
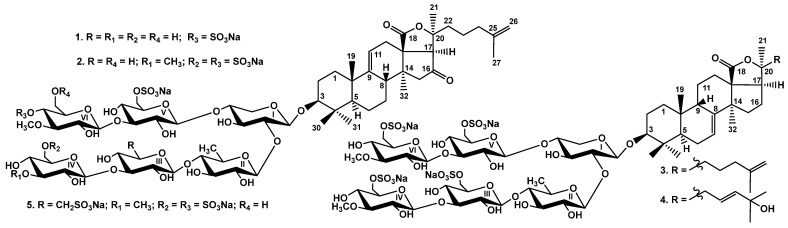
Chemical structures of glycosides isolated from *Psolus chitonoides*: **1**—chitonoidoside I; **2**—chitonoidoside J; **3**—chitonoidoside K; **4**—chitonoidoside K_1_; and **5**—chitonoidoside L.

The crude glycosidic fraction of the sea cucumber *Psolus chitonoides* was obtained as result of the hydrophobic chromatography on a Polychrom-1 column (powdered Teflon, Biolar, Latvia) of the concentrated ethanolic extract. Its subsequent separation by chromatography on Si gel columns with the stepped gradient of eluents CHCl3/EtOH/H2O (100:75:10), (100:100:17), (100:125:25) and (100:150:50) gave the fractions I–VII. The individual compounds **1**–**5** (Figure 1) were isolated by HPLC of the fractions V–VII on a silica-based column Supelcosil LC-Si (4.6 *×* 150 mm), reversed-phase semipreparative columns Supelco Ascentis RP-Amide (10 *×* 250 mm) and Phenomenex Synergi Fusion RP (10 × 250 mm).

The configurations of monosaccharide residues in the glycosides **1**–**5** were assigned as *D* based on the biogenetic analogies with all other known sea cucumber triterpene glycosides.

The aglycones of chitonoidosides I (**1**), J (**2**) and L (**5**) (Tables S1, S2 and S4 and Figures S1–S6, S9–S14 and S33–S38) were identical to each other and to holotoxinogenin, a common aglycone for many sea cucumber glycosides [3,4,5,25]. The same aglycone was also found in the other six glycosides previously isolated from *P. chitonoides* [23,24].

The molecular formula of chitonoidoside I (**1**) was determined to be C_65_H_100_O_37_S_2_Na_2_ from the [M_2Na_–2Na]^2−^ peak at *m*/*z* 768.2694 (calc. 768.2698) in the (*−*)HR-ESI-MS (Figure S8). The ^1^H and ^13^C NMR spectra of the carbohydrate chain of chitonoidoside I (**1**) (Table 1, Figures S1–S7) demonstrated six characteristic doublets of anomeric protons at δ_H_ 4.66–5.20 (*J* = 6.3–8.2 Hz) and six signals of anomeric carbons at δ_C_ 102.4–104.7.

Analysis of the ^1^H,^1^H-COSY, 1D TOCSY, HSQC and ROESY spectra of **1** indicated the presence of two xylose residues (Xyl1 and Xyl3), one quinovose (Qui2), two glucose (Glc4 and Glc5) and 3-O-methylglucose (MeGlc6) residues. The monosaccharides were connected to each other and to the aglycone by *β*-glycosidic bonds located at typical positions for this class of metabolites, which was confirmed by the correlations in the ROESY and HMBC spectra: H-1 Xyl1/H-3 (C-3) of the aglycone, H-1 Qui2/H-2 (C-2) Xyl1, H-1 Xyl3/H-4 (C-4) Qui2, H-1 Glc4/H-3 (C-3) Xyl3, H-1 Glc5/H-4 (C-4) Xyl1 and H-1 MeGlc6/H-3 (C-3) Glc5 (Table 1, Figures S5 and S6).

The terminal residues in hexasaccharide chains of the holothuroid’s glycosides usually contain 3-*O*-methyl groups as the “signals of termination” that stop the elongation of the sugar chain in the process of biosynthesis [26]. The presence of the signals of 3-*O*-methyl group in the ^1^H and ^13^C NMR spectra of chitonoidoside I (**1**) at δ_H_ 3.92 (s) and δ_C_ 60.7, correspondingly, indicated that only one terminal monosaccharide unit was methylated while another one did not have this “stop-signal”.

The analysis of the ^1^H,^1^H-COSY, HSQC and 1D TOCSY spectra of **1** showed that the fourth monosaccharide unit attached to C-3 Xyl3 was glucose (Glc4) (Table 1) without the 3-*O*-methyl group; therefore, the signal of C-3 Glc4 was shielded to δ_C_ 77.2. While the sixth unit was 3-*O*-methylated MeGlc with deshielded signal of C-3 MeGlc6 at δ_C_ 85.2 due to the attachment of OMe-group. It was supposed that the carbohydrate chain of **1** contained two sulfate groups with one of them attached to C-6 Glc5 based on the signal at δ_C_ 67.3. This sulfate position is common for the majority of glycosides from *P. chitonoides*, which have upper semi-chain attached to C-4 Xyl1 [23,24].

The position of the second sulfate group was determined as C-4 in 3-O-methylated Glc6 due to the deshielding of this carbon signal to δ_C_ 76.1 (*α*-shifting effect of sulfate group) and the shielding of the signals C-3 MeGlc6 and C-5 MeGlc6 to δ_C_ 85.2 and 76.3 (*β*-shifting effect of sulfate group), respectively, when compared with corresponding signals of non-sulfated MeGlc6 residue in chitonoidoside G [24] (δ_C_ 70.3 (C-4 MeGlc6), δ_C_ 86.8 (C-3 MeGlc5), δ_C_ 77.5 (C-5 MeGlc6)).

The ^13^C NMR signals of the sugar units composing the upper semi-chain of chitonoidoside I (**1**) were coincident with the corresponding signals in the ^13^C NMR spectrum of chitonoidoside H [24] corroborating the sulfation of C-6 Glc5 and C-4 MeGlc6. The signal of C-6 Glc4 at δ_C_ 62.0 was characteristic of non-sulfated hydroxymethyl group of glucopyranose unit. Generally, these data indicate the presence of a new hexasaccharide chain with terminal glucose unit in the bottom semi-chain and two sulfate groups in the upper one in chitonoidoside I (**1**).

The (*−*)ESI-MS/MS of **1** (Figure S8) demonstrated the fragmentation of [M_2Na_–2Na]^2−^ ion at *m*/*z* 768.3 with the ion-peaks observed at *m*/*z* 687.2 [M_2Na_–2Na−Glc]^2−^, 621.2 [M_2Na_–2Na−Glc–Xyl]^2−^, 548.2 [M_2Na_–2Na−Glc−Xyl–Qui]^2−^ and 322.0 [M_2Na_–2Na−Glc−Xyl−Qui–Agl]^2−^ corroborating the sequence of monosaccharides in the bottom semi-chain and the aglycone structure of **1**.

These data indicate that chitonoidoside I (**1**) is 3*β*-*O*-{*β*-d-glucopyranosyl-(1→3)-*β*-d-xylopyranosyl-(1→4)-*β*-d-quinovopyranosyl-(1→2)-[4-*O*-sodium sulfate-3-*O*-methyl-*β*-d-glucopyranosyl-(1→3)-6-*O*-sodium sulfate-*β*-d-glucopyranosyl-(1→4)]-*β*-d-xylopyranosyl}-16-oxo-holosta-9(11),25(26)-diene.

The molecular formula of chitonoidoside J (**2**) was determined to be C_66_H_101_O_40_S_3_Na_3_ from the [M_3Na_–Na]^−^ ion peak at *m*/*z* 1675.4855 (calc. 1675.4832), [M_3Na_–2Na]^2−^ ion peak at *m*/*z* 826.2495 (calc. 826.2470) and [M_3Na_–3Na]^3−^ ion peak at *m*/*z* 543.1700 (calc. 543.1683) in the (−)HR-ESI-MS (Figure S16). The ^1^H NMR spectrum of the carbohydrate part of chitonoidoside J (**2**) showed six characteristic doublets at δ_H_ 4.66–5.19 (*J* = 6.7–7.6 Hz), correlated by the HSQC spectrum with corresponding anomeric carbon signals at δ_C_ 102.7–104.5.

These signals indicated the presence of hexasaccharide chain with *β*-configurations of glycosidic bonds (Table 2, Figures S9–S15). The comparison of the ^13^C NMR spectra of carbohydrate chains of **1** and **2** showed the closeness of the signals of five monosaccharide residues with exception of the signals of terminal (fourth) residue. 3-*O*-methylglucose sulfated by C-6 was established to occupy the fourth position in the carbohydrate chain of **2** based on the analysis of the ^1^H-^1^H COSY, 1D TOCSY and HSQC spectra.

The signal of C-3 MeGlc4 was observed at δ_C_ 86.4 due to the *O*-methylation and the signal of C-6 MeGlc4 was deshielded to δ_C_ 66.6 due to *α*-shifting effect of sulfate group. The signals of two 3-*O*-methyl groups, observed at δ_H_ 3.76 (s) and 3.97 (s), were correlated by the HMBC spectrum with C-3 MeGlc4 and C-3 MeGlc6, correspondingly, demonstrating the both terminal sugar units were *O*-methylated.

The presence of three sulfate groups were deduced from the availability of three-charged ion-peak in the HR-ESI-MS as well as from the shifting effects observed in ^13^C NMR spectrum. Thus, the first sulfate group was attached to C-6 MeGlc4 (δ_C_ 66.6), the second one to C-6 Glc5 (δ_C_ 67.0) and the third to C-4 MeGlc6 (δ_C_ 75.5). The positions of glycosidic linkages in **2** were established by the ROESY and HMBC spectra in the same manner as for **1** (Table 2, Figures S13 and S14).

The (*−*)ESI-MS/MS of **2** (Figure S16) demonstrated the fragmentation of [M_3Na_–Na]^−^ ion at *m*/*z* 1675.5 resulting in the ion-peaks appearance at *m*/*z* 1277.5 [M_3Na_–Na−MeGlcOSO_3_Na–NaHSO_4_]^−^, 987.4 [M_3Na_–Na−2MeGlcOSO_3_Na−Xyl]^−^ and 841.4 [M_3Na_–Na−2MeGlcOSO_3_Na−Xyl–Qui]^−^. The fragmentation of [M_3Na_–2Na]^2−^ ion at *m*/*z* 826.2 led to the ion peaks at *m*/*z* 811.2 [M_3Na_–2Na−OMe]^2−^, 775.3 [M_3Na_–2Na−NaSO_3_]^2−^, 760.3 [M_3Na_–2Na−Ome–NaSO_3_]^2−^ and 687.2 [M_3Na_–2Na−MeGlcOSO_3_Na]^2−^. Therefore, compound **2** contains a new carbohydrate chain for the sea cucumber glycosides. This is the first trisulfated glycoside found in *P. chitonoides*. This glycoside, together with kuriloside H, isolated recently from *Thyonidium kurilensis* [27], forms a group of highly polar hexaosides with three sulfate groups.

All these data indicate that chitonoidoside J (**2**) is 3*β*-*O*-{6-*O*-sodium sulfate-3-*O*-methyl-*β*-d-glucopyranosyl-(1→3)-*β*-d-xylopyranosyl-(1→4)-*β*-d-quinovopyranosyl-(1→2)-[4-*O*-sodium sulfate-3-*O*-methyl-*β*-d-glucopyranosyl-(1→3)-6-*O*-sodium sulfate-*β*-d-glucopyranosyl-(1→4)]-*β*-d-xylopyranosyl}-16-oxo-holosta-9(11),25(26)-diene.

The ^1^H and ^13^C NMR spectra of the carbohydrate chain of chitonoidosides K (**3**) and K_1_ (**4**) (Table 3 and Table S3, Figures S17–S23, S25–S31) were coincident to each other indicating the identity of these parts for the molecules of **3** and **4**. The HSQC spectrum of **3** demonstrated six signals of anomeric protons at δ_H_ 4.57–5.11 (d, *J* = 7.3–8.6 Hz) and corresponding signals of anomeric carbons at δ_C_ 103.2–104.6, indicating the presence of a hexasaccharide moiety with *β*-glycosidic bonds.

The monosaccharide composition of **3** was determined on the base of analysis of the ^1^H,^1^H-COSY, HSQC, 1D TOCSY and ROESY spectra as one xylose (Xyl1), one quinovose (Qui2), two glucoses (Glc3 and Glc5) and two 3-*O*-methylglucoses (MeGlc4 and MeGlc6). Hence, it was different from the other hexaosides of *P. chitonoides* due to the replacement of xylose residue (Xyl3) in the bottom semi-chain for glucose (Glc3) one. The isolated spin system of the third monosaccharide attached to C-4 Qui2 (the ROE-correlation between the signals at δ_H_ 4.64 (H-1 Glc3) and 3.25 (H-4 Qui2)) was deduced by the ^1^H,^1^H-COSY and 1D TOCSY spectra.

The corresponding carbon signals found by the HSQC spectrum were assigned to glucopyranose residue sulfated at C-6 Glc3 (δ_C_ 67.5). Noticeably, all the signals of hydroxylated methylene groups were deshielded due to *α*-shifting effects of sulfate groups and observed at δ_C_ 67.0 (the doubled intensity of the signal corresponding to C-6 MeGlc4 and C-6 MeGlc6), 67.5 (C-6 Glc3) and 67.7 (C-6 Glc5) in the ^13^C NMR spectrum of the carbohydrate chain of **3**.

The signals of C-5 Glc3, C-5 MeGlc4, C-5 Glc5 and C-5 MeGlc6 were shielded to δ_C_ 74.3, 75.5, 74.4 and 75.5, correspondingly, due to the *β*-shifting effects of sulfate groups. These data indicated the presence of four sulfate groups at C-6 of *O*-methylated and non-methylated glycopyranose residues in the carbohydrate chain of chitonoidosides K (**3**) and K_1_ (**4**). The HR-ESI-MS data also confirmed that **3** and **4** are tetrasulfated compounds by the presence of four-charged ion peaks.

The molecular formula of chitonoidoside K (**3**) was determined to be C_67_H_104_O_43_S_4_Na_4_ from the [M_4Na_–2Na]^2−^ ion peak at *m*/*z* 885.2353 (calc. 885.2320), [M_4Na_–3Na]^3−^ ion peak at *m*/*z* 582.4939 (calc. 582.4916) and [M_4Na_–4Na]^4−^ ion peak at *m*/*z* 431.1223 (calc. 431.1214) in the (*−*)HR-ESI-MS (Figure S24). The ^13^C NMR spectrum of the aglycone part of **3** differed from those of the other chitonoidosides isolated thus far by the presence of the signals of 7(8)-double bond at δ_C_ 119.8 (C-7) and 146.7 (C-8) instead of the signals characteristic for 9(11)-double bond (Table 4, Figures S17–S22).

The COSY correlation H-6/H-7 (1.88/5.64) and long-range correlation H-9/H-7 (3.32/5.64) indicate the existence of 7(8)-double bond in the nucleus **3**. The Δ7(8) position was also confirmed by the HMBC correlation between the signals of methyl group H_3_-32 (δ_H_ 1.08 (s)) and C-8 (δ_C_ 146.7) as well as by the ROE-correlations between H-7 (δ_H_ 5.64) and H-15 (δ_H_ 1.73), H-7 and H-32 (δ_H_ 1.08 (s)). Additionally, the ^13^C and ^1^H NMR spectra of the aglycone part of chitonoidoside K (**3**) demonstrated the signals of quaternary oxygen-bonded carbons at δ_C_ 180.9 (C-18) and 84.7 (C-20) assigning to 18(20)-lactone as well as the signals characteristic of the terminal 25(26)-double bond at δ_C_ 145.3 (C-25) and 110.7 (C-26) and at δ_H_ 4.70 and 4.66 (H_2_-26, both brs) (Table 4, Figures S17–S22).

The protons H_2_-15/H_2_-16/H-17 formed an isolated spin system in the COSY spectrum, and the signal of methylene group CH_2_-16 was observed at δ_C_ 24.4. Both signals of C-15 and C-17 were shielded to δ_C_ 34.1 and δ_C_ 53.0 as compared with the corresponding signals in the aglycone of **1** having 16-oxo-group. These data indicate the absence of the functional group at C-16 in the holostane nucleus of **3**. The same aglycone was previously found in four other glycosides isolated from the sea cucumbers *Cucumaria japonica* [28,29], *Colochirus robustus* [30] and *Psolus fabricii* [31].

The (*−*)ESI-MS/MS of **3** (Figure S24) demonstrated the fragmentation of [M_4Na_–2Na]^2−^ ion at *m*/*z* 885.2 resulting in the ion-peaks appearance at *m*/*z* 665.2 [M_4Na_–2Na−Agl–H]^2−^, 622.2 [M_4Na_–2Na−MeGlcOSO_3_Na−GlcOSO_3_Na]^2−^ and 550.2 [M_4Na_–2Na−MeGlcOSO_3_Na−GlcOSO_3_Na–Qui]^2−^. The fragmentation of [M_4Na_–3Na]^3−^ ion at *m*/*z* 582.5 showed the presence of the ion peak at *m*/*z* 489.8 [M_4Na_–3Na−MeGlcOSO_3_Na]^3−^, 385.1 [M_4Na_–3Na−2C_7_H_12_O_9_SNa (MeGlcOSO_3_Na)]^3−^, thus, confirming the structure of **3**.

These data indicate that chitonoidoside K (**3**) is 3*β*-*O*-{6-*O*-sodium sulfate-3-*O*-methyl-*β*-d-glucopyranosyl-(1→3)-6-*O*-sodium sulfate-*β*-d-glucopyranosyl-(1→4)-*β*-d-quinovopyranosyl-(1→2)-[6-*O*-sodium sulfate-3-*O*-methyl-*β*-d-glucopyranosyl-(1→3)-6-*O*-sodium sulfate-*β*-d-glucopyranosyl-(1→4)]-*β*-d-xylopyranosyl}-holosta-7(8),25(26)-diene.

The molecular formula of chitonoidoside K_1_ (**4**) was determined to be C_67_H_104_O_44_S_4_Na_4_ from the [M_4Na_–2Na]^2−^ ion peak at *m*/*z* 893.2272 (calc. 893.2295), [M_4Na_–3Na]^3−^ ion peak at *m*/*z* 587.8228 (calc. 587.8232) and [M_4Na_–4Na]^4−^ ion peak at *m*/*z* 435.1200 (calc. 435.1201) in the (*−*)HR-ESI-MS (Figure S32).

The ^13^C NMR spectrum of the aglycone part of **4** demonstrated the signals of 7(8)-double bond at δ_C_ 120.5 (C-7) and 147.4 (C-8) similarly to that of chitonoidoside K (**3**) (Table 5, Figures S25–S30). The polycyclic system of chitonoidoside K_1_ (**4**) was the same as in **3**, which was deduced from the analysis of 1D and 2D NMR spectra but the signals corresponding to the side chains of these glycosides were different. Analysis of the ^1^H,^1^H-COSY spectrum of **4** led to the detection of isolated spin system formed by the protons H_2_-22, H-23, H-24. The values of their δ_H_ and coupling constants (δ_H_ 2.43 (brt, *J* = 7.0 Hz, H-22), 5.78 (dt, *J* = 7.1; 15.0 Hz, H-23) and 5.91 (d, *J* = 15.0 Hz, H-24)) showed the presence of the 23*E*,24-double bond.

The coincidence of the signals of methyl groups C-26 and C-27 to each other (δ_C_ 30.6, C-26(27) and δ_H_ 1.48 (s), H-26(27)) indicated the presence of an OH-group at C-25 that was confirmed by the HMBC correlation H-26(27)/C-25 (1.48/70.7). The deshielding of C-25 signal to δ_C_ 70.7 was characteristic for the hydroxylated carbon (Table 5, Figures S25–S30). The same aglycone was previously found only in cucumarioside A_2_-3 from *Cucumaria frondosa* [32].

The (*−*)ESI-MS/MS of **4** (Figure S32) demonstrated the fragmentation of [M_4Na_–2Na]^2−^ ion at *m*/*z* 893.2 resulting in the ion-peaks appearance at *m*/*z* 754.2 [M_4Na_–2Na−MeGlcOSO_3_Na]^2−^, 622.2 [M_4Na_–2Na−MeGlcOSO_3_Na−GlcOSO_3_Na]^2−^. The fragmentation of [M_4Na_–3Na]^3−^ ion at *m*/*z* 587.8 led to the presence of the ion peak at *m*/*z* 495.2 [M_4Na_–3Na−MeGlcOSO_3_Na]^3−^.

These data indicate that chitonoidoside K_1_ (**4**) is 3*β*-*O*-{6-*O*-sodium sulfate-3-*O*-methyl-*β*-d-glucopyranosyl-(1→3)-6-*O*-sodium sulfate-*β*-d-glucopyranosyl-(1→4)-*β*-d-quinovopyranosyl-(1→2)-[6-*O*-sodium sulfate-3-*O*-methyl-*β*-d-glucopyranosyl-(1→3)-6-*O*-sodium sulfate-*β*-d-glucopyranosyl-(1→4)]-*β*-d-xylopyranosyl}-25-hydroxyholosta-7(8),23*E*(24)-diene.

The molecular formula of chitonoidoside L (**5**) was determined to be C_67_H_102_O_44_S_4_Na_4_ from the [M_4Na_–2Na]^2−^ ion peak at *m*/*z* 892.2234 (calc. 892.2217) and [M_4Na_–3Na]^3−^ ion peak at *m*/*z* 587.1535 (calc. 587.1510) and [M_4Na_–4Na]^4−^ ion peak at *m*/*z* 434.6182 (calc. 434.6160) in the (−)HR-ESI-MS (Figure S40). The ^1^H NMR spectrum of the carbohydrate part of chitonoidoside L (**5**) demonstrated six characteristic doublets at δ_H_ 4.62–5.17 (*J* = 7.5–8.5 Hz), which were correlated by the HSQC spectrum with corresponding anomeric carbon signals at δ_C_ 103.1–104.6.

These signals indicated the presence of a hexasaccharide chain with *β*-configurations of glycosidic bonds (Table 6, Figures S33–S39). The comparison of the ^13^C NMR spectra of carbohydrate chains of chitonoidosides K (**3**) and L (**5**) showed the closeness of the signals of five monosaccharide residues with exception of the signals of terminal (sixth) residue. Analysis of the ^1^H-^1^H COSY, 1D TOCSY and HSQC spectra of this monosaccharide residue in **5** allowed us to deduce all the signals of protons and carbons, which indicated that it was 3-*O*-methylated Glc6 sulfated by C-4.

The signal of C-4 MeGlc6 was deshielded to δ_C_ 76.1 (as compared to the same signal in **3** observed at δ_C_ 69.8) due to the *α*-shifting effect of sulfate group. The signal of C-6 MeGlc6 was observed at δ_C_ 61.7 indicating the absence of a sulfate group at this position in the sugar chain of **5**. Thus, chitonoidoside L (**5**) as well as chitonoidosides K (**3**) and K_1_ (**4**) are tetrasulfated hexaosides—more polar glycosides than tetrasulfated pentaosides, psolusosides P and Q, containing two sulfate groups bonded to one (glucose) residue, which were found recently in *Psolus fabricii* [33]. Moreover, tetrasulfated hexasaccharide chains of **3**–**5** containing sulfate groups at monosaccharide residues in different positions are novel, and these compounds are the most polar glycosides of the sea cucumbers found so far.

The (*−*)ESI-MS/MS of chitonoidoside L (**5**) (Figure S40) demonstrated the fragmentation of [M_4Na_–2Na]^2−^ ion at *m*/*z* 892.2 leading to the presence of the ion peaks at *m*/*z* 621.2 [M_4Na_–2Na−MeGlcOSO_3_Na]^2−^ and 548.2 [M_4Na_–2Na−MeGlcOSO_3_Na–GlcOSO_3_Na]^2−^ with fragmentation of [M_4Na_–3Na]^3^ ion at *m*/*z* 587.1 that resulted in the appearance of the ion peak at *m*/*z* 494.8 [M_4Na_–3Na−MeGlcOSO_3_Na]^3−^.

These data indicate that chitonoidoside L (**5**) is 3*β*-*O*-{6-*O*-sodium sulfate-3-*O*-methyl-*β*-d-glucopyranosyl-(1→3)-6-*O*-sodium sulfate-*β*-d-glucopyranosyl-(1→4)-*β*-d-quinovopyranosyl-(1→2)-[4-*O*-sodium sulfate-3-*O*-methyl-*β*-d-glucopyranosyl-(1→3)-6-*O*-sodium sulfate-*β*-d-glucopyranosyl-(1→4)]-*β*-d-xylopyranosyl}-16-oxo-holosta-9(11),25(26)-diene.

### 2.2. Bioactivity of the Glycosides

The cytotoxic activity of chitonoidosides I–L (**1**–**5**) against human cell lines, including erythrocytes and cancer cell lines, HeLa, DLD-1 and HL-60, was studied. The previously tested chitonoidoside A [23] as well as cisplatin were used as the positive controls (Table 7). The tetrasulfated hexaosides, chitonoidosides K (**3**) and L (**5**) were the most active in all the tests. Noticeably, the sulfate group at C-4 MeGlc6 did not decrease the membranolytic effect of **5** as compared with **3**, having the sulfate group at C-6 MeGlc6.

It is known that diverse cell lines exhibit a differential sensitivity to the cytotoxic action of sea cucumber glycosides depending on both their chemical structures and the composition of the cellular membranes [10]. In the current experiments, erythrocytes were, as usual, more sensitive to the action of the glycosides, than cancer cells, but leukemia promyeloblast HL-60 cells displayed only slightly lesser sensitivity, than erythrocytes.

The glycosides **1** and **2** demonstrated some negative selectivity in relation to DLD-1 cells, being less cytotoxic when compared with their effect on the other cell lines. Chitonoidosides I (**1**) and J (**2**), di- and trisulfated hexaosides, correspondingly, with a xylose residue in the bottom semi-chain demonstrated lower cytotoxicity in comparison with chitonoidosides K (**3**) and L (**5**) having four sulfate groups and glucose residue in the bottom semi-chain. Chitonoidoside K_1_ (**4**) was expectedly inactive in all experiments due to the presence of a hydroxyl group in the aglycone side chain [10,22].

Thus, almost all the glycosides isolated from *P. chitonoides* demonstrated high hemolytic and high or moderate cytotoxic activities against cancer cells. In contrast with typical proposals, the increasing of the number of sulfate groups in the carbohydrate chains does not decrease the activity of the glycosides. Moreover, tetrasulfated hexaosides are among the most active compounds.

### 2.3. The Biosynthetic Peculiarities of the Glycosides from P. chitonoides

**Figure 2:**
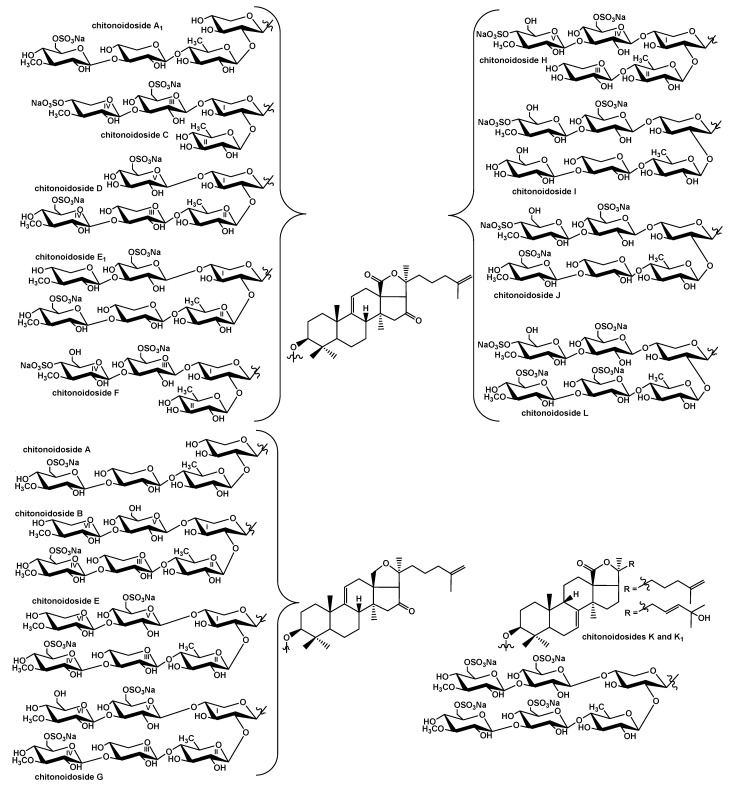
Combinatorial library for the glycosides of *P. chitonoides*.

The glycosides of the sea cucumber *P. chitonoides* are characterized by different structural diversity of their aglycone and carbohydrate parts (Figure 2). Thus, four diverse aglycones were found among fifteen isolated chitonoidosides A–L [23,24], including the new type, lacking 18(20)-lactone but containing an 18(20)-ether bond.

The structural variability of the carbohydrate chains was much more remarkable: twelve types of oligosaccharide chains were discovered [23,24], i.e., 12 groups of chitonoidosides named with the capital letters A–L, based on the sugar part structures. The major part of the groups of chitonoidoides is formed by the only compound with exception of the groups A and E. All the last isolated compounds (**1**–**5**) had hexasaccharide chains with two, three or four sulfate groups.

Noticeably, the glycosides of *P. chitonoides* are predominantly hexaosides differing from each other by the sugar residues in the bottom semi-chain (the third (Xyl3 or Glc3) and fourth (Glc4 or sulfated MeGlc4) units) and terminal residues in the upper semi-chain (MeXyl6 or MeGlc6) as well by the number (from one to four) and positions (at C-4 or C-6 of terminal MeGlc6) of sulfate groups. Such diversity is formed due to the mosaic type of biosynthesis of the sea cucumber triterpene glycosides, when the enzymatic reactions of glycosylation, 3-*O*-methylation and sulfation of the forming carbohydrate chains are shifted in time relative to each other [19,24,26].

**Figure 3:**
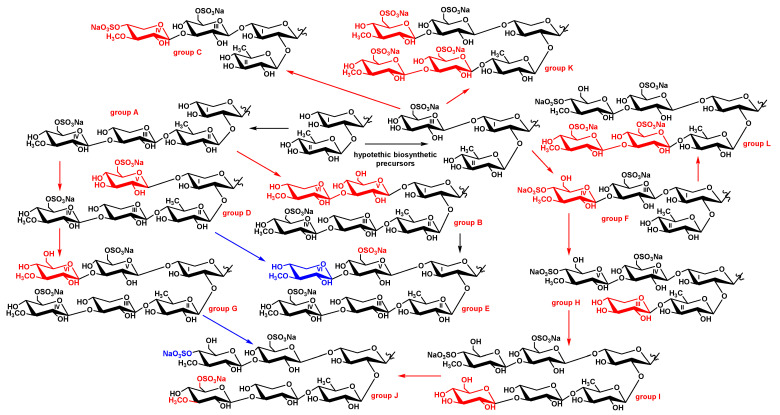
Biosynthetic network of carbohydrate chains of the glycosides isolated from *P. chitonoides*. Red indicates main steps of transformations and blue indicates the alternative path of biosynthetic modifications.

Considering the biosynthesis of sugar moieties occurs through sequential bonding of monosaccharides to the certain positions of biosynthesizing carbohydrate chains, chitonoidoside H [24] is a precursor of chitonoidoside I (**1**) (Figure 3). It is interesting that none of the hexaosides found earlier (chitonoidosides of the groups B [23], E or G [24]) could have been a biosynthetic precursor of the carbohydrate chain of chitonoidoside I (**1**) because of the differences in monosaccharide composition, determined by the presence of 3-*O*-methylxylose as the sixth unit in the first two groups or the sulfation and 3-*O*-methylation of the fourth (glucose) unit in chitonoidoside G.

Chitonoidoside J (**2**) can be formed from **1** through 3-*O*-methylation and sulfation by C-6 of its fourth (glucose) unit, wherein the sulfation of the sixth sugar precedes to the transformations of the fourth residue (red color in the Figure 3). Alternatively, the carbohydrate chain of **2** can be formed from the chain of chitonoidoside G [24] through the sulfation by C-4 of its MeGlc6 unit (blue color in the Figure 3).

The carbohydrate moiety of chitonoidoside L (**5**) could biosynthesized from the tetrasaccharide chain of chitonoidoside F through four steps: glycosylation with Glc3 and Glc4, followed by *O*-methylation and sulfation. None of the hexaosides previously found in *P. chitonoides* are suited to be the precursor of the sugar chain chitonoidoside L (**5**). Therefore, the hexasaccharide moieties of the glycosides of *P. chitonoides* are formed as result of an individual sequence of enzymatic reactions of glycosylation, methylation and sulfation leading to the substantial diversity of their structures (Figure 3).

Chitonoidosides of the group K (**3**, **4**) structurally stand out from the other glycosides of *P. chitonoides* both by the aglycones and sugar chains. The branchpoint leading to the carbohydrate moiety of this group is on the early stage of its formation when the precursor consists of three monosaccharides, including sulfated glucose attached to C-4 Xyl1. Then, three-stepped glycosylation of the bottom and upper semi-chains takes place leading to the hypothetic hexasaccharide precursor with the same sugar composition as chitonoidoside L (**5**).

However, chitonoidosides of the groups K and L differ by the character of sulfation. Chitonoidoside F is a biosynthetic precursor of chitonoidoside L (**5**), but it cannot be a precursor of chitonoidosides of the group K because of the difference in the positions of sulfate group in MeGlc6 residue. Significantly, compounds **3** and **4** fall outside of the whole metabolic network formed by all the glycosides of *P. chitonoides* not only by the carbohydrate chains but additionally by the aglycone structures.

**Figure 4:**
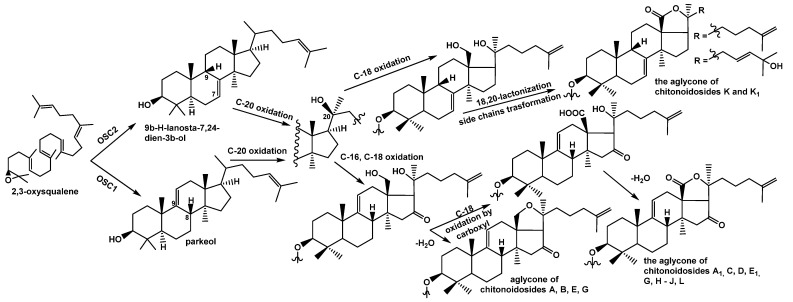
Biosynthesis of the aglycones of the glycosides isolated from *P. chitonoides*.

Indeed, chitonoidosides K (**3**) and K_1_ (**4**) contain aglycones with a 7(8)-double bond instead of 9(11)-bond characteristic for the remaining chitonoidosides (Figure 4). This indicates that the polycyclic systems of the glycosides of *P. chitonoides* can be produced by two different oxidosqualene cyclases (OSCs–enzymes converted 2,3-oxidosqualene into different triterpene alcohols) as was found in *Apostichopus japonicus* [18] at the initial stages of biosynthesis.

Most likely, the OSC1, forming a parkeol (precursor of the glycosides with 9(11)-double bond), is more active or highly expressed than the second one (OSC2), synthetizing 7(8)-unsaturated precursor of the aglycones of chitonoidosides K (**3**) and K_1_ (**4**). Presumably, this branchpoint determines the subsequent general direction of biosynthetic transformations leading to the products **3** and **4**.

Thus, their precursors fall into another cascade of enzymatic reactions, including those catalyzed by cytochrome P450-dependent monooxygenases, oxidating the aglycones so that the stage of C-16 oxidation is absent (Figure 4) but the oxidative transformation of the aglycones side chain is added as well as by glycosyltransferases synthesizing the carbohydrate chains differing from those of the remaining chitonoidosides.

## 3. Materials and Methods

### 3.1. General Experimental Procedures

We used specific rotation with a PerkinElmer 343 Polarimeter (PerkinElmer, Waltham, MA, USA); NMR, Bruker AMX 500 (Bruker BioSpin GmbH, Rheinstetten, Germany) (500.12/125.67 MHz (^1^H/^13^C) spectrometer; ESI MS (positive and negative ion modes), Agilent 6510 Q-TOF apparatus (Agilent Technology, Santa Clara, CA, USA)), sample concentration 0.01 mg/mL; HPLC, Agilent 1260 Infinity II with a differential refractometer (Agilent Technology, Santa Clara, CA, USA); and columns Supelcosil LC-Si (4.6 *×* 150 mm, 5 µm) and Ascentis RP-Amide (10 *×* 250 mm, 5 µm) (Supelco, Bellefonte, PA, USA) and Phenomenex Synergi Fusion RP (10 *×* 250 mm, 5 µm) (Phenomenex, Torrance, CA, USA).

### 3.2. Animals and Cells

Specimens of the holothurian *Psolus chitonoides* (family Psolidae; order Dendrochirotida) were harvested in the Bering Sea during the 14th expedition cruise on board the r/v “Akademik Oparin” on 24 August 1991, north off Bering Island (Commander Islands). The harvesting was performed by the Sigsbee trawl at 100–150 m in depth. The animals were taxonomically determined by Dr. Alexey V. Smirnov, Zoological Institute of the Russian Academy of Sciences. Voucher specimens are kept in Zoological Institute of RAS, St. Petersburg, Russia.

Human erythrocytes were purchased from the Station of Blood Transfusion in Vladivostok. The cells of human adenocarcinoma line HeLa were provided by the N.N. Blokhin National Medicinal Research Center of Oncology of the Ministry of Health Care of the Russian Federation, (Moscow, Russia). The cells of human colorectal adeno- carcinoma line DLD-1 CCL-221™ and human promyeloblast cell line HL-60 CCL-240 were received from ATCC (Manassas, VA, USA).

The HeLa cell line was cultured in the medium of DMEM (Gibco Dulbecco’s Modified Eagle’s Medium) with 1% penicillin/streptomycin sulfate (Biolot, St. Petersburg, Russia) and 10% fetal bovine serum (FBS) (Biolot, St. Petersburg, Russia). The cells of the DLD-1 and HL-60 lines were cultured in the medium of RPMI with 1% penicillin/streptomycin (Biolot, St. Petersburg, Russia) and 10% fetal bovine serum (FBS) (Biolot, St. Petersburg, Russia). All the cells were incubated at 37 °C in a humidified atmosphere at 5% (*v*/*v*) CO_2_.

The study was conducted according to the guidelines of the Declaration of Helsinki and approved by the Ethics Committee of the Pacific Institute of Bioorganic Chemistry (Protocol No. 0037.12.03.2021).

### 3.3. Extraction and Isolation

The sea cucumbers were minced and kept in EtOH at +10 °C. Then, they were extracted twice with refluxing 60% EtOH. The combined extracts were concentrated to dryness in vacuum, dissolved in H_2_O and chromatographed on a Polychrom-1 column (powdered Teflon, Biolar, Latvia). Eluting first the inorganic salts and impurities with H_2_O and then the glycosides with 50% EtOH gave 3200 mg of crude glycoside fraction. After the stepwise column chromatography on Si gel using different ratios of CHCl_3_/EtOH/H_2_O as the mobile phase, some fractions of diverse polarity were obtained.

The most polar fractions V–VII were isolated with CHCl_3_/EtOH/H_2_O in the ratios 100:125:25 and 100:150:50. HPLC of the fraction V on silica-based column Supelcosil LC-Si (4.6 × 150 mm, 5 µm) with CHCl_3_/MeOH/H_2_O (55/30/4) as the mobile phase resulted in the isolation of three subfractions (V.2–V.4). The subsequent HPLC of subfraction V.3 on Supelco Ascentis RP-Amide (10 × 250 mm) with MeOH/H_2_O/NH_4_OAc (1 M water solution) (63/35/2) as the mobile phase led to the isolation of the main subfraction as well as some other minor ones.

The former was repeatedly chromatographed on Supelcosil LC-Si (4.6 *×* 150 mm, 5 µm) with CHCl_3_/MeOH/H_2_O (60/30/4) as the mobile phase and gave 4.9 mg of chitonoidoside I (**1**). For the subfraction V.4, a similar procedure of HPLC was applied: the first step—on a Phenomenex Synergi Fusion RP (10 × 250 mm) column with CH_3_CN/H_2_O/NH_4_OAc (1 M water solution) (32/66/2) as the mobile phase, followed by HPLC of the main fraction on Supelcosil LC-Si (4.6 *×* 150 mm, 5 µm) with CHCl_3_/MeOH/H_2_O (55/23/3) as the mobile phase.

This resulted in the isolation of 4.0 mg of chitonoidoside J (**2**). Chitonoidoside K (**3**) (5.5 mg) was obtained after HPLC of the most polar fraction VII on a Phenomenex Synergi Fusion RP (10 × 250 mm) column with CH_3_CN/H_2_O/NH_4_OAc (1 M water solution) (33/65/2) as the mobile phase followed by the rechromatography of the obtained subfraction VII.4 on Supelco Ascentis RP-Amide (10 × 250 mm) with MeOH/H_2_O/NH_4_OAc (1 M water solution) (65/34/1) as the mobile phase.

#### 3.3.1. Chitonoidoside I (**1**)

Colorless powder; [α]_D_^20^*−*44° (*c* 0.1, 50% MeOH). For NMR: See Table S1 and Table 1, Figures S1–S7. (*−*)HR-ESI-MS *m*/*z*: 768.2694 (calc. 768.2698) [M_2Na_–2Na]^2−^. (*−*)ESI-MS/MS *m*/*z*: 687.2 [M_2Na_–2Na−C_6_H_10_O_5_ (Glc)]^2−^, 621.2 [M_2Na_–2Na−C_6_H_10_O_5_ (Glc)–C_5_H_8_O_4_ (Xyl)]^2−^, 548.2 [M_2Na_–2Na−C_6_H_10_O_5_ (Glc)–C_5_H_8_O_4_ (Xyl)–C_6_H_10_O_4_ (Qui)]^2−^ and 322.0 [M_2Na_–2Na−C_6_H_10_O_5_ (Glc)–C_5_H_8_O_4_ (Xyl)–C_6_H_10_O_4_ (Qui)–C_30_H_44_O_3_ (Agl)]^2−^.

#### 3.3.2. Chitonoidoside J (**2**)

Colorless powder; [α]_D_^20^*−*33° (*c* 0.1, 50% MeOH). NMR: See Table S2 and Table 2, Figures S9–S15. (*−*)HR-ESI-MS *m*/*z*: 1675.4855 (calc. 1675.4832) [M_3Na_–Na]^−^, 826.2495 (calc. 826.2470) [M_3Na_–2Na]^2−^, 543.1700 (calc. 543.1683) [M_3Na_–3Na]^3−^; (*−*)ESI-MS/MS *m*/*z*: 1277.5 [M_3Na_–Na−C_7_H_11_O_8_SNa (MeGlcOSO_3_Na)–NaHSO_4_]^−^, 987.4 [M_3Na_–Na−2C_7_H_11_O_8_SNa (2MeGlcOSO_3_Na)−C_5_H_8_O_4_ (Xyl)]^−^, 841.4 [M_3Na_–Na−2C_7_H_11_O_8_SNa (2MeGlcOSO_3_Na)−C_5_H_8_O_4_ (Xyl)–C_6_H_10_O_4_ (Qui)]^−^; 811.2 [M_3Na_–2Na−OMe]^2−^, 775.3 [M_3Na_–2Na−NaSO_3_]^2−^, 760.3 [M_3Na_–2Na−OMe–NaSO_3_]^2−^ and 687.2 [M_3Na_–2Na−C_7_H_11_O_8_SNa (MeGlcOSO_3_Na)]^2−^ (Figure S16).

#### 3.3.3. Chitonoidoside K (**3**)

Colorless powder; [α]_D_^20^*−*16° (*c* 0.1, 50% MeOH). NMR: See Table 3 and Table 4, Figures S17–S23. (*−*)HR-ESI-MS *m*/*z*: 885.2353 (calc. 885.2320) [M_4Na_–2Na]^2−^, 582.4939 (calc. 582.4916) [M_4Na_–3Na]^3−^, 431.1223 (calc. 431.1214) [M_4Na_–4Na]^4−^; (*−*)ESI-MS/MS *m*/*z*: 665.2 [M_4Na_–2Na−C_30_H_45_O_2_ (Agl)–H]^2−^, 622.2 [M_4Na_–2Na−C_7_H_12_O_8_SNa (MeGlcOSO_3_Na)−C_6_H_9_O_7_SNa (GlcOSO_3_Na)]^2−^, 550.2 [M_4Na_–2Na−C_7_H_12_O_8_SNa (MeGlcOSO_3_Na)−C_6_H_9_O_7_SNa (GlcOSO_3_Na)–C_6_H_9_O_4_ (Qui)]^2−^, 489.8 [M_4Na_–3Na−C_7_H_12_O_8_SNa (MeGlcOSO_3_Na)]^3−^ and 385.1 [M_4Na_–3Na−2C_7_H_12_O_9_SNa (MeGlcOSO_3_Na)]^3−^ (Figure S24).

#### 3.3.4. Chitonoidoside K_1_ (**4**)

Colorless powder; [α]_D_^20^*−*20° (*c* 0.1, 50% MeOH). NMR: See Table 3 and Table 5, Figures S25–S31. (*−*)HR-ESI-MS *m*/*z*: 893.2272 (calc. 893.2295) [M_4Na_–2Na]^2−^, 587.8228 (calc. 587.8232) [M_4Na_–3Na]^3−^, 435.1200 (calc. 435.1210) [M_4Na_–4Na]^4−^; (*−*)ESI-MS/MS *m*/*z*: 754.2 [M_4Na_–2Na−C_7_H_12_O_8_SNa (MeGlcOSO_3_Na)]^2−^, 622.2 [M_4Na_–2Na−C_7_H_12_O_8_SNa (MeGlcOSO_3_Na)−C_6_H_9_O_8_SNa (GlcOSO_3_Na)]^2−^ and 495.2 [M_4Na_–3Na−C_7_H_12_O_8_SNa (MeGlcOSO_3_Na)]^3−^ (Figure S32).

#### 3.3.5. Chitonoidoside L (**5**)

Colorless powder; [α]_D_^20^*−*38° (*c* 0.1, 50% MeOH). NMR: See Table S2 and Table 6, Figures S33–S39. (*−*)HR-ESI-MS *m*/*z*: 892.2234 (calc. 892.2217) [M_4Na_–2Na]^2−^, 587.1535 (calc. 587.1510) [M_4Na_–3Na]^3−^, 434.6182 (calc. 434.6160) [M_4Na_–4Na]^4−^; (*−*)ESI-MS/MS *m*/*z*: 621.2 [M_4Na_–2Na−C_7_H_12_O_8_SNa (MeGlcOSO_3_Na)−C_6_H_9_O_8_SNa (GlcOSO_3_Na)]^2−^, 548.2 [M_4Na_–2Na−C_7_H_12_O_8_SNa (MeGlcOSO_3_Na)−C_6_H_9_O_8_SNa (GlcOSO_3_Na)–C_6_H_9_O_4_ (Qui)]^2−^ and 494.8 [M_4Na_–3Na−C_7_H_12_O_8_SNa (MeGlcOSO_3_Na)]^3−^ (Figure S40).

### 3.4. Cytotoxic Activity (MTT Assay) (for DLD-1 Cells)

All the studied substances (including chitonoidoside A and cisplatin used as positive controls) were tested in concentrations between 0.1 to 100 µM using two-fold dilution in d-H_2_O. The cell suspension (180 µL) and solutions (20 µL) of tested compounds in different concentrations were injected into wells of 96-well plates (1 × 10^4^ cells/well) and incubated at 37 °C for 24 h in a 5% CO_2_ atmosphere. After incubation, the tested substances with medium were replaced by 100 µL of fresh medium. Then, 10 µL of MTT (3-(4,5-dimethylthiazol-2-yl)-2,5-diphenyltetrazolium bromide) (Sigma-Aldrich, St. Louis, MO, USA) stock solution (5 mg/mL) was added to each well, followed by incubation of the microplate for 4 h.

After that, 100 µL of SDS-HCl solution (1 g SDS/10 mL d-H_2_O/17 µL 6 N HCl) was added to each well, and the plates were incubated for 18 h. The absorbance of the converted dye formazan was measured with a Multiskan FC microplate photometer (Thermo Fisher Scientific, Waltham, MA, USA) at 570 nm. The cytotoxic activity of the tested compounds was calculated as the concentration that caused 50% cell metabolic activity inhibition (IC_50_). The experiments were performed in triplicate, *p* < 0.05.

### 3.5. Cytotoxic Activity (MTS Assay) (for HeLa and HL-60 Cells)

The cells of the HL-60 line (6 × 10^3^/200 µL) were placed in 96-well plates at 37 °C for 24 h in a 5% CO_2_ incubator. The cells were treated with tested substances and chitonoidoside A and cisplatin as positive control at concentrations from 0 to 100 µM for an additional 24 h incubation.

Then, the cells were incubated with 10 µL MTS ([3-(4,5-dimethylthiazol-2-yl)-5-(3-carboxymethoxyphenyl)-2-(4-sulfophenyl)-2H-tetrazolium) for 4 h, and the absorbance in each well was measured at 490/630 nm with plate reader PHERA star FS (BMG Labtech, Ortenberg, Germany). The experiments were performed in triplicate, and the mean absorbance values were calculated. The results are presented as the percentage of inhibition that produced a reduction in absorbance after the tested compounds treatment compared to the non-treated cells (negative control), *p* < 0.01.

### 3.6. Hemolytic Activity

Erythrocytes were isolated from human blood (A(+)) by centrifugation with phosphate-buffered saline (PBS) (pH 7.4) at 4 °C for 5 min by 450 g on a LABOFUGE 400R (Heraeus, Hanau, Germany) centrifuge three times. Then, the residue of erythrocytes was resuspended in ice cold phosphate saline buffer (pH 7.4) to a final optical density of 1.5 at 700 nm and kept on ice. For the hemolytic assay, 180 µL of erythrocyte suspension was mixed with 20 µL of test compound solution (including chitonoidoside A used as positive control) in V-bottom 96-well plates.

After 1 h of incubation at 37 °C, the plates were exposed to centrifugation 10 min at 900 g on a LMC-3000 (Biosan, Riga, Latvia) laboratory centrifuge. Then, 100 µL of supernatant was carefully selected and transferred to new flat-plates, respectively. Lysis of erythrocytes was determined by measuring the concentration of hemoglobin in the supernatant with a microplate photometer Multiskan FC (Thermo Fisher Scientific, Waltham, MA, USA), λ = 570 nm. The effective dose causing 50% hemolysis of erythrocytes (ED_50_) was calculated using the computer program SigmaPlot 10.0. All the experiments were made in triplicate, *p* < 0.01.

## 4. Conclusions

As result of the investigation of the glycosidic composition of the sea cucumber *Psolus chitonoides,* fifteen previously unknown triterpene glycosides—chitonoidosides A–L—were isolated, their structures were established, and their cytotoxic activity was studied. Four different aglycones were found in them: two of them characterized by 9(11)-double bonds in the polycyclic system (holotoxinogenin and its structural analog with an 18(20)-ether bond), and the other two had 7(8)-unsaturated nuclei and different side chains (Figure 2).

These findings suggest that two oxidosqualene cyclases operate the biosynthesis of these aglycones, forming parkeol type and 9*β*H-lanosta-7,24-diene precursors. Twelve structurally different carbohydrate chains were found in the glycosides of *P. chitonoides* (groups of chitonoidosides A–L).

Among them are tetrasaccharide chains of three types (chitonoidosides of the groups A, C and F), differing by their architecture (having/lacking the bottom/upper semi-chain), sugar composition and the number of sulfate groups; and two types of pentasaccharide chains (chitonoidosides of the groups D and H), whose architectures were quite different due to the absence or presence of one of the terminal (fourth or sixth) 3-*O*-methylglucose residues sulfated by C-4 or by C-6.

The major part of the glycosidic sum consists of the glycosides with hexasaccharide moieties (seven types: chitonoidosides of the groups B, E, G and I–L). They were variable in the third residue in the chain (Xyl3 or Glc3), the quantity of sulfate groups (from one to four) as well as their positions and finally by terminal monosaccharides in the bottom (MeGlc4 or Glc4) and upper (MeXyl6 or MeGlc6) semi-chains.

Noticeably, the hexaosides, chitonoidosides D (58 mg) and K_1_ (**4**) (53 mg), were quantitatively predominant compounds of the glycosidic sum of *P. chitonoides*. This fact is easily explained by the high biologic activity of chitonoidoside D [23]. However, it is a surprise in the case of **4** because its activity was greatly reduced by the presence of an OH-group in the side chain [22]. It can be supposed that this highly polar and hydrophilic compound performs an ecological protective function in acting as an aposematic signal to prevent predator attacks.

The unusual structural features of the carbohydrate chains of chitonoidosides were 3-*O*-methylxylose and 3-*O*-methylglucose residues sulfated by C-4 and the presence of four sulfate groups attached to the C-6 of different glucopyranose units.

The pathways of biosynthetic transformations of the both aglycones and carbohydrate chains were analyzed based on the structural features of the glycosides of *P. chitonoides*. The analysis revealed and confirmed the mosaicism of the biosynthesis of these compounds with some clearly directed trends.

## Figures and Tables

**Table 1:** ^13^C and ^1^H NMR chemical shifts and HMBC and ROESY correlations of carbohydrate moiety of chitonoidoside I (**1**).

Atom	δ_C_ Mult. *^a, b, c^*	δ_H_ Mult. (*J* in Hz) *^d^*	HMBC	ROESY
Xyl1 (1→C-3)				
1	104.7 CH	4.66 d (6.3)	C: 3	H-3; H-3, 5 Xyl1
2	**82.1** CH	3.93 t (8.1)	C: 1 Qui2; 1 Xyl1	H-1 Qui2
3	75.1 CH	4.13 t (8.1)	C: 4 Xyl1	H-1, 5 Xyl1
4	**78.3** CH	4.13 m		H-1 Glc5
5	63.5 CH_2_	4.37 brd (11.3)		
		3.64 m		H-1, 3 Xyl1
Qui2 (1→2Xyl1)				
1	104.5 CH	5.02 d (7.3)	C: 2 Xyl1	H-2 Xyl1; H-3, 5 Qui2
2	75.7 CH	3.88 t (8.9)	C: 1, 3 Qui2	H-4 Qui2
т3	74.7 CH	4.00 t (8.9)	C: 2, 4 Qui2	H-1, 5 Qui2
4	**85.6** CH	3.48 t (8.9)	C: 1 Xyl3; 3, 5 Qui2	H-1 Xyl3; H-2 Qui2
5	71.4 CH	3.68 dd (6.2; 8.9)		H-1, 3 Qui2
6	17.7 CH_3_	1.60 d (6.2)	C: 4, 5 Qui2	H-4, 5 Qui2
Xyl3 (1→4Qui2)				
1	104.3 CH	4.75 d (7.6)	C: 4 Qui2	H-4 Qui2; H-3, 5 Xyl3
2	73.3 CH	3.87 t (8.7)	C: 1, 3 Xyl3	
3	**86.5** CH	4.10 t (8.7)	C: 1 Glc4; 2 Xyl3	H-1 Glc4; H-1, 5 Xyl3
4	68.7 CH	3.93 m		
5	65.8 CH_2_	4.13 m	C: 4 Xyl3	
		3.60 m	C: 1 Xyl3	H-1 Xyl3
Glc4 (1→3Xyl3)				
1	104.4 CH	5.20 d (7.7)	C: 3 Xyl3	H-3 Xyl3; H-3, 5 Glc4
2	74.9 CH	3.93 t (8.6)	C: 1, 3 Glc4	
3	77.2 CH	4.10 m	C: 4 Glc4	H-1 Glc4
4	71.1 CH	3.90 m	C: 5 Glc4	H-6 Glc4
5	77.7 CH	3.91 m		H-1 Glc4
6	62.0 CH_2_	4.39 d (11.2)		
		4.04 dd (5.4; 11.2)	C: 5 Glc4	
Glc5 (1→4Xyl1)				
1	102.4 CH	4.88 d (8.2)	C: 4 Xyl1	H-4 Xyl1; H-3, 5 Glc5
2	73.6 CH	3.82 t (8.8)	C: 1, 3 Glc5	
3	**85.9** CH	4.16 t (8.8)	C: 1 MeGlc6	H-1 MeGlc6; H-1, 5 Glc5
4	69.0 CH	3.85 t (8.8)		
5	75.5 CH	4.05 t (8.8)		H-1 Glc5
6	*67.3* CH_2_	4.92 d (11.3)		
		4.64 dd (6.2; 11.3)		
MeGlc6 (1→3Glc5)				
1	104.4 CH	5.17 d (7.6)	C: 3 Glc5	H-3 Glc5; H-3, 5 MeGlc6
2	74.0 CH	3.85 t (8.9)	C: 1 MeGlc6	
3	85.2 CH	3.70 t (8.9)	C: 4 MeGlc6, OMe	H-1 MeGlc6
4	*76.1* CH	4.88 t (8.9)	C: 5 MeGlc6	H-2 MeGlc6
5	76.3 CH	3.84 m		H-1 MeGlc6
6	61.6 CH_2_	4.48 d (11.4)		
		4.32 dd (5.1; 11.4)		
OMe	60.7 CH_3_	3.92 s	C: 3 MeGlc6	H-3 MeGlc6

*^a^* Recorded at 125.67 MHz in C_5_D_5_N/D_2_O (4/1). *^b^* Bold = interglycosidic positions. *^c^* Italic = sulfate position. *^d^* Recorded at 500.12 MHz in C_5_D_5_N/D_2_O (4/1). Multiplicity by 1D TOCSY. The original spectra of **1** are provided as Figures S1–S7.

**Table 2:** ^13^C and ^1^H NMR chemical shifts and HMBC and ROESY correlations of carbohydrate moiety of chitonoidoside J (**2**).

Atom	δ_C_ Mult. *^a, b, c^*	δ_H_ Mult. (*J* in Hz) *^d^*	HMBC	ROESY
Xyl1 (1→C-3)				
1	104.5 CH	4.66 d (6.7)	C: 3	H-3; H-3, 5 Xyl1
2	**82.4** CH	3.88 t (6.7)	C: 1 Qui2; 1, 3 Xyl1	H-1 Qui2
3	75.0 CH	4.07 t (6.7)	C: 4 Xyl1	H-5 Xyl1
4	**78.6** CH	4.07 m		H-1 Glc5
5	63.3 CH_2_	4.33 brd (10.7)	C: 3 Xyl1	
		3.59 t (10.7)		H-1 Xyl1
Qui2 (1→2Xyl1)				
1	104.5 CH	4.97 d (7.3)	C: 2 Xyl1	H-2 Xyl1; H-3, 5 Qui2
2	75.5 CH	3.93 t (7.9)	C: 1, 3 Qui2	H-4 Qui2
3	74.2 CH	4.04 t (7.9)	C: 2, 4 Qui2	H-5 Qui2
4	**85.0** CH	3.48 t (7.9)	C: 1 Xyl3; 3, 5 Qui2	H-1 Xyl3
5	71.2 CH	3.68 dd (6.1; 8.0)		H-1 Qui2
6	17.3 CH_3_	1.62 d (6.2)	C: 4, 5 Qui2	H-4, 5 Qui2
Xyl3 (1→4Qui2)				
1	104.0 CH	4.74 d (7.2)	C: 4 Qui2	H-4 Qui2; H-3, 5 Xyl3
2	73.0 CH	3.85 t (9.0)	C: 1, 3 Xyl3	
3	**86.4** CH	4.06 t (9.0)	C: 1 MeGlc4; 2 Xyl3	H-1 MeGlc4; H-5 Xyl3
4	68.2 CH	3.92 t (9.0)		
5	65.6 CH_2_	4.12 dd (4.8; 10.8)	C: 3, 4 Xyl3	
		3.59 t (10.8)	C: 1 Xyl3	H-1 Xyl3
MeGlc4 (1→3Xyl3)				
1	104.5 CH	5.14 d (7.6)	C: 3 Xyl3	H-3 Xyl3; H-3, 5 MeGlc4
2	73.9 CH	3.84 t (8.6)	C: 1 MeGlc4	
3	86.4 CH	3.62 t (8.6)	C: 2,4 MeGlc4; OMe	H-1 MeGlc4
4	69.7 CH	4.04 m	C: 5 MeGlc4	
5	75.5 CH	4.04 m		H-1, 3 MeGlc4
6	66.6 CH_2_	5.02 d (11.3)		
		4.83 dd (4.9; 11.3)		H-4 MeGlc4
OMe	60.1 CH_3_	3.76 s	C: 3 MeGlc4	
Glc5 (1→4Xyl1)				
1	102.7 CH	4.86 d (7.6)	C: 4 Xyl1	H-4 Xyl1; H-3, 5 Glc5
2	72.8 CH	3.83 t (7.6)	C: 1 Glc5	
3	**86.0** CH	4.13 t (8.9)	C: 1 MeGlc6; 2, 4 Glc5	H-1 MeGlc6; H-1 Glc5
4	68.9 CH	3.83 m		
5	75.5 CH	4.07 t (8.9)		H-1 Glc5
6	*67.0* CH_2_	5.04 d (11.4)		
		4.67 m		
MeGlc6 (1→3Glc5)				
1	104.3 CH	5.19 d (7.6)	C: 3 Glc5	H-3 Glc5; H-3, 5 MeGlc6
2	73.8 CH	3.89 t (8.9)	C: 1, 3 MeGlc6	
3	85.2 CH	3.76 t (8.9)	C: 2, 4 MeGlc6; OMe	
4	*75.5* CH	5.07 t (8.9)	C: 3 MeGlc6	H-2 MeGlc6
5	76.4 CH	3.85 t (8.9)		H-1 MeGlc6
6	61.3 CH_2_	4.48 dd (2.5; 11.4)		
		4.43 brd (11.4)		
OMe	60.2 CH_3_	3.97 s	C: 3 MeGlc6	H-3 MeGlc6

*^a^* Recorded at 125.67 MHz in C_5_D_5_N/D_2_O (4/1). *^b^* Bold = interglycosidic positions. *^c^* Italic = sulfate position. *^d^* Recorded at 500.12 MHz in C_5_D_5_N/D_2_O (4/1). Multiplicity by 1D TOCSY. The original spectra of **2** are provided as Figures S9–S15.

**Table 3:** ^13^C and ^1^H NMR chemical shifts and HMBC and ROESY correlations of carbohydrate moiety of chitonoidoside K (**3**).

Atom	δ_C_ Mult. *^a, b, c^*	δ_H_ Mult. (*J* in Hz) *^d^*	HMBC	ROESY
Xyl1 (1→C-3)				
1	104.6 CH	4.57 d (7.3)	C: 3	H-3; H-3, 5 Xyl1
2	**82.9** CH	3.69 t (8.7)	C: 1 Qui2; 1, 3 Xyl1	H-1 Qui2
3	75.1 CH	3.92 m	C: 4 Xyl1	H-1, 5 Xyl1
4	**80.3** CH	3.93 m		H-1 Glc5
5	63.4 CH_2_	4.27 dd (4.7; 12.1)	C: 3 Xyl1	
		3.52 m		H-1 Xyl1
Qui2 (1→2Xyl1)				
1	104.6 CH	4.72 d (8.6)	C: 2 Xyl1	H-2 Xyl1; H-3, 5 Qui2
2	75.5 CH	3.84 t (8.6)	C: 1, 3 Qui2	H-4 Qui2
3	74.4 CH	3.95 t (8.6)	C: 2, 4 Qui2	
4	**86.1** CH	3.25 t (8.6)	C: 1 Glc3; 3, 5 Qui2	H-1 Glc3; H-2 Qui2
5	71.6 CH	3.53 dd (5.7; 8.6)		H-1, 3 Qui2
6	17.5 CH_3_	1.48 d (5.7)	C: 4, 5 Qui2	H-4, 5 Qui2
Glc3 (1→4Qui2)				
1	103.9 CH	4.64 d (7.9)	C: 4 Qui2	H-4 Qui2; H-3, 5 Glc3
2	74.5 CH	3.73 t (9.1)	C: 1, 3 Glc3	
3	**86.3** CH	4.10 t (9.1)	C: 1 MeGlc4; 4 Glc3	H-1 MeGlc4; H-1 Glc3
4	69.2 CH	3.64 t (9.1)	C: 3, 5, 6 Glc3	
5	74.3 CH	4.04 t (9.1)		H-1 Glc3
6	*67.5* CH_2_	4.87 dd (4.2; 11.0)		
		4.49 dd (6.9; 11.0)	C: 5 Glc3	
MeGlc4 (1→3Glc3)				
1	104.6 CH	5.11 d (8.0)	C: 3 Glc3	H-3 Glc3; H-3, 5 MeGlc4
2	74.2 CH	3.74 t (8.0)	C: 1, 3 MeGlc4	
3	86.4 CH	3.58 t (8.0)	C: 4 MeGlc4, OMe	H-1, 5 MeGlc4; OMe
4	69.8 CH	3.96 t (8.0)	C: 3, 5 MeGlc4	H-6 MeGlc4
5	75.5 CH	3.96 m	C: 6 MeGlc4	H-1, 3 MeGlc4
6	*67.0* CH_2_	4.88 d (11.6)		
		4.69 dd (3.5; 11.6)	C: 5 MeGlc4	
OMe	60.5 CH_3_	3.70 s	C: 3 MeGlc4	
Glc5 (1→4Xyl1)				
1	103.2 CH	4.80 d (8.4)	C: 4 Xyl1	H-4 Xyl1; H-3, 5 Glc5
2	73.2 CH	3.77 t (8.4)	C: 1 Glc5	
3	**86.2** CH	4.08 t (9.1)	C: 1 MeGlc6; 2 Glc5	H-1 MeGlc6; H-1 Glc5
4	69.4 CH	3.67 t (9.1)		
5	74.4 CH	4.07 m		
6	*67.7* CH_2_	4.90 d (11.0)		
		4.43 dd (7.8; 11.0)	C: 5 Glc5	
MeGlc6 (1→3Glc5)				
1	104.5 CH	5.07 d (7.8)	C: 3 Glc5	H-3 Glc5; H-3, 5 MeGlc6
2	74.3 CH	3.72 t (7.8)	C: 1, 3 MeGlc6	
3	86.4 CH	3.57 t (7.8)	C: 4 MeGlc6, OMe	H-1, 5 MeGlc6
4	69.8 CH	3.95 m	C: 3, 5 MeGlc6	H-6 MeGlc6
5	75.5 CH	3.95 m		H-1, 3 MeGlc6
6	*67.0* CH_2_	4.87 d (11.0)	C: 4, 5 MeGlc6	
		4.69 dd (2.6; 11.0)		
OMe	60.5 CH_3_	3.71 s	C: 3 MeGlc6	

*^a^* Recorded at 125.67 MHz in C_5_D_5_N/D_2_O (4/1). *^b^* Bold = interglycosidic positions. *^c^* Italic = sulfate position. *^d^* Recorded at 500.12 MHz in C_5_D_5_N/D_2_O (4/1). Multiplicity by 1D TOCSY. The original spectra of **1** are provided Figures S17–S23.

**Table 4:** ^13^C and ^1^H NMR chemical shifts and HMBC and ROESY correlations of the aglycone moiety of chitonoidoside K (**3**).

Position	δ_C_ Mult. *^a^*	δ_H_ Mult. (*J* in Hz) *^b^*	HMBC	ROESY
1	36.0 CH_2_	1.30 m (α)		H-3, H-5, H-11, H-19
2	26.9 CH_2_	1.95 m (β)		
		1.76 m		
3	88.9 CH	3.14 dd (3.3; 11.8)		H-1, H-5, H-31, H1-Xyl1
4	39.2 C			
5	48.0 CH	0.88 m	C: 10, 19, 30	H-3, H-31
6	22.9 CH_2_	1.89 m		H-31
7	119.8 CH	5.64 m		H-15, H-32
8	146.7 C			
9	47.3 CH	3.32 brd (14.1)		H-19
10	35.4 C			
11	22.7 CH_2_	1.65 m (α)		H-1
		1.42 m (β)		H-19
12	30.2 CH_2_	1.95 m		
13	58.7 C			
14	51.2 C			
15	34.1 CH_2_	1.73 m (β)		H-7, H-32
		1.49 d (13.1) (α)		H-32
16	24.4 CH_2_	1.93 m (β)		
		1.72 m (α)		H-32
17	53.0 CH	2.17 t (12.3)		H-21, H-32
18	180.9 C			
19	23.9 CH_3_	1.08 s	C: 5, 9, 10	H-2, H-9
20	84.7 C			
21	25.9 CH_3_	1.38 s	C: 17, 20, 22	H-12, H-17, H-24
22	39.2 CH_2_	1.64 m		
		1.54 m		
23	21.8 CH_2_	1.31 m		
		1.23 m		
24	37.8 CH_2_	1.88 m	C: 25	
25	145.3 C			
26	110.7 CH_2_	4.70 brs	C: 24, 27	H-27
		4.66 brs	C: 24, 27	H-24
27	22.0 CH_3_	1.61 s	C: 24, 25, 26	H-24, H-26
30	17.1 CH_3_	0.89 s	C: 3, 4, 5, 31	H-31
31	28.5 CH_3_	1.09 s	C: 3, 4, 5, 30	H-3, H-5, H-6, H-30, H-1 Xyl1
32	30.8 CH_3_	1.08 s	C: 8, 13, 14, 15	H-7, H-12, H-17

*^a^* Recorded at 125.67 MHz in C_5_D_5_N/D_2_O (4/1). *^b^* Recorded at 500.12 MHz in C_5_D_5_N/D_2_O (4/1). The original spectra of **3** are provided as Figures S17–S22.

**Table 5:** ^13^C and ^1^H NMR chemical shifts and HMBC and ROESY correlations of the aglycone moiety of chitonoidoside K_1_ (**4**).

Position	δ_C_ Mult. *^a^*	δ_H_ Mult. (*J* in Hz) *^b^*	HMBC	ROESY
1	36.7 CH_2_	1.36 m		H-31
2	27.6 CH_2_	2.01 m		
		1.81 m		H-19, H-30
3	89.6 CH	3.19 dd (3.5; 11.5)		H-5, H-31, H1-Xyl1
4	40.0 C			
5	48.7 CH	0.92 m	C: 6, 10, 30	H-1, H-3, H-31
6	23.8 CH_2_	1.94 m		H-31
7	120.5 CH	5.67 m		H-15, H-32
8	147.4 C			
9	48.1 CH	3.35 brd (14.4)		H-19
10	36.1 C			
11	23.5 CH_2_	1.93 m		
		1.70 m		
12	30.9 CH_2_	1.95 m		
13	59.5 C			
14	51.9 C			
15	34.9 CH_2_	1.77 m		H-7
		1.54 m		H-32
16	25.3 CH_2_	2.03 m		H-32
		1.88 m		
17	53.8 CH	2.25 dd (4.8; 9.6)		H-12, H-21, H-32
18	181.7 C			
19	24.6 CH_3_	1.13 s	C: 1, 5, 9, 10	H-2, H-6, H-9
20	85.2 C			
21	26.8 CH_3_	1.42 s	C: 17, 20, 22	H-12, H-17, H-22
22	42.7 CH_2_	2.43 brt (7.0)		
23	120.8 CH	5.78 dt (7.1; 15.0)	C: 22, 25	H-26 (27)
24	144.7 CH	5.91 d (15.0)	C: 22, 25, 26, 27	H-26 (27)
25	70.7 C			
26	30.6 CH_3_	1.48 s	C: 24, 25, 27	
27	30.6 CH_3_	1.48 s	C: 24, 25, 26	
30	18.3 CH_3_	0.93 s	C: 3, 4, 5, 31	H-2, H-6, H-31
31	29.2 CH_3_	1.13 s	C: 3, 4, 5, 30	H-3, H-6, H-30, H-1 Xyl1
32	31.5 CH_3_	1.06 s	C: 8, 13, 14, 15	H-7, H-12, H-15, H-16, H-17

*^a^* Recorded at 125.67 MHz in C_5_D_5_N/D_2_O (4/1). *^b^* Recorded at 500.12 MHz in C_5_D_5_N/D_2_O (4/1). The original spectra of **4** are provided as Figures S25–S30.

**Table 6:** ^13^C and ^1^H NMR chemical shifts and HMBC and ROESY correlations of carbohydrate moiety of chitonoidoside L (**5**).

Atom	δ_C_ Mult. *^a, b, c^*	δ_H_ Mult. (*J* in Hz) *^d^*	HMBC	ROESY
Xyl1 (1→C-3)				
1	104.6 CH	4.62 d (7.5)	C: 3	H-3; H-3, 5 Xyl1
2	**82.8** CH	3.78 m	C: 1 Qui2; 1, 3 Xyl1	H-1 Qui2; H-4 Xyl1
3	75.1 CH	4.00 m	C: 4 Xyl1	H-1, 5 Xyl1
4	**80.0** CH	4.01 m	C: 3 Xyl1	H-1 Glc5
5	63.5 CH_2_	4.33 dd (5.3; 11.7)		
		3.58 m		H-1 Xyl1
Qui2 (1→2Xyl1)				
1	104.6 CH	4.81 d (7.5)	C: 2 Xyl1	H-2 Xyl1; H-3, 5 Qui2
2	75.5 CH	3.88 t (9.5)	C: 3 Qui2	H-4 Qui2
3	74.5 CH	3.99 t (9.5)	C: 2 Qui2	H-1, 5 Qui2
4	**86.4** CH	3.33 t (9.5)	C: 1 Glc3	H-1 Glc3; H-2 Qui2
5	71.5 CH	3.61 dd (5.9; 9.6)		H-1, 3 Qui2
6	17.5 CH_3_	1.55 d (5.9)	C: 4, 5 Qui2	H-4, 5 Qui2
Glc3 (1→4Qui2)				
1	104.0 CH	4.70 d (7.5)	C: 4 Qui2	H-4 Qui2; H-3, 5 Glc3
2	74.5 CH	3.80 t (7.5)	C: 1 Glc3	
3	**86.1** CH	4.16 t (7.5)	C: 1 MeGlc4; 4 Glc3	H-1 MeGlc4; H-1 Glc3
4	69.2 CH	3.71 t (7.5)	C: 3, 6 Glc3	H-2, 6 Glc3
5	74.6 CH	4.10 m		H-1 Glc3
6	*67.6* CH_2_	4.94 brd (10.7)	C: 4 Glc3	
		4.54 dd (7.1; 10.7)	C: 5 Glc3	H-4 Glc3
MeGlc4 (1→3Glc3)				
1	104.6 CH	5.16 d (8.4)	C: 3 Glc3	H-3 Glc3; H-3, 5 MeGlc4
2	74.3 CH	3.80 t (8.4)	C: 1, 3 MeGlc4	
3	86.3 CH	3.63 t (8.4)	C: 2, 4 MeGlc4, OMe	H-1, 5 MeGlc4; OMe
4	69.8 CH	4.00 m	C: 3 MeGlc4	H-6 MeGlc4
5	75.5 CH	4.00 m	C: 6 MeGlc4	H-1 MeGlc4
6	*67.0* CH_2_	4.93 d (11.7)	C: 5 MeGlc4	
		4.75 d (11.7)	C: 5 MeGlc4	
OMe	60.4 CH_3_	3.75 s	C: 3 MeGlc4	
Glc5 (1→4Xyl1)				
1	103.1 CH	4.86 d (8.5)	C: 4 Xyl1	H-4 Xyl1; H-3, 5 Glc5
2	73.3 CH	3.83 t (8.5)	C: 1, 3 Glc5	
3	**86.4** CH	4.17 t (8.5)	C: 1 MeGlc6; 4 Glc5	H-1 MeGlc6; H-1 Glc5
4	69.3 CH	3.74 t (8.5)	C: 3, 5, 6 Glc5	
5	74.5 CH	4.12 m		H-1 Glc5
6	*67.5* CH_2_	4.95 d (11.8)		
		4.51 m		
MeGlc6 (1→3Glc5)				
1	104.3 CH	5.17 d (7.4)	C: 3 Glc5	H-3 Glc5; H-3, 5 MeGlc6
2	74.0 CH	3.87 t (8.2)	C: 1, 3 MeGlc6	H-4 MeGlc6
3	85.3 CH	3.72 t (8.2)	C: 2, 4 MeGlc6, OMe	H-1 MeGlc6; OMe
4	*76.1* CH	4.88 t (9.3)	C: 3, 5, 6 MeGlc6	H-2 MeGlc6
5	76.5 CH	3.85 m		H-1 MeGlc6
6	61.7 CH_2_	4.50 d (10.7)		
		4.33 dd (6.6; 11.5)		
OMe	60.7 CH_3_	3.92 s	C: 3 MeGlc6	

*^a^* Recorded at 125.67 MHz in C_5_D_5_N/D_2_O (4/1). *^b^* Bold = interglycosidic positions. *^c^* Italic = sulfate position. *^d^* Recorded at 500.12 MHz in C_5_D_5_N/D_2_O (4/1). Multiplicity by 1D TOCSY. The original spectra of **5** are provided Figures S33–S39.

**Table 7:** The cytotoxic activities of glycosides **1**–**5**, cisplatin and chitonoidoside A (positive controls) against human erythrocytes, HeLa, DLD-1 and HL-60 human cell lines.

Glycosides	ED_50_, µM, Erythrocytes	Cytotoxicity, ED_50_ µM		
		HeLa	DLD-1	HL-60
Chitonoidoside I (**1**)	1.26 ± 0.09	20.56 ± 1.06	69.12 ± 5.12	17.50 ± 0.95
Chitonoidoside J (**2**)	2.48 ± 0.13	17.64 ± 0.70	58.17 ± 4.27	10.17 ± 0.89
Chitonoidoside K (**3**)	0.29 ± 0.01	13.82 ± 1.10	9.20 ± 0.21	6.77 ± 0.44
Chitonoidoside K_1_ (**4**)	>100.00	>100.00	>100.00	>100.00
Chitonoidoside L (**5**)	0.71 ± 0.03	15.21 ± 1.45	16.45 ± 0.51	7.89 ± 0.44
Chitonoidoside A	7.51 ± 0.11	45.36 ± 1.05	39.67 ± 1.36	12.83 ± 0.87
Cisplatin	-	23.82 ± 1.81	>100.00	6.77 ± 0.14

## Supplementary Materials

The following supporting information can be downloaded at: https://www.mdpi.com/article/10.3390/md20060369/s1, Figure S1. The ^13^C NMR (125.67 MHz) spectrum of chitonoidoside I (**1**) in C_5_D_5_N/D_2_O (4/1); Figure S2. The ^1^H NMR (500.12 MHz) spectrum of chitonoidoside I (**1**) in C_5_D_5_N/D_2_O (4/1); Figure S3. The COSY (500.12 MHz) spectrum of chitonoidoside I (**1**) in C_5_D_5_N/D_2_O (4/1); Figure S4. The HSQC (500.12 MHz) spectrum of chitonoidoside I (**1**) in C_5_D_5_N/D_2_O (4/1); Figure S5. The ROESY (500.12 MHz) spectrum of chitonoidoside I (**1**) in C_5_D_5_N/D_2_O (4/1); Figure S6. The HMBC (500.12 MHz) spectrum of chitonoidoside I (**1**) in C_5_D_5_N/D_2_O (4/1); Figure S7. 1 D TOCSY (500.12 MHz) spectra of Xyl1, Qui2, Xyl3, Glc4, Glc5 and MeGlc6 of chitonoidoside I (**1**) in C_5_D_5_N/D_2_O (4/1); Figure S8. HR-ESI-MS and ESI-MS/MS spectra of chitonoidoside I (**1**); Figure S9. The ^13^C NMR (125.67 MHz) spectrum of chitonoidoside J (**2**) in C_5_D_5_N/D_2_O (4/1); Figure S10. The ^1^H NMR (500.12 MHz) spectrum of chitonoidoside J (**2**) in C_5_D_5_N/D_2_O (4/1); Figure S11. The COSY (500.12 MHz) spectrum of chitonoidoside J (**2**) in C_5_D_5_N/D_2_O (4/1); Figure S12. The HSQC (500.12 MHz) spectrum of chitonoidoside J (**2**) in C_5_D_5_N/D_2_O (4/1); Figure S13. The HMBC (500.12 MHz) spectrum of chitonoidoside J (**2**) in C_5_D_5_N/D_2_O (4/1); Figure S14. The ROESY (500.12 MHz) spectrum of chitonoidoside J (**2**) in C_5_D_5_N/D_2_O (4/1); Figure S15. 1D TOCSY (500.12 MHz) spectra of Xyl1, Qui2, Xyl3, MeGlc4, Glc5, MeGlc6 of chitonoidoside J (**2**) in C_5_D_5_N/D_2_O (4/1); Figure S16. HR-ESI-MS and ESI-MS/MS spectra of chitonoidoside J (**2**); Figure S17. The ^13^C NMR (125.67 MHz) spectrum of chitonoidoside K (**3**) in C_5_D_5_N/D_2_O (4/1); Figure S18. The ^1^H NMR (500.12 MHz) spectrum of chitonoidoside K (**3**) in C_5_D_5_N/D_2_O (4/1); Figure S19. The COSY (500.12 MHz) spectrum of chitonoidoside K (**3**) in C_5_D_5_N/D_2_O (4/1); Figure S20. The HSQC (500.12 MHz) spectrum of chitonoidoside K (**3**) in C_5_D_5_N/D_2_O (4/1); Figure S21. The HMBC (500.12 MHz) spectrum of chitonoidoside K (**3**) in C_5_D_5_N/D_2_O (4/1); Figure S22. The ROESY (500.12 MHz) spectrum of chitonoidoside K (**3**) in C_5_D_5_N/D_2_O (4/1); Figure S23. 1D TOCSY (500.12 MHz) spectra of Xyl1, Qui2, Glc3, MeGlc4, Glc5, MeGlc6 of chitonoidoside K (**3**) in C_5_D_5_N/D_2_O (4/1); Figure S24. HR-ESI-MS and ESI-MS/MS spectra of chitonoidoside K (**3**); Figure S25. The ^13^C NMR (125.67 MHz) spectrum of chitonoidoside K_1_ (**4**) in C_5_D_5_N/D_2_O (4/1); Figure S26. The ^1^H NMR (500.12 MHz) spectrum of chitonoidoside K_1_ (**4**) in C_5_D_5_N/D_2_O (4/1); Figure S27. The COSY (500.12 MHz) spectrum of chitonoidoside K_1_ (**4**) in C_5_D_5_N/D_2_O (4/1); Figure S28. The HSQC (500.12 MHz) spectrum of chitonoidoside K_1_ (**4**) in C_5_D_5_N/D_2_O (4/1); Figure S29. The ROESY (500.12 MHz) spectrum of chitonoidoside K_1_ (**4**) in C_5_D_5_N/D_2_O (4/1); Figure S30. The HMBC (500.12 MHz) spectrum of chitonoidoside K_1_ (**4**) in C_5_D_5_N/D_2_O (4/1); Figure S31. 1D TOCSY (500.12 MHz) spectra of Xyl1, Qui2, Glc3, MeGlc4, Glc5 and MeGlc6 of chitonoidoside K_1_ (**4**) in C_5_D_5_N/D_2_O (4/1); Figure S32. HR-ESI-MS and ESI-MS/MS spectra of chitonoidoside K_1_ (**4**); Figure S33. The ^13^C NMR (125.67 MHz) spectrum of chitonoidoside L (**5**) in C_5_D_5_N/D_2_O (4/1); Figure S34. The ^1^H NMR (500.12 MHz) spectrum of chitonoidoside L (**5**) in C_5_D_5_N/D_2_O (4/1); Figure S35. The COSY (500.12 MHz) spectrum of chitonoidoside L (**5**) in C_5_D_5_N/D_2_O (4/1); Figure S36. The HSQC (500.12 MHz) spectrum of chitonoidoside L (**5**) in C_5_D_5_N/D_2_O (4/1); Figure S37. The ROESY (500.12 MHz) spectrum of chitonoidoside L (**5**) in C_5_D_5_N/D_2_O (4/1); Figure S38. The HMBC (500.12 MHz) spectrum of chitonoidoside L (**5**) in C_5_D_5_N/D_2_O (4/1); Figure S39. 1D TOCSY (500.12 MHz) spectra of Xyl1, Qui2, Glc3, MeGlc4, Glc5 and MeGlc6 of chitonoidoside L (**5**) in C_5_D_5_N/D_2_O (4/1); Figure S40. HR-ESI-MS and ESI-MS/MS spectra of chitonoidoside L (**5**); Table S1. ^13^C and ^1^H NMR chemical shifts, HMBC and ROESY correlations of aglycone moiety of chitonoidoside I (**1**); Table S2. ^13^C and ^1^H NMR chemical shifts, HMBC and ROESY correlations of the aglycone part of chitonoidoside J (**2**); Table S3. ^13^C and ^1^H NMR chemical shifts, HMBC and ROESY correlations of the carbohydrate chain of chitonoidoside K_1_ (**4**); Table S4. ^13^C and ^1^H NMR chemical shifts, HMBC and ROESY correlations of the aglycone part of chitonoidoside L (**5**).

## References

[1] Chanley J.D., Ledeen R., Wax J., Nigrelli R.F., Sobotka H. (1959). Holothurin. 1. The isolation, properties and sugar components of holothurin A. J. Am. Chem. Soc..

[2] Stonik V.A., Kalinin V.I., Avilov S.A. (1999). Toxins from the sea cucumbers (Holothuroids): Chemical structures, properties, taxonomic distribution, biosynthesis and evolution. J. Nat. Toxins.

[3] Kalinin V.I., Silchenko A.S., Avilov S.A., Stonik V.A., Smirnov A.V. (2005). Sea cucumbers triterpene glycosides, the recent progress in structural elucidation and chemotaxonomy. Phytochem. Rev..

[4] Mondol M.A.M., Shin H.J., Rahman M.A. (2017). Sea cucmber glycosides: Chemical structures, producing species and important biological properties. Mar. Drugs.

[5] Kalinin V.I., Silchenko A.S., Avilov S.A., Stonik V.A. (2021). Progress in the studies of triterpene glycosides from sea cucumbers (Holothuroidea, Echinodermata) between 2017 and 2021. Nat. Prod. Commun..

[6] Kalinin V.I., Anisimov M.M., Prokofieva N.G., Avilov S.A., Afiyatullov S.S., Stonik V.A., Jangoux M., Lawrence J.M. (1996). Bilological activities and biological role of triterpene glycosides from holothuroids (Echinodermata). Echinoderm Studies.

[7] Chludil H.D., Murray A.P., Seldes A.M., Maier M.S., Rahman A.U. (2003). Biologically active triterpene glycosides from sea cucumbers (Holothurioidea, Echinodermata). Studies in Natural Products Chemistry.

[8] Kalinin V.I., Aminin D.L., Avilov S.A., Silchenko A.S., Stonik V.A., Rahman A.U. (2008). Triterpene glycosides from sea cucucmbers (Holothurioidae, Echinodermata), biological activities and functions. Studies in Natural Product Chemistry (Bioactive Natural Products).

[9] Kim S.-K., Himaya S.W.A. (2012). Triterpene glycosides from sea cucucmbers and their biological activities. Adv. Food Nutr. Res..

[10] Aminin D.L., Menchinskaya E.S., Pislyagin E.A., Silchenko A.S., Avilov S.A., Kalinin V.I., Rahman A.U. (2016). Sea cucumber triterpene glycosides as anticancer agents. Studies in Natural Product Chemistry.

[11] Gomes A.R., Freitas A.C., Duarte A.C., Rocha-Santos T.A.P., Rachman A.U. (2016). Echinoderms: A review of bioactive compounds with potential health effects. Studies in Natural Products Chemistry.

[12] Kalinin V.I., Prokofieva N.G., Likhatskaya G.N., Schentsova E.B., Agafonova I.G., Avilov S.A., Drozdova O.A. (1996). Hemolytic activities of triterpene glycosides from the holothurian order Dendrochirotida: Some trends in the evolution of this group of toxins. Toxicon.

[13] Bryne M., Rowe F., Uthike S. (2010). Molecular, phylogeny and evolution in the family Stichopodidae (Aspidochirotida: Holothurioidea) based on COI and 16S mitochondrial DNA. Mol. Phylogenet. Evol..

[14] Kalinin V.I., Silchenko A.S., Avilov S.A., Stonik V.A. (2019). Non-holostane aglycones of sea cucumber triterpene glycosides. Structure, biosynthesis, evolution. Steroids.

[15] Avilov S.A., Kalinin V.I., Smirnov A.V. (2004). Use of triterpene glycosides for resolving taxonomic problems in the sea cucumber genus *Cucumaria* (Holothorioidea, Echinodermata). Biochem. Syst. Ecol..

[16] Honey-Escandon M., Arreguin-Espinosa R., Solis-Martin F.A., Samyn Y. (2015). Biological and taxonomic perspective of triterpenoid glycosides of sea cucumbers of the family Holothuriidae (Echinodermata, Holothuroidea). Comp. Biochem. Physiol..

[17] Omran N.E., Salem H.K., Eissa S.H., Kabbash A.M., Kandeil ·M.A., Salem M.A. (2020). Chemotaxonomic study of the most abundant Egyptian sea-cucumbers using ultra-performance liquid chromatography (UPLC) coupled to high-resolution mass spectrometry (HRMS). Chemoecology.

[18] Li Y., Wang R., Xun X., Wang J., Bao L., Thimmappa R., Ding J., Jiang J., Zhang L., Li T. (2018). Sea cucumber genome provides insights into saponin biosynthesis and aestivation regulation. Cell Discov..

[19] Claereboudt E.J.S., Gualier G., Decroo C., Colson E., Gerbaux P., Claereboudt M.R., Schaller H., Flammang P., Deleu M., Eeckhaut I. (2019). Triterpenoids in echinoderms: Fundamental differences in diversity and biosynthetic pathways. Mar. Drugs.

[20] Kamyab E., Rohde S., Kellerman M.Y., Schupp P.J. (2020). Chemical defense mechanisms and ecological implications of Indo-Pacific holothurians. Molecules.

[21] Park J.-I., Bae H.-R., Kim C.G., Stonik V.A., Kwak J.Y. (2014). Relationships between chemical structures and functions of triterpene glycosides isolated from sea cucumbers. Front. Chem..

[22] Zelepuga E.A., Silchenko A.S., Avilov S.A., Kalinin V.I. (2021). Structure-activity relationships of holothuroid’s triterpene glycosides and some in silico insights obtained by molecular dynamics study on the mechanisms of their membranolytic action. Mar. Drugs.

[23] Silchenko A.S., Kalinovsky A.I., Avilov S.A., Andrijaschenko P.V., Popov R.S., Dmitrenok P.S., Chingizova E.A., Kalinin V.I. (2021). Unusual structures and cytotoxicities of chitonoidosides A, A_1_, B, C, D, and E, six triterpene glycosides from the Far Eastern sea cucumber *Psolus chitonoides*. Mar. Drugs.

[24] Silchenko A.S., Kalinovsky A.I., Avilov S.A., Andrijaschenko P.V., Popov R.S., Chingizova E.A., Kalinin V.I., Dmitrenok P.S. (2021). Triterpene glycosides from the Far Eastern sea cucumber *Psolus chitonoides*: Chemical structures and cytotoxicities of chitonoidosides E_1_, F, G, and H. Mar. Drugs.

[25] Maltsev I.I., Stonik V.A., Kalinovsky A.I., Elyakov G.B. (1984). Triterpene glycosides from sea cucumber *Stichopus japonicus* Selenka. Comp. Biochem. Physiol..

[26] Silchenko A.S., Avilov S.A., Kalinin V.I., Rahman A.U. (2022). Separation procedures for complicated mixtures of sea cucumber triterpene glycosides with isolation of individual glycosides, their comparison with HPLC/MS metabolomic approach, and biosynthetic interpretation of the obtained structural data. Studies in Natural Product Chemistry.

[27] Silchenko A.S., Kalinovsky A.I., Avilov S.A., Andrijaschenko P.V., Popov R.S., Dmitrenok P.S., Chingizova E.A., Kalinin V.I. (2021). Triterpene glycosides from the Far Eastern sea cucumber *Thyonidium (=Duasmodactyla) kurilensis* (Levin): The structures, cytotoxicities, and biogenesis of kurilosides A_3_, D_1_, G, H, I, I_1_, J, K, and K_1_. Mar. Drugs.

[28] Avilov S.A., Stonik V.A., Kalinovskii A.I. (1990). Structures of four new triterpene glycosides from the holothurian *Cucumaria japonica*. Chem. Nat. Compd..

[29] Drozdova O.A., Avilov S.A., Kalinovskii A.I., Stonik V.A., Mil’grom Y.M., Rashkes Y.V. (1993). Trisulfated glycosides from the holothurian *Cucumaria japonica*. Chem. Nat. Compd..

[30] Silchenko A.S., Kalinovsky A.I., Avilov S.A., Andryjaschenko P.V., Dmitrenok P.S., Kalinin V.I., Yurchenko E.A., Dolmatov I.Y. (2016). Colochirosides A_1_, A_2_, A_3_ and D, four novel sulfated triterpene glycosides from the sea cucumber *Colochirus robustus* (Cucumariidae, Dendrochirotida). Nat. Prod. Commun..

[31] Silchenko A.S., Kalinovsky A.I., Avilov S.A., Kalinin V.I., Andrijaschenko P.V., Dmitrenok P.S., Popov R.S., Chingizova E.A., Ermakova S.P., Malyarenko O.S. (2019). Structures and bioactivities of six new triterpene glycosides, psolusosides E, F, G, H, H_1_ and I and the corrected structure of psolusoside B from the sea cucumber *Psolus fabricii*. Mar. Drugs.

[32] Silchenko A.S., Avilov S.A., Antonov A.S., Kalinovsky A.I., Dmitrenok P.S., Kalinin V.I., Stonik V.A., Woodward C., Collin P.D. (2005). Glycosides from the sea cucumber *Cucumaria frondosa* III. Structure of frondosides A_2_-1, A_2_-2, A_2_-3 and A_2_-6, four new minor monosulfated triterpene glycosides. Can. J. Chem..

[33] Silchenko A.S., Kalinovsky A.I., Avilov S.A., Kalinin V.I., Andrijaschenko P.V., Dmitrenok P.S., Popov R.S., Chingizova E.A. (2019). Structures and bioactivities of psolusosides B_1_, B_2_, J, K, L, M, N, O, P, and Q from the sea cucumber *Psolus fabricii*. The first finding of tetrasulfated marine low molecular weight metabolites. Mar. Drugs.

