# Supplementary material for: Structures and Biologic Activity of Chitonoidosides I, J, K, K1 and L-Triterpene Di-, Tri- and Tetrasulfated Hexaosides from the Sea Cucumber Psolus chitonoides
Source: Mar Drugs. 2022 May 30;20(6):369. doi: 10.3390/md20060369 (PMC9228963; doi:10.3390/md20060369)
Supplement: Supplementary file 1 [file marinedrugs-20-00369-s001.zip › marinedrugs-1746901-supplementary-minor.pdf]

Supplementary data content page

Title: **Structures and Biologic Activity of Chitonoidosides I, J, K, K_1 and L – Triterpene Di-, Tri- and Tetrasulfated Hexaosides from the Sea Cucumber *Psolus chitonoides***

Authors: Alexandra S. Silchenko, Sergey A. Avilov, Pelageya V. Andrijaschenko, Roman S. Popov, Ekaterina A. Chingizova, Pavel S. Dmitrenok, Anatoly I. Kalinovsky, Anton И. Rasin, Vladimir I. Kalinin*

Address: [1]*G.B. Elyakov Pacific Institute of Bioorganic Chemistry, Far Eastern Branch of Russian Academy of Sciences, Pr. 100-let Vladivostoka 159, 690022 Vladivostok, Russia*

Correspondence kalininv@piboc.dvo.ru; Tel.: +7-423-231-1168

Content:

Figure S1. The ^{13}C NMR (125.67 MHz) spectrum of chitonoidoside I (**1**) in C_5D_5N/D_2O (4/1)

Figure S2. The ^1H NMR (500.12 MHz) spectrum of chitonoidoside I (**1**) in C_5D_5N/D_2O (4/1)

Figure S3. The COSY (500.12 MHz) spectrum of chitonoidoside I (**1**) in C_5D_5N/D_2O (4/1)

Figure S4. The HSQC (500.12 MHz) spectrum of chitonoidoside I (**1**) in C_5D_5N/D_2O (4/1)

Figure S5. The ROESY (500.12 MHz) spectrum of chitonoidoside I (**1**) in C_5D_5N/D_2O (4/1)

Figure S6. The HMBC (500.12 MHz) spectrum of chitonoidoside I (**1**) in C_5D_5N/D_2O (4/1)

Figure S7. 1 D TOCSY (500.12 MHz) spectra of Xyl1, Qui2, Xyl3, Glc4, Glc5 and MeGlc6 of chitonoidoside I (**1**) in C_5D_5N/D_2O (4/1)

Figure S8. HR-ESI-MS and ESI-MS/MS spectra of chitonoidoside I (**1**)

Table S1. ^{13}C and ^1H NMR chemical shifts, HMBC and ROESY correlations of aglycone moiety of chitonoidoside I (**1**)

Figure S9. The ^{13}C NMR (125.67 MHz) spectrum of chitonoidoside J (**2**) in C_5D_5N/D_2O (4/1)

Figure S10. The ^1H NMR (500.12 MHz) spectrum of chitonoidoside J (**2**) in C_5D_5N/D_2O (4/1)

Figure S11. The COSY (500.12 MHz) spectrum of chitonoidoside J (**2**) in C_5D_5N/D_2O (4/1)

Figure S12. The HSQC (500.12 MHz) spectrum of chitonoidoside J (**2**) in C_5D_5N/D_2O (4/1)

Figure S13. The HMBC (500.12 MHz) spectrum of chitonoidoside J (**2**) in C_5D_5N/D_2O (4/1)

Figure S14. The ROESY (500.12 MHz) spectrum of chitonoidoside J (**2**) in C_5D_5N/D_2O (4/1)

Figure S15. 1D TOCSY (500.12 MHz) spectra of Xyl1, Qui2, Xyl3, MeGlc4, Glc5, MeGlc6 of chitonoidoside J (**2**) in C_5D_5N/D_2O (4/1).

Figure S16. HR-ESI-MS and ESI-MS/MS spectra of chitonoidoside J (**2**)

Table S2. ^{13}C and ^{1}H NMR chemical shifts, HMBC and ROESY correlations of the aglycone part of chitonoidoside J (**2**)

Figure S17. The ^{13}C NMR (125.67 MHz) spectrum of chitonoidoside K (**3**) in C_5D_5N/D_2O (4/1)

Figure S18. The ^{1}H NMR (500.12 MHz) spectrum of chitonoidoside K (**3**) in C_5D_5N/D_2O (4/1)

Figure S19. The COSY (500.12 MHz) spectrum of chitonoidoside K (**3**) in C_5D_5N/D_2O (4/1)

Figure S20. The HSQC (500.12 MHz) spectrum of chitonoidoside K (**3**) in C_5D_5N/D_2O (4/1)

Figure S21. The HMBC (500.12 MHz) spectrum of chitonoidoside K (**3**) in C_5D_5N/D_2O (4/1)

Figure S22. The ROESY (500.12 MHz) spectrum of chitonoidoside K (**3**) in C_5D_5N/D_2O (4/1)

Figure S23. 1 D TOCSY (500.12 MHz) spectra of Xyl1, Qui2, Glc3, MeGlc4, Glc5, MeGlc6 of chitonoidoside K (**3**) in C_5D_5N/D_2O (4/1)

Figure S24. HR-ESI-MS and ESI-MS/MS spectra of chitonoidoside K (**3**)

Figure S25. The ^{13}C NMR (125.67 MHz) spectrum of chitonoidoside K_1 (**4**) in C_5D_5N/D_2O (4/1)

Figure S26. The ^{1}H NMR (500.12 MHz) spectrum of chitonoidoside K_1 (**4**) in C_5D_5N/D_2O (4/1)

Figure S27. The COSY (500.12 MHz) spectrum of chitonoidoside K_1 (**4**) in C_5D_5N/D_2O (4/1)

Figure S28. The HSQC (500.12 MHz) spectrum of chitonoidoside K_1 (**4**) in C_5D_5N/D_2O (4/1)

Figure S29. The ROESY (500.12 MHz) spectrum of chitonoidoside K_1 (**4**) in C_5D_5N/D_2O (4/1)

Figure S30. The HMBC (500.12 MHz) spectrum of chitonoidoside K_1 (**4**) in C_5D_5N/D_2O (4/1)

Figure S31. 1D TOCSY (500.12 MHz) spectra of Xyl1, Qui2, Glc3, MeGlc4, Glc5 and MeGlc6 of chitonoidoside K_1 (**4**) in C_5D_5N/D_2O (4/1)

Figure S32. HR-ESI-MS and ESI-MS/MS spectra of chitonoidoside K_1 (**4**)

Table S3. ^{13}C and ^{1}H NMR chemical shifts, HMBC and ROESY correlations of the carbohydrate chain of chitonoidoside K_1 (**4**)

Figure S33. The ^{13}C NMR (125.67 MHz) spectrum of chitonoidoside L (**5**) in C_5D_5N/D_2O (4/1)

Figure S34. The ^{1}H NMR (500.12 MHz) spectrum of chitonoidoside L (**5**) in C_5D_5N/D_2O (4/1)

Figure S35. The COSY (500.12 MHz) spectrum of chitonoidoside L (**5**) in C_5D_5N/D_2O (4/1)

Figure S36. The HSQC (500.12 MHz) spectrum of chitonoidoside L (**5**) in C_5D_5N/D_2O (4/1)

Figure S37. The ROESY (500.12 MHz) spectrum of chitonoidoside L (**5**) in C_5D_5N/D_2O (4/1)

Figure S38. The HMBC (500.12 MHz) spectrum of chitonoidoside L (**5**) in C_5D_5N/D_2O (4/1)

Figure S39. 1D TOCSY (500.12 MHz) spectra of Xyl1, Qui2, Glc3, MeGlc4, Glc5 and MeGlc6 of chitonoidoside L (**5**) in C_5D_5N/D_2O (4/1)

Figure S40. HR-ESI-MS and ESI-MS/MS spectra of chitonoidoside L (**5**)

Table S4. ^{13}C and ^{1}H NMR chemical shifts, HMBC and ROESY correlations of the aglycone part of chitonoidoside L (**5**)

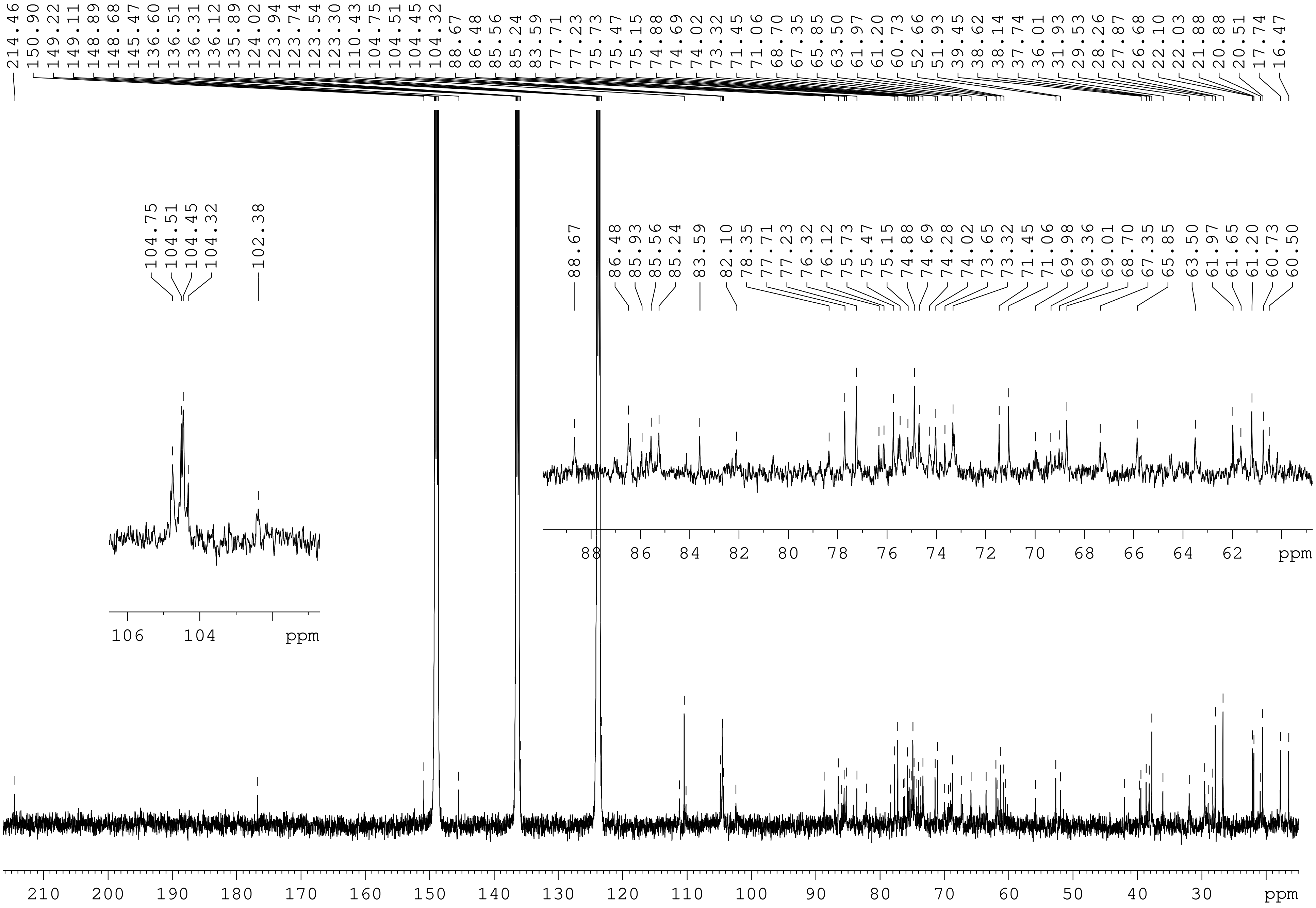

Figure S1. The ^{13}C NMR (125.67 MHz) spectrum of chitonoidoside I (**1**) in C_5D_5N/D_2O (4/1)

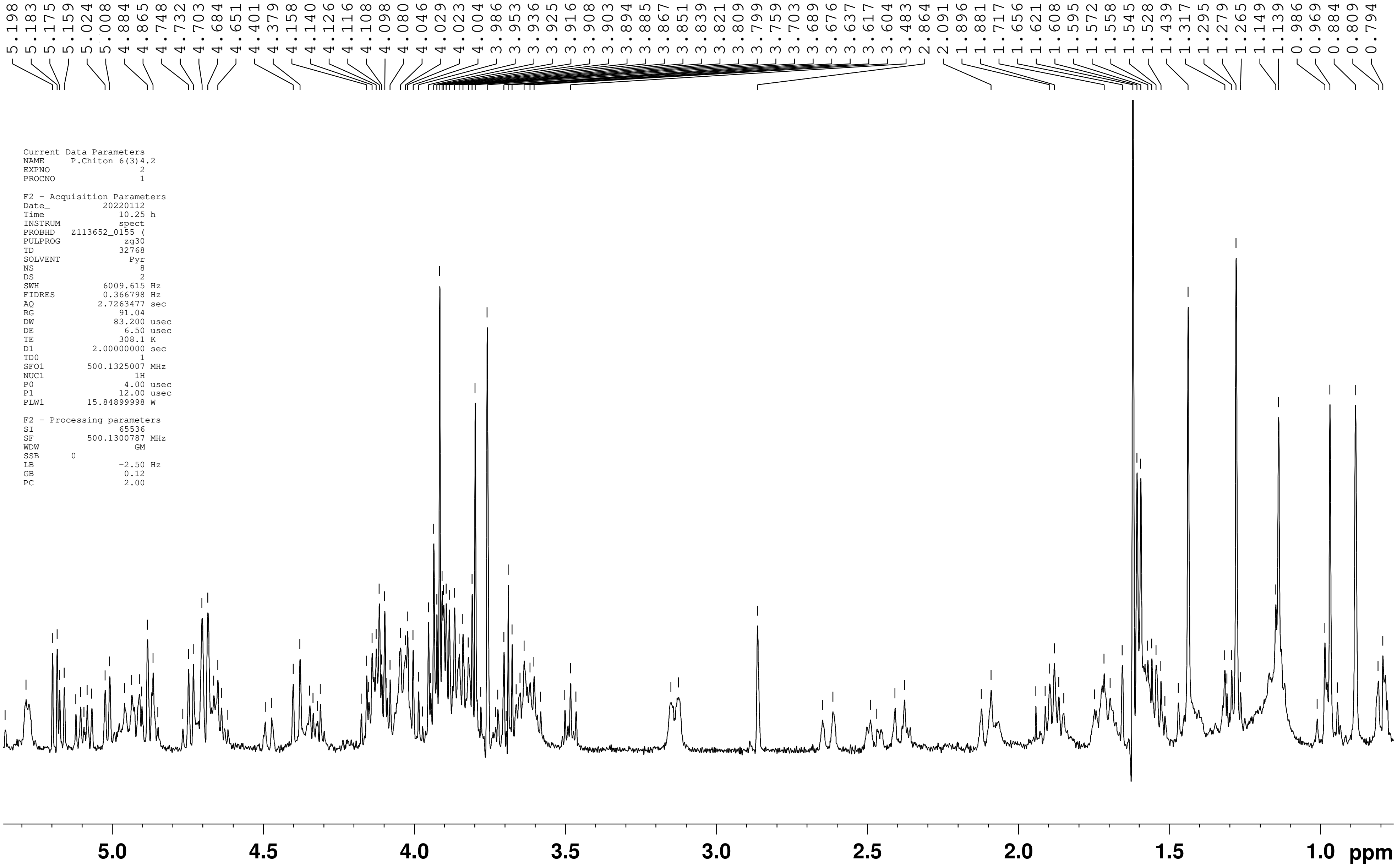

Figure S2. The ^{1}H NMR (500.12 MHz) spectrum of chitonoidoside I (**1**) in C_5D_5N/D_2O (4/1)

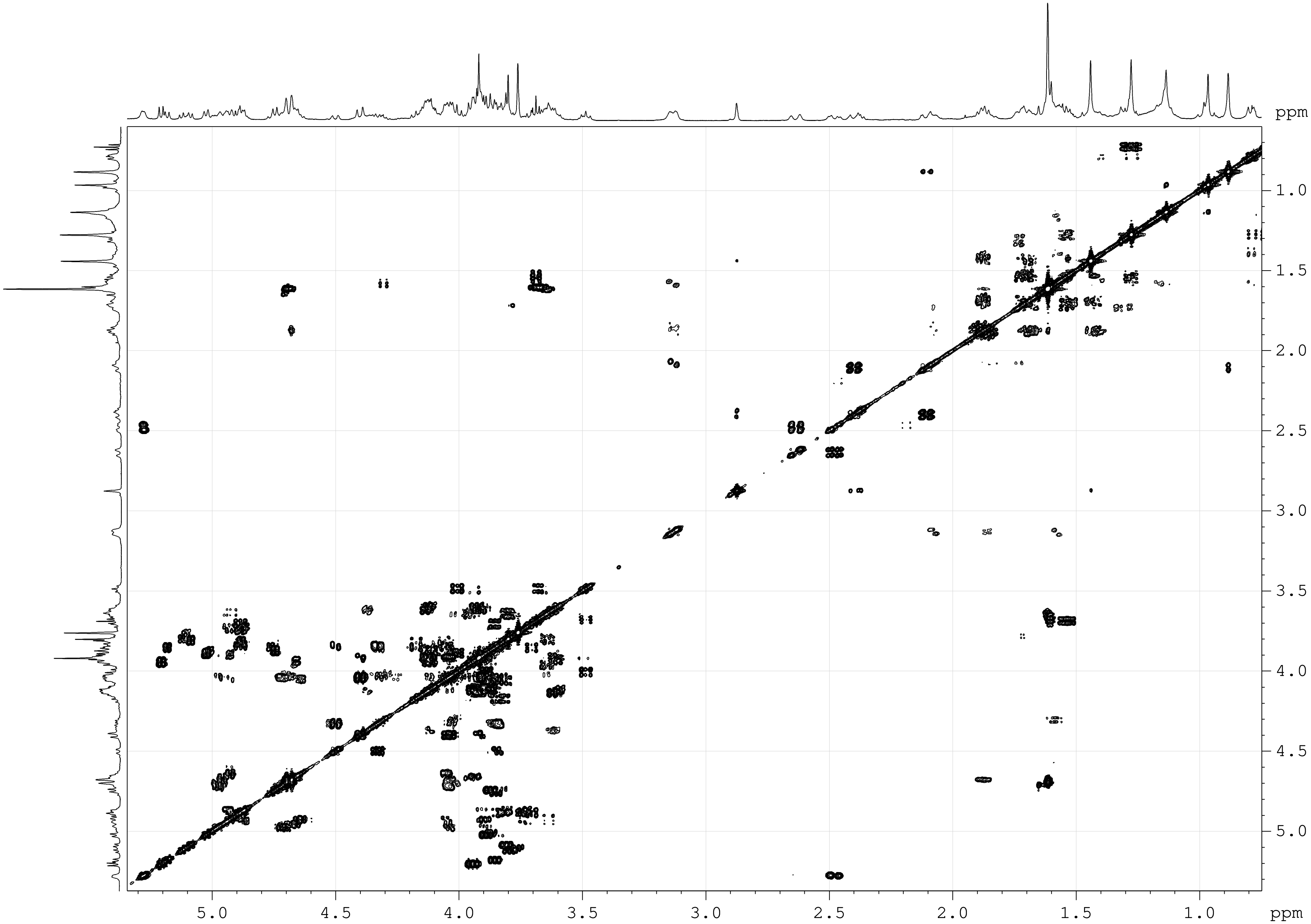

Figure S3. The COSY (500.12 MHz) spectrum of chitonoidoside I (**1**) in C_5D_5N/D_2O (4/1)

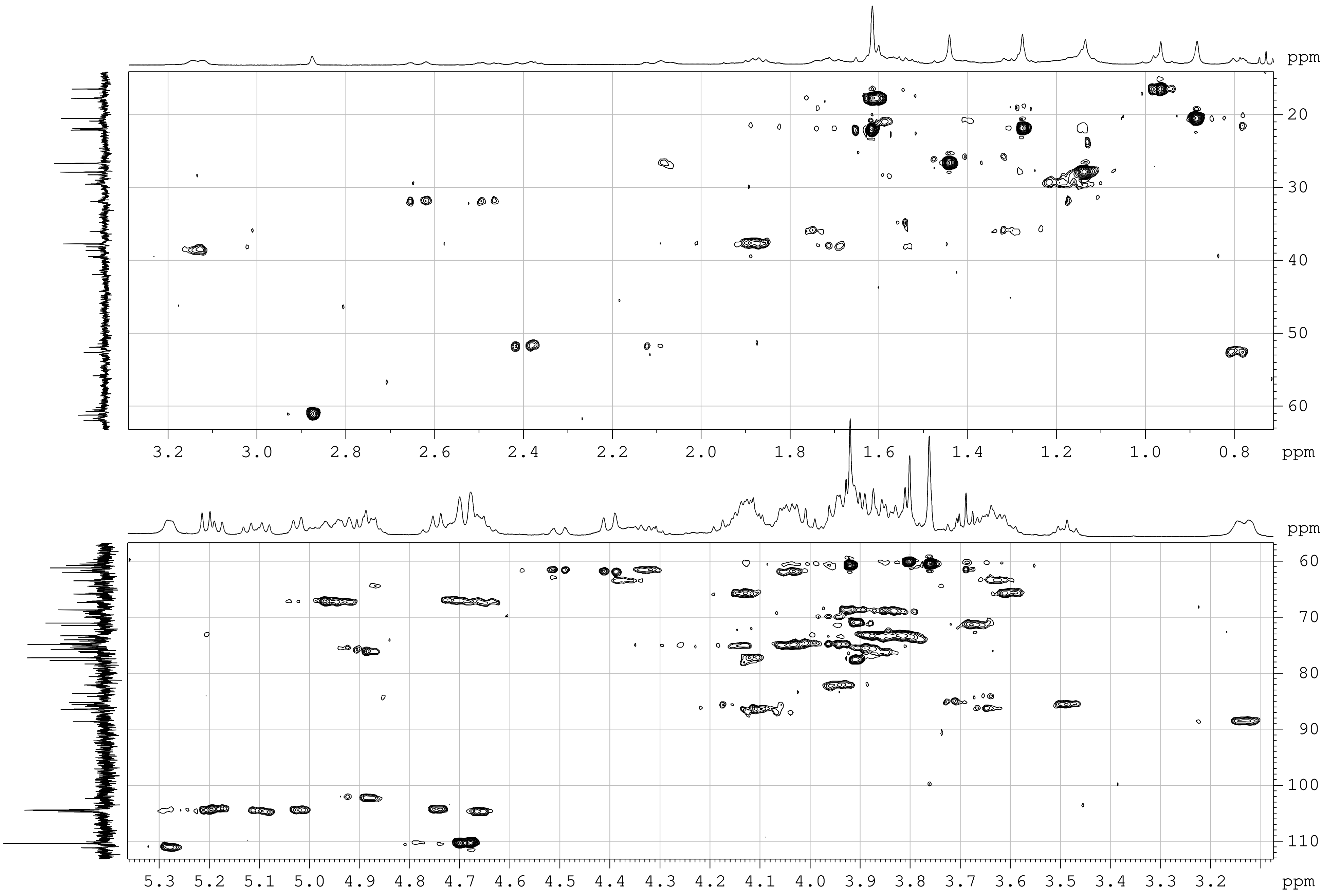

Figure S4. The HSQC (500.12 MHz) spectrum of chitonoidoside I (**1**) in C_5D_5N/D_2O (4/1)

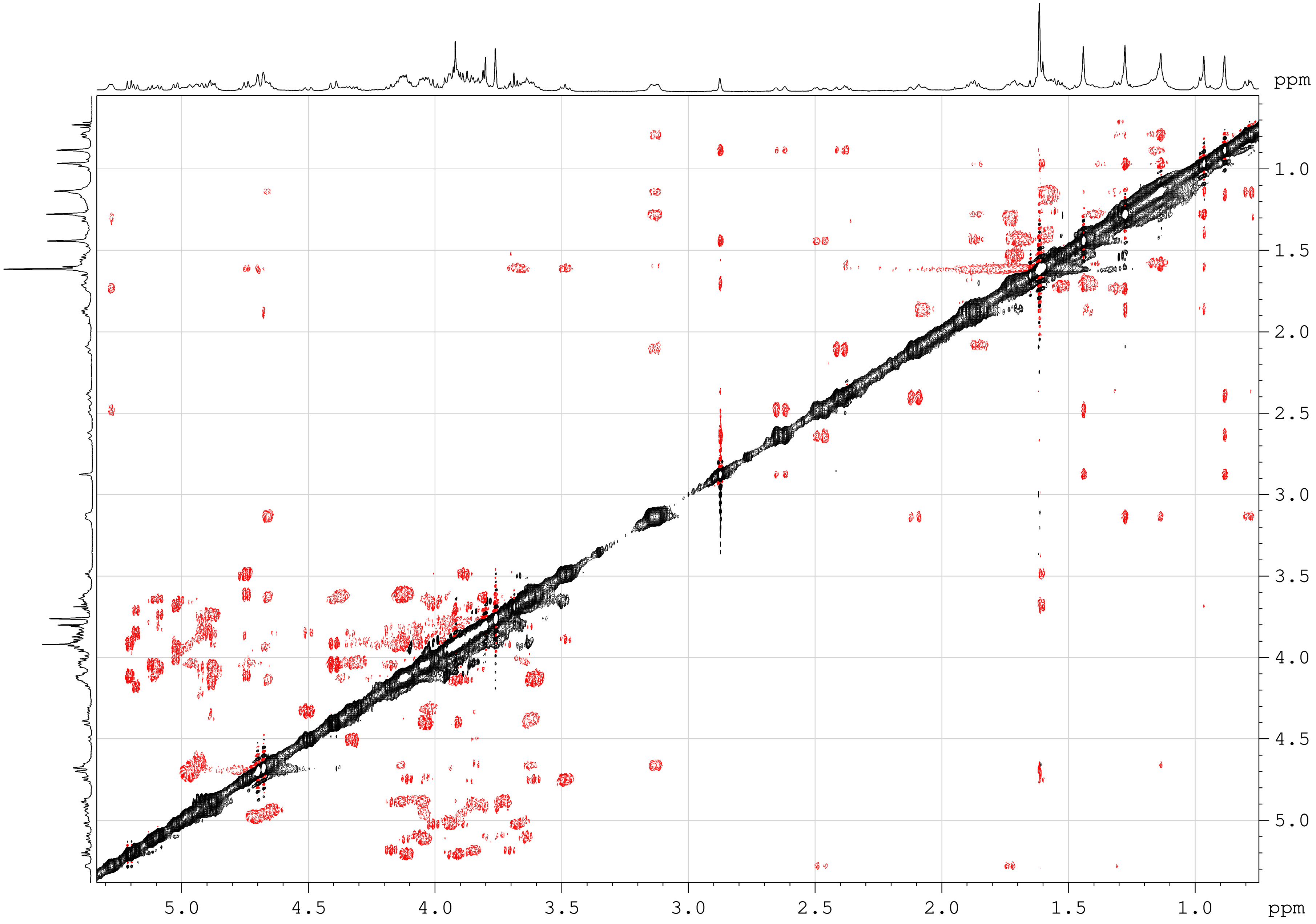

Figure S5. The ROESY (500.12 MHz) spectrum of chitonoidoside I (**1**) in C_5D_5N/D_2O (4/1)

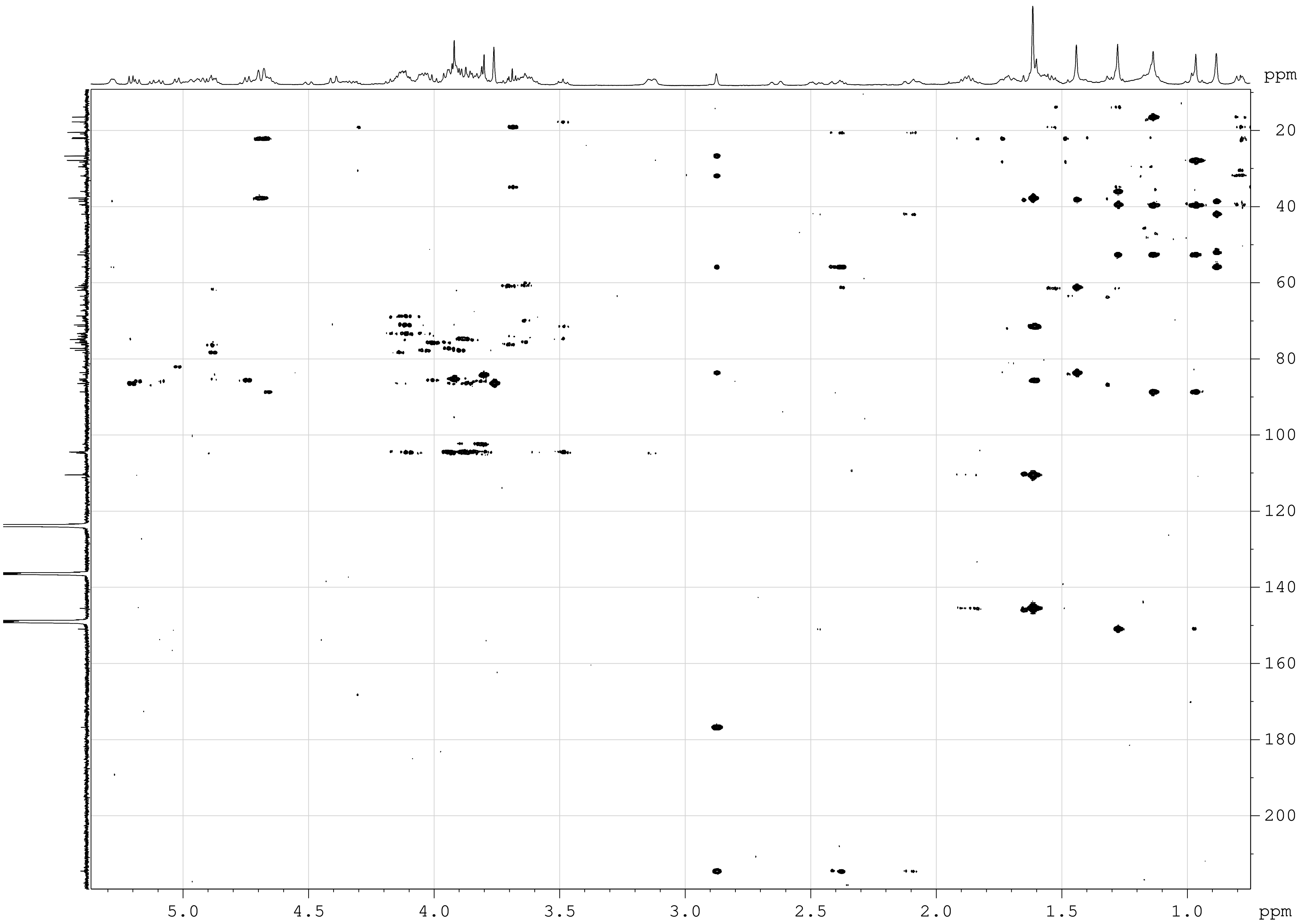

Figure S6. The HMBC (500.12 MHz) spectrum of chitonoidoside I (**1**) in C_5D_5N/D_2O (4/1)

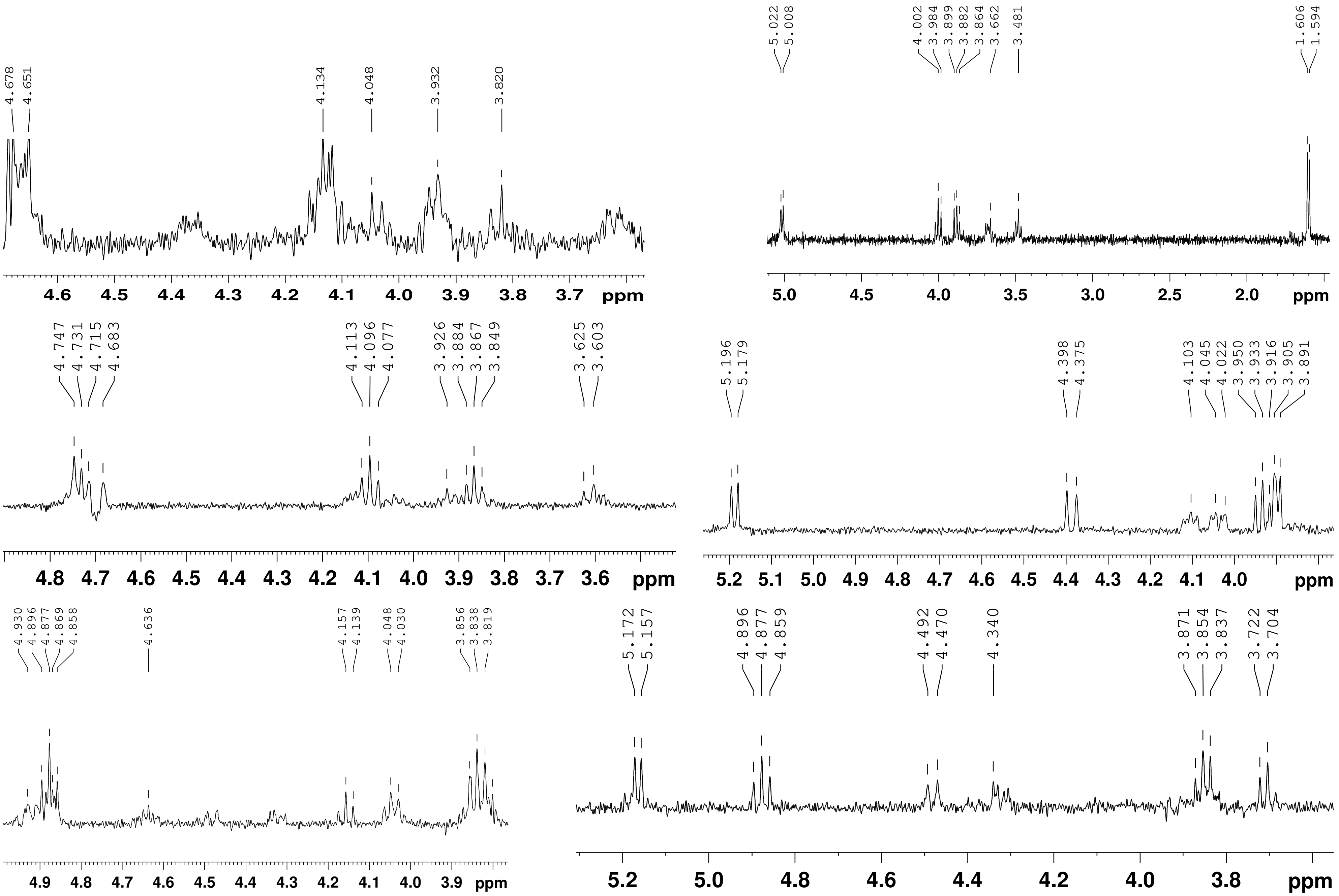

Figure S7. 1 D TOCSY (500.12 MHz) spectra of Xyl1, Qui2, Xyl3, Glc4, Glc5 and MeGlc6 of chitonoidoside I (**1**) in C_5D_5N/D_2O (4/1)

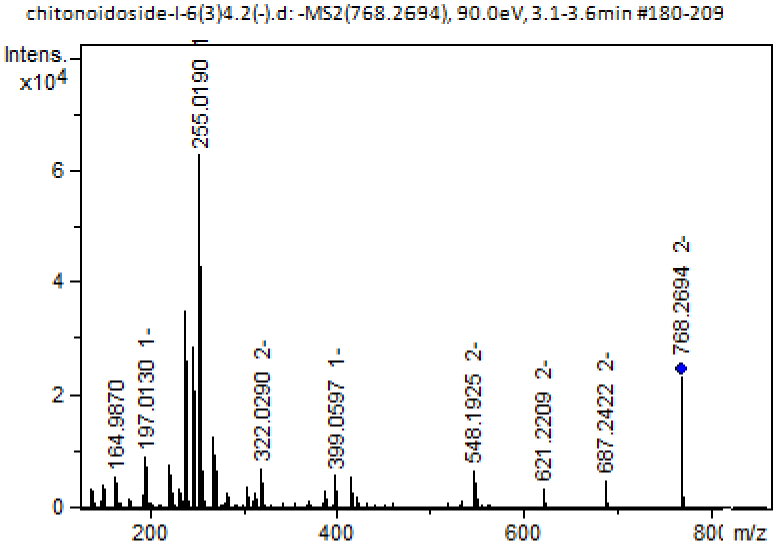

Figure S8. HR-ESI-MS (–) and ESI-MS/MS (–) spectra of chitonoidoside I (**1**)

Table S1. ^{13}C and ^{1}H NMR chemical shifts and HMBC and ROESY correlations of aglycone moiety of chitonoidoside I (**1**).

Position	δ_C mult.[a]	δ_H mult. (*J* in Hz)[b]	HMBC	ROESY
1	36.0 CH_2	1.75 m		H-11, H-19
		1.31 m		
2	26.7 CH_2	2.08 m		
		1.87 m		H-19
3	88.7 CH	3.13 dd (4.0; 11.2)	C: 1 Xyl1	H-5, H-31, H1-Xyl1
4	39.5 C			
5	52.7 CH	0.79 brd (11.6)	C: 10, 19, 30	H-3, H-31
6	20.9 CH_2	1.58 m		
		1.40 m		H-19
7	28.3 CH_2	1.58 m		
		1.17 m		H-32
8	38.6 CH	3.14 m		H-19
9	150.9 C			
10	39.6 C			
11	111.1 CH	5.28 m	C: 10, 13	H-1, H-19
12	31.9 CH_2	2.64 d (17.4)	C: 18	H-17, H-32
		2.48 dd (5.8; 17.4)	C: 11, 14	H-21
13	55.8 C			
14	41.9 C			
15	51.9 CH_2	2.40 d (15.8)	C: 13, 16, 32	H-7, H-32
		2.11 d (15.8)	C: 14, 16, 32	H-8
16	214.5 C			
17	61.2 CH	2.88 s	C: 12, 13, 16, 18, 20, 21	H-21, H-32
18	176.7 C			
19	21.9 CH_3	1.28 s	C: 1, 5, 9, 10	H-1, H-2, H-8, H-30
20	83.6 C			
21	26.7 CH_3	1.44 s	C: 17, 20, 22	H-12, H-17
22	38.1 CH_2	1.71 m	C: 20, 21, 23	H-21
		1.54 m	C: 17, 20, 21, 23	
23	22.0 CH_2	1.71 m		
		1.45 m		
24	37.7 CH_2	1.88 m	C: 25, 26, 27	H-21
25	145.5 C			
26	110.4 CH_2	4.70 brs	C: 24, 27	
		4.68 brs	C: 24, 27	
27	22.1 CH_3	1.62 s	C: 24, 25, 26	
30	16.5 CH_3	0.98 s	C: 3, 4, 5, 31	H-2, H-6, H-31,
31	27.9 CH_3	1.15 s	C: 3, 4, 5, 30	H-3, H-5, H-6, H-30
32	20.5 CH_3	0.88 s	C: 8, 13, 14, 15	H-7, H-12, H-15, H-17

[a] Recorded at 125.67 MHz in C_5D_5N. [b] Recorded at 500.12 MHz in C_5D_5N.

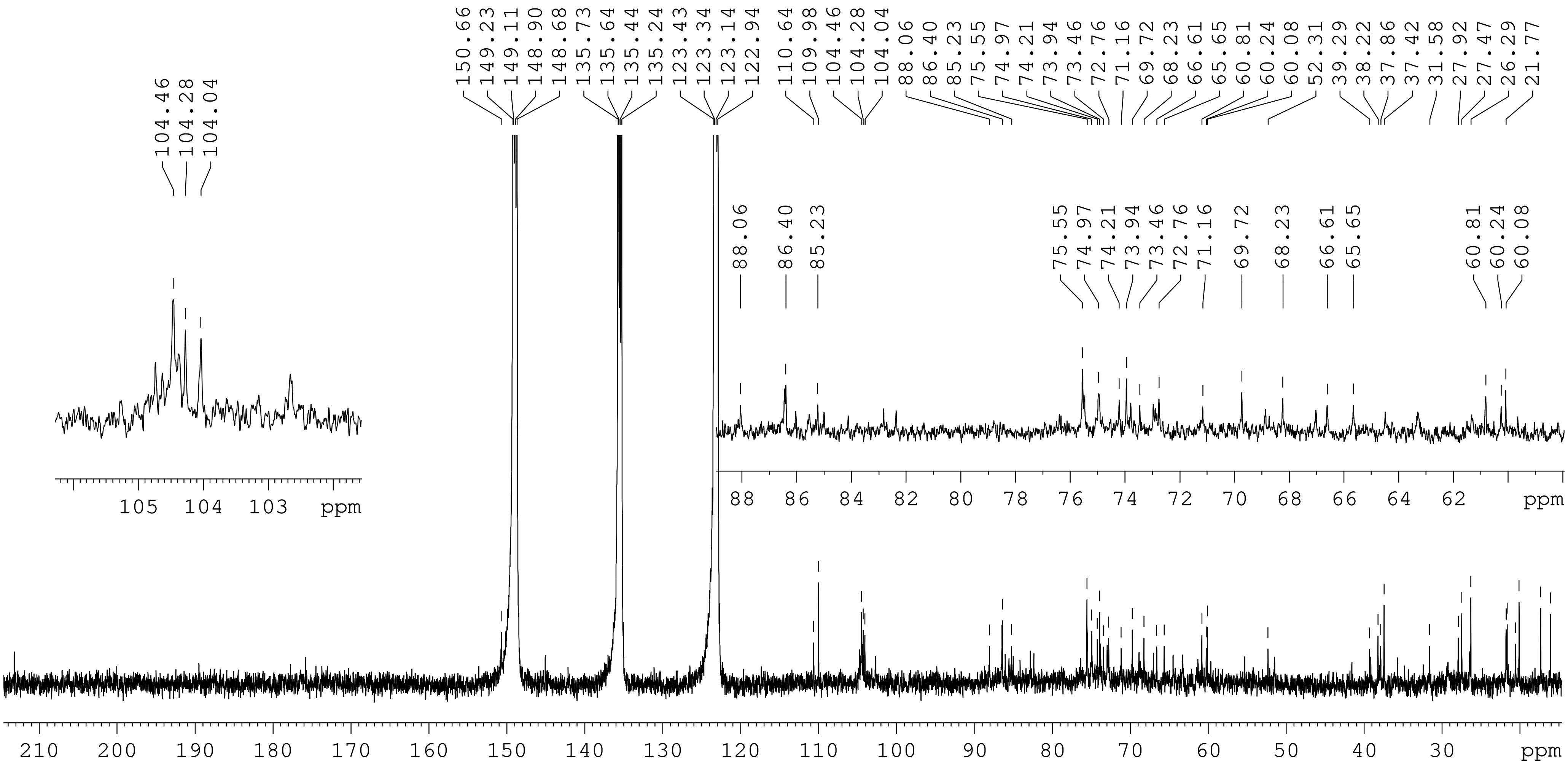

Figure S9. The ^{13}C NMR (125.67 MHz) spectrum of chitonoidoside J (**2**) in C_5D_5N/D_2O (4/1)

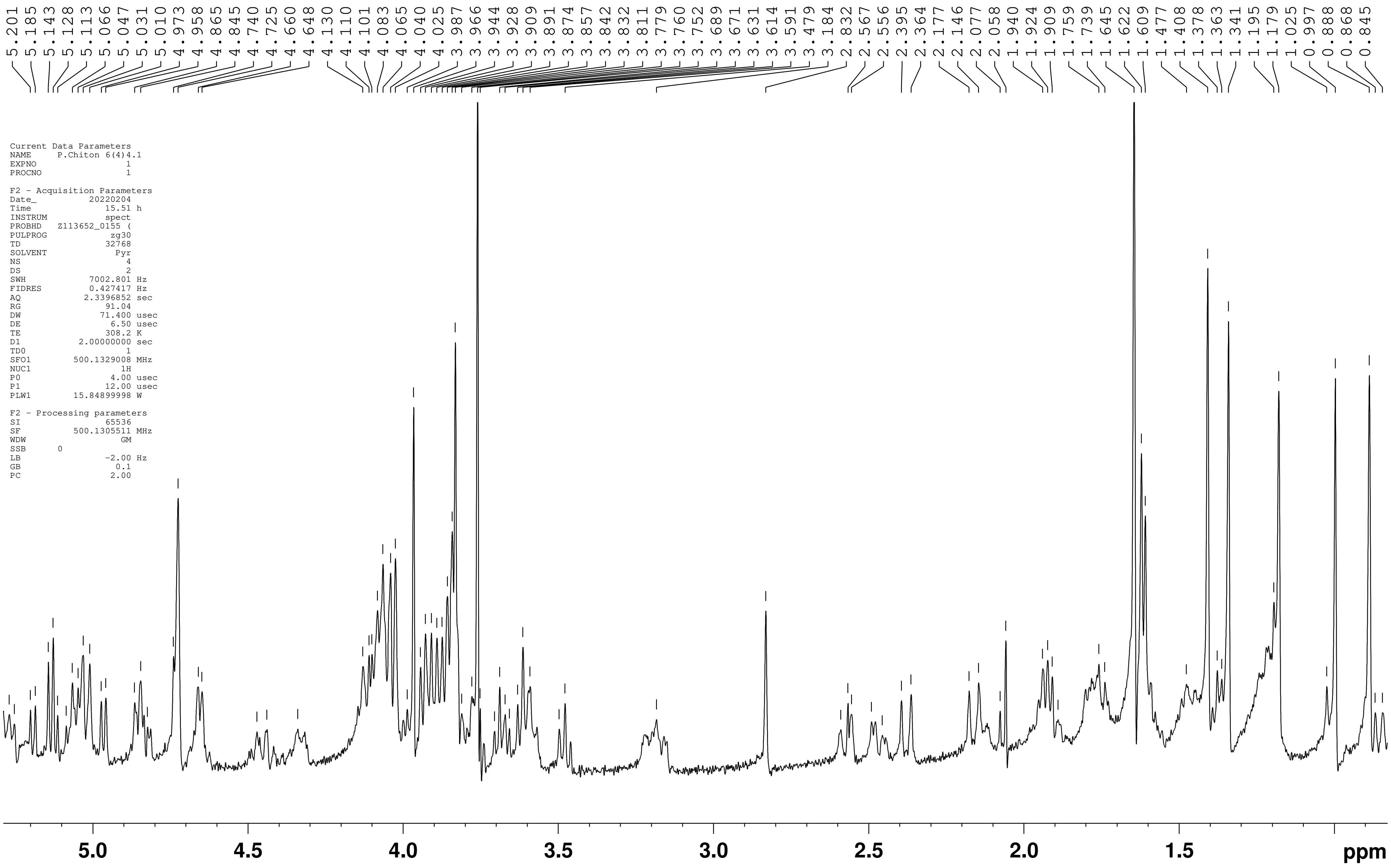

Figure S10. The ^{1}H NMR (500.12 MHz) spectrum of chitonoidoside J (**2**) in C_5D_5N/D_2O (4/1)

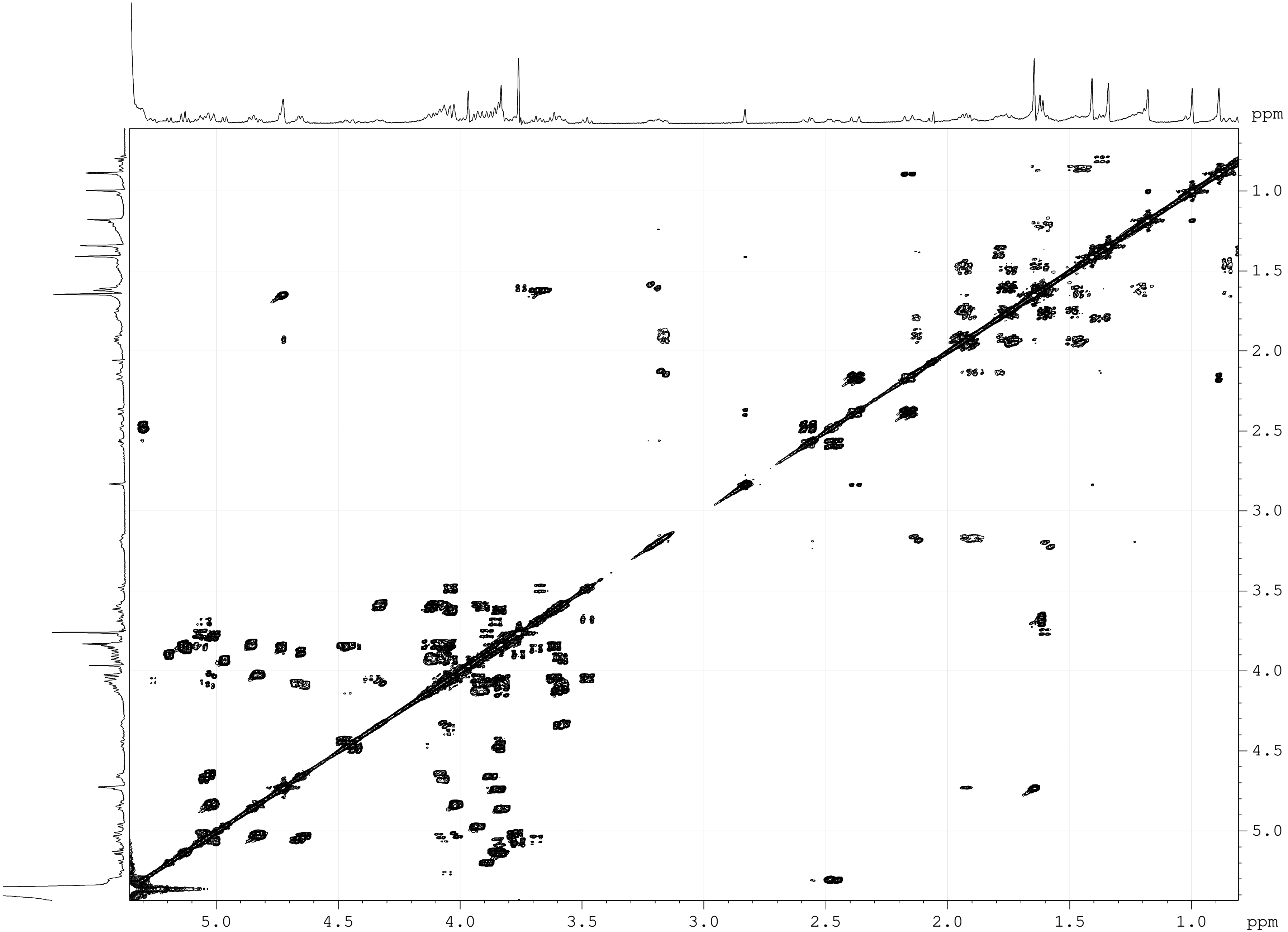

Figure S11. The COSY (500.12 MHz) spectrum of chitonoidoside J (**2**) in C_5D_5N/D_2O (4/1)

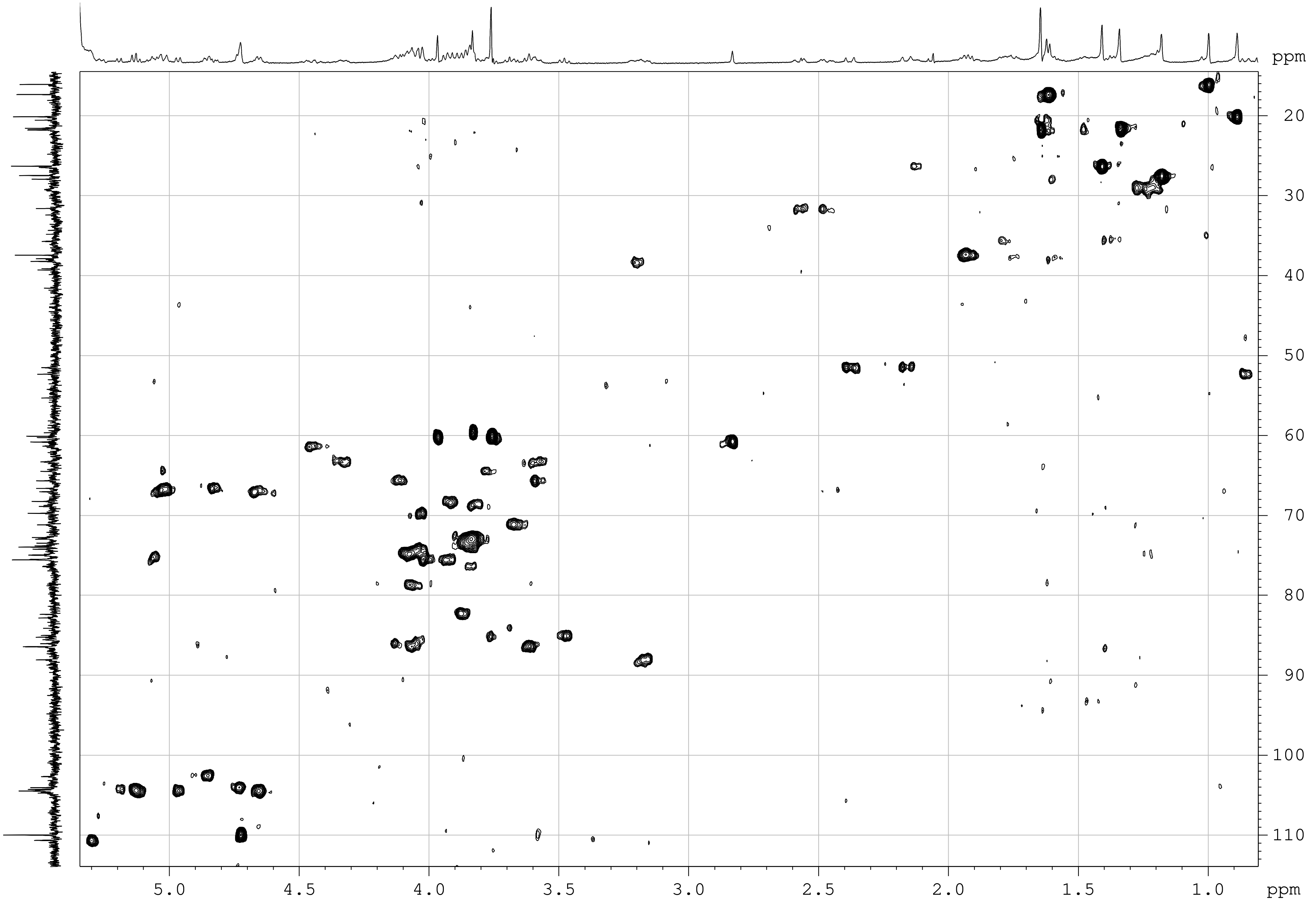

Figure S12. The HSQC (500.12 MHz) spectrum of chitonoidoside J (**2**) in C_5D_5N/D_2O (4/1)

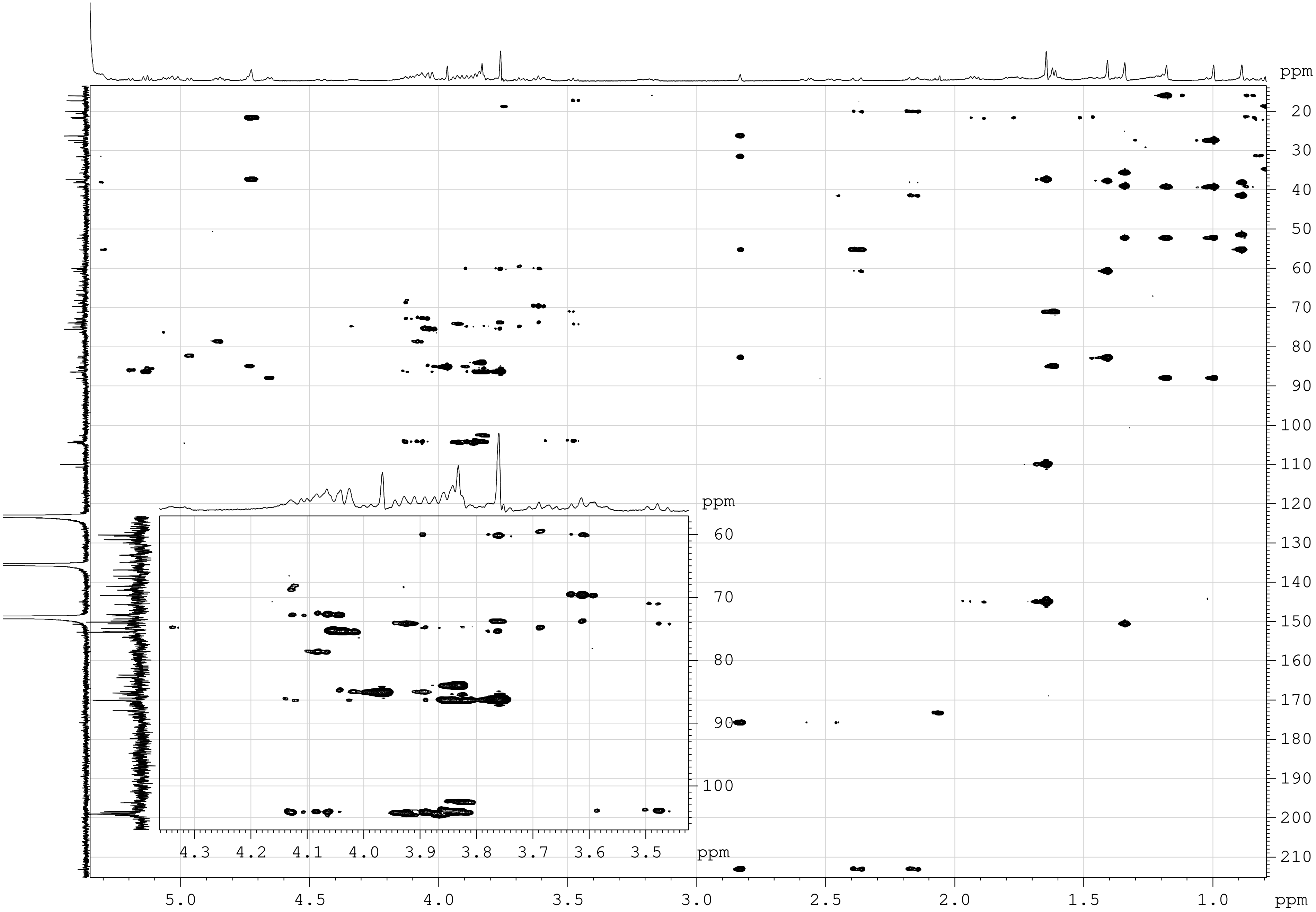

Figure S13. The HMBC (500.12 MHz) spectrum of chitonoidoside J (**2**) in C_5D_5N/D_2O (4/1)

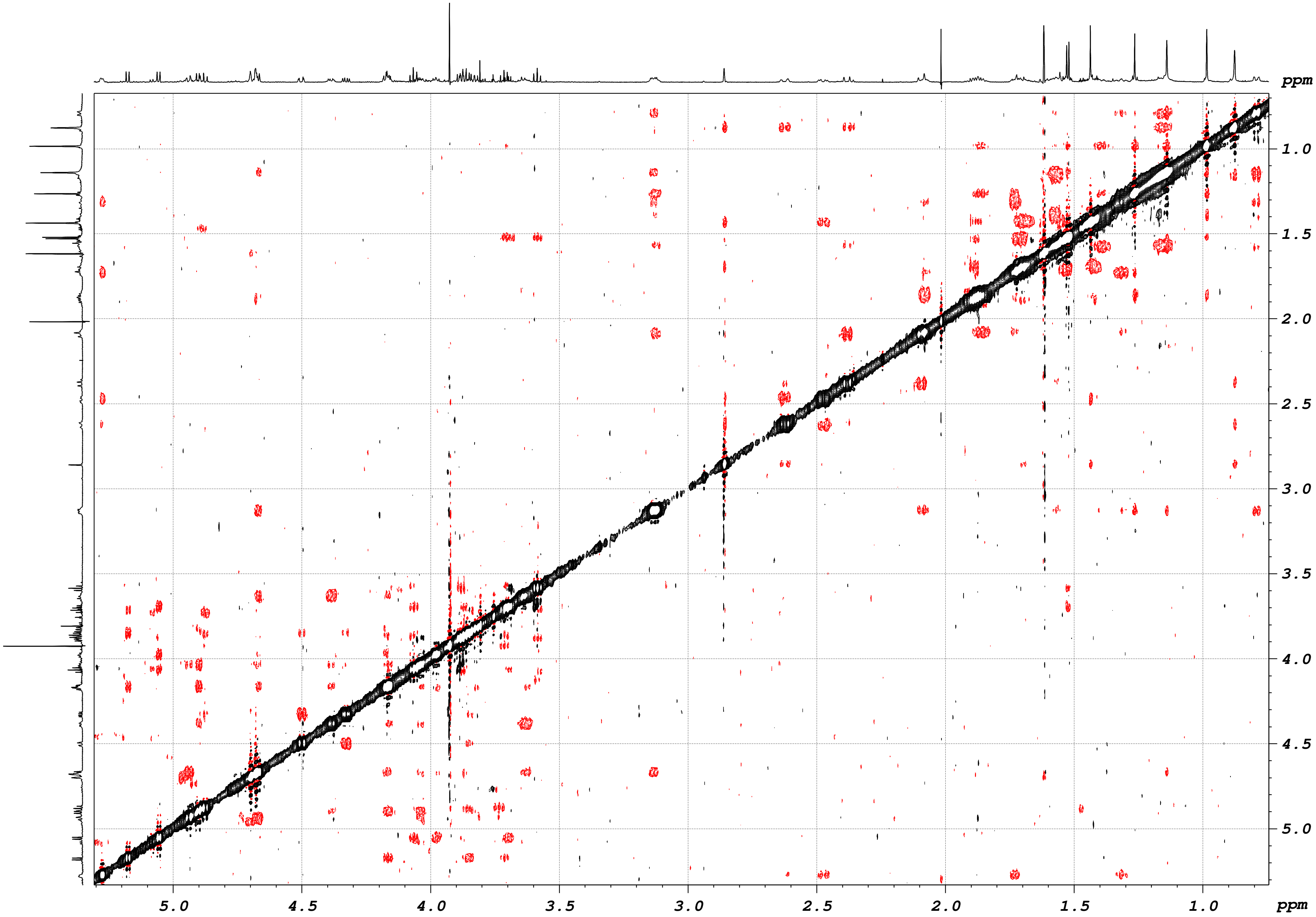

Figure S14. The ROESY (500.12 MHz) spectrum of chitonoidoside J (**2**) in C_5D_5N/D_2O (4/1)

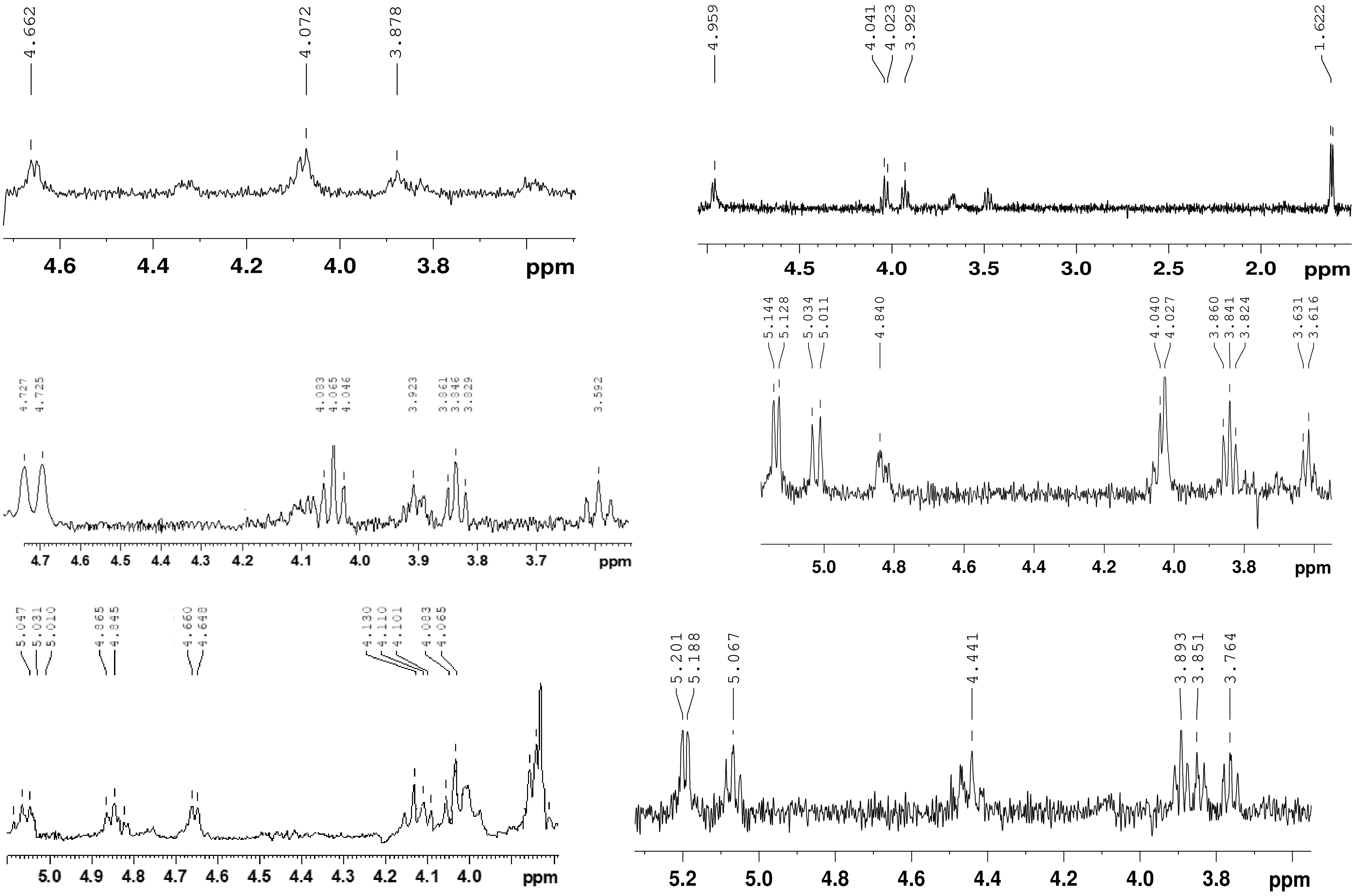

Figure S15. 1D TOCSY (500.12 MHz) spectra of Xyl1, Qui2, Xyl3, MeGlc4, Glc5, MeGlc6 of chitonoidoside J (**2**) in C_5D_5N/D_2O (4/1).

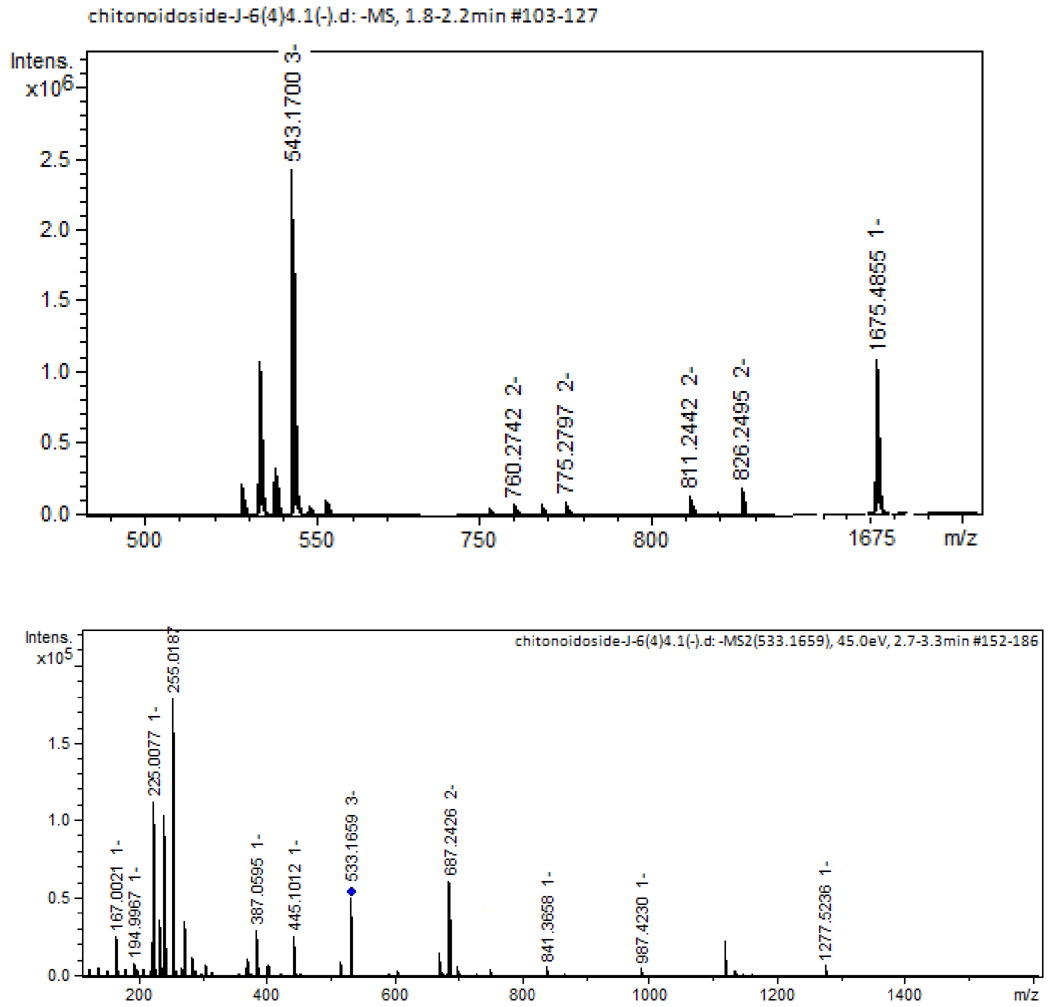

Figure S16. HR-ESI-MS and ESI-MS/MS spectra of chitonoidoside J (**2**)

Table S2. ^{13}C and ^{1}H NMR chemical shifts, HMBC and ROESY correlations of the aglycone moiety of chitonoidoside J (**2**).

Position	δ_C mult. [a]	δ_H mult. (*J* in Hz) [b]	HMBC	ROESY
1	35.7 CH_2	1.79 m		H-11
		1.37 m		
2	26.5 CH_2	2.13 m		
3	88.1 CH	3.17 dd (4.2; 12.0)	C: 30	H-5, H-31, H1-Xyl1
4	39.1 C			
5	52.3 CH	0.86 brd (11.2)	C: 19, 30	H-3, H-31
6	20.5 CH_2	1.62 m		
		1.46 m		
7	27.9 CH_2	1.60 m		
		1.21 m		H-32
8	38.2 CH	3.19 m		H-15, H-19
9	150.7 C			
10	39.3 C			
11	110.6 CH	5.30 m	C: 10, 13	H-1
12	31.6 CH_2	2.57 brd (16.7)	C: 18	H-17, H-32
		2.47 dd (5.8; 16.7)	C: 13, 18	H-21
13	55.3 C			
14	41.5 C			
15	51.5 CH_2	2.37 d (15.6)	C: 13, 16, 32	H-32
		2.16 d (15.6)	C: 14, 16, 32	H-8
16	213.2 C			
17	60.8 CH	2.83 s	C: 12, 16, 18, 20, 21	H-12, H-21, H-22, H-32
18	176.0 C			
19	21.5 CH_3	1.34 s	C: 1, 5, 9, 10	H-1, H-8, H-11, H-30
20	82.8 C			
21	26.3 CH_3	1.41 s	C: 17, 20, 22	H-12, H-17, H-22
22	37.9 CH_2	1.74 m		
		1.59 m		
23	21.8 CH_2	1.61 m		
		1.48 m		
24	37.4 CH_2	1.93 m		
25	145.1 C			
26	110.0 CH_2	4.73 brs	C: 24, 27	
27	21.7 CH_3	1.65 s	C: 25, 26	H-26
30	16.1 CH_3	1.00 s	C: 3, 4, 5, 31	
31	27.5 CH_3	1.18 s	C: 3, 4, 5, 30	H-5, H-30
32	20.1 CH_3	0.89 s	C: 8, 13, 14, 15	H-17

[a]Recorded at 125.67 MHz in C_5D_5N/D_2O (4/1). [b]Recorded at 500.12 MHz in C_5D_5N/D_2O (4/1).

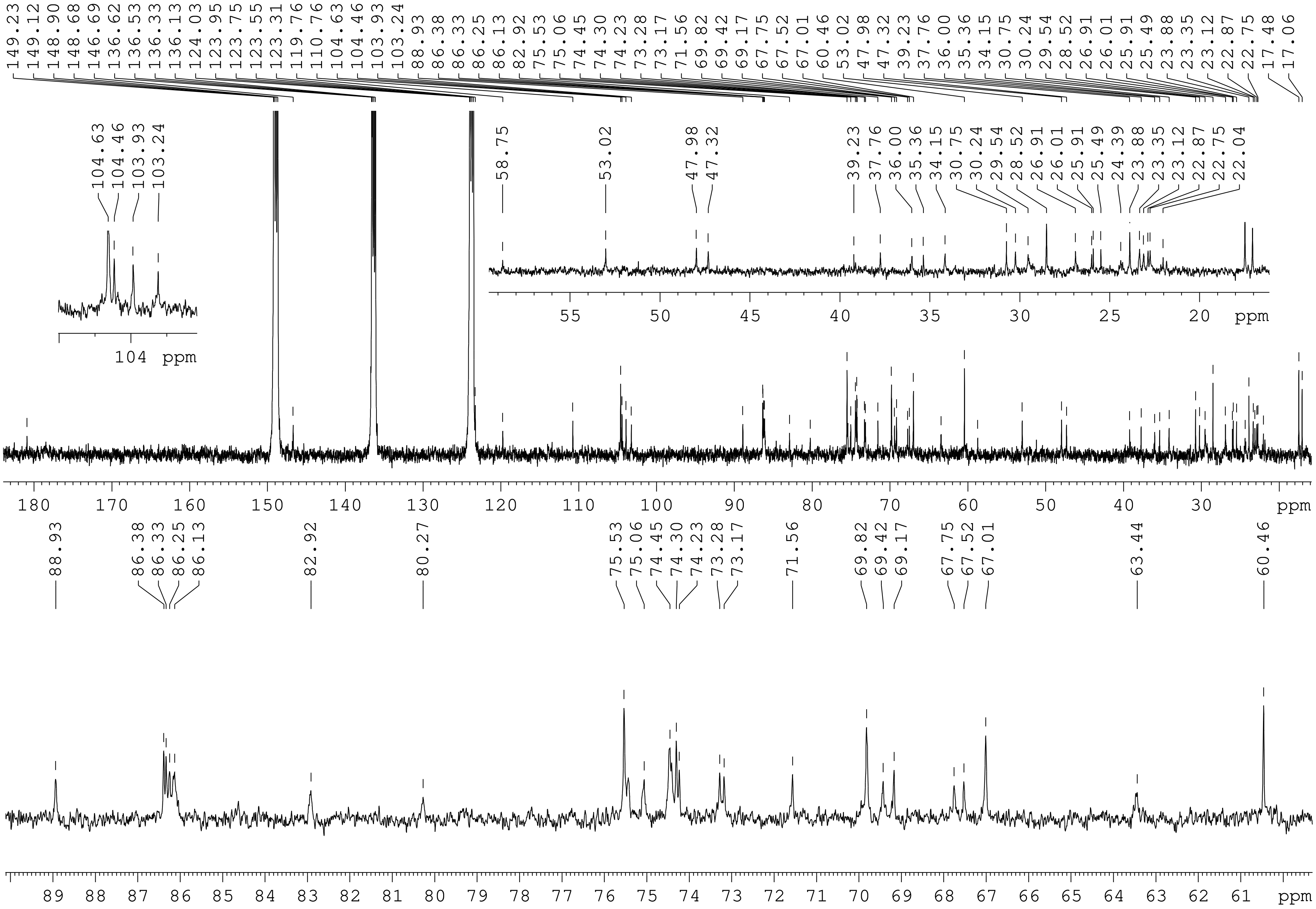

Figure S17. The ^{13}C NMR (125.67 MHz) spectrum of chitonoidoside K (**3**) in C_5D_5N/D_2O (4/1)

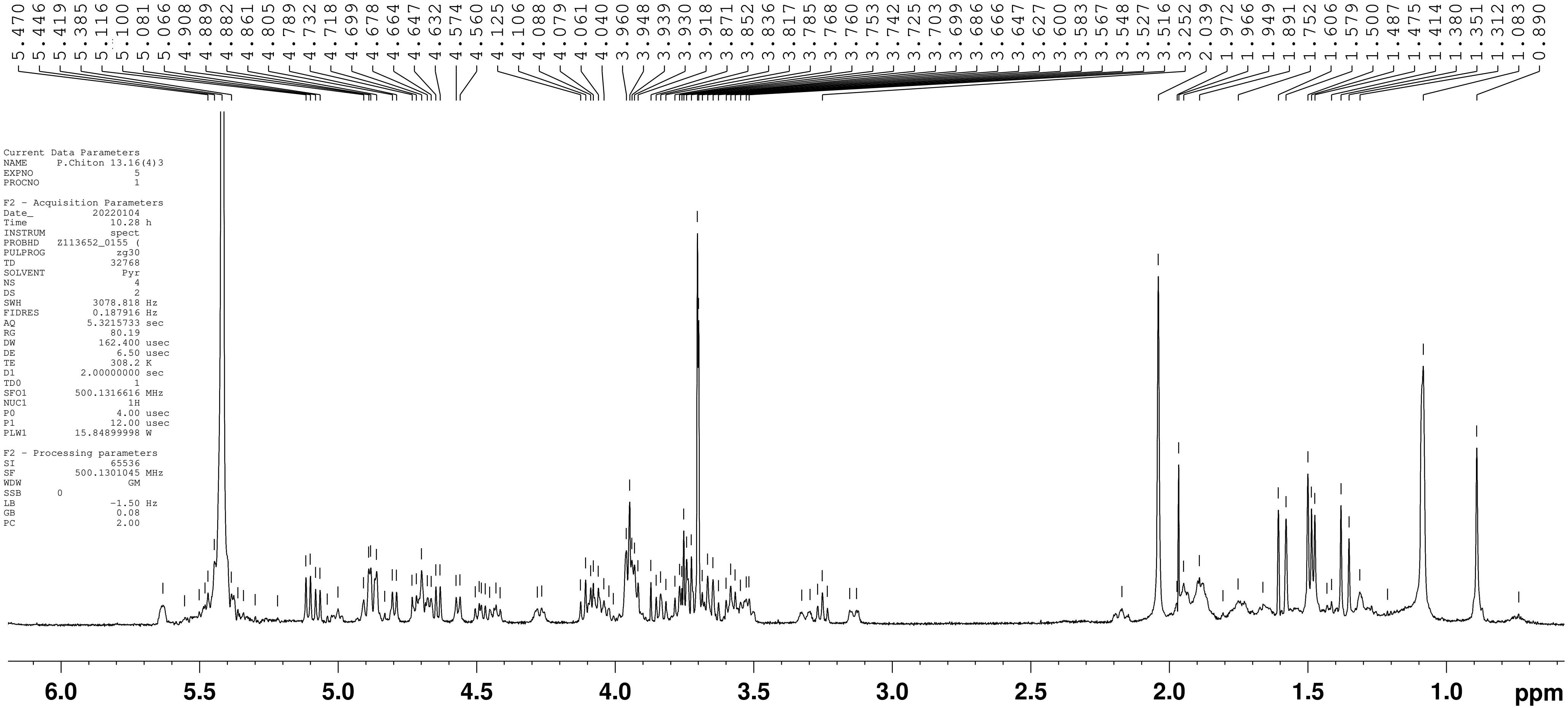

Figure S18. The ^{1}H NMR (500.12 MHz) spectrum of chitonoidoside K (**3**) in C_5D_5N/D_2O (4/1)

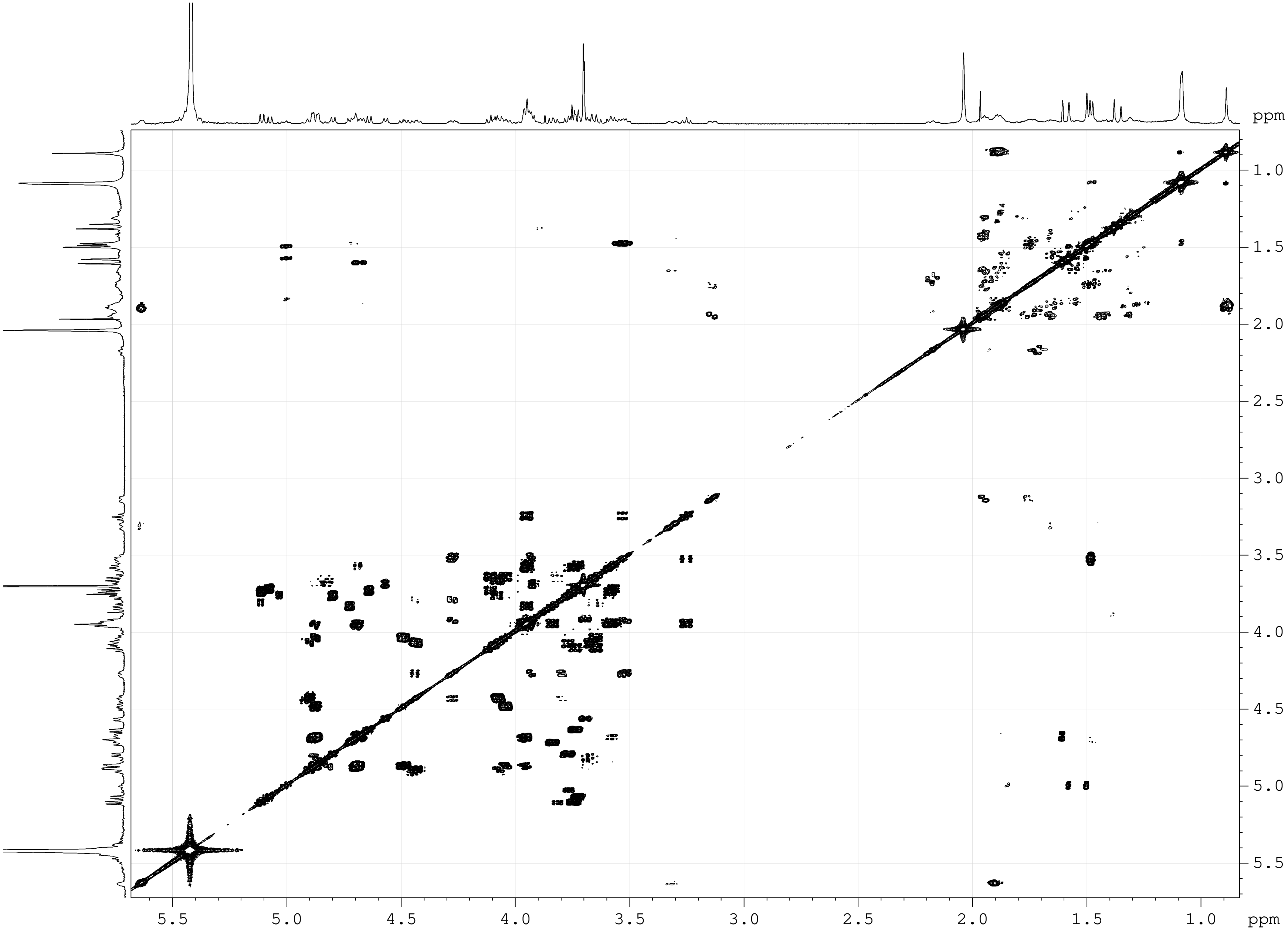

Figure S19. The COSY (500.12 MHz) spectrum of chitonoidoside K (**3**) in C_5D_5N/D_2O (4/1)

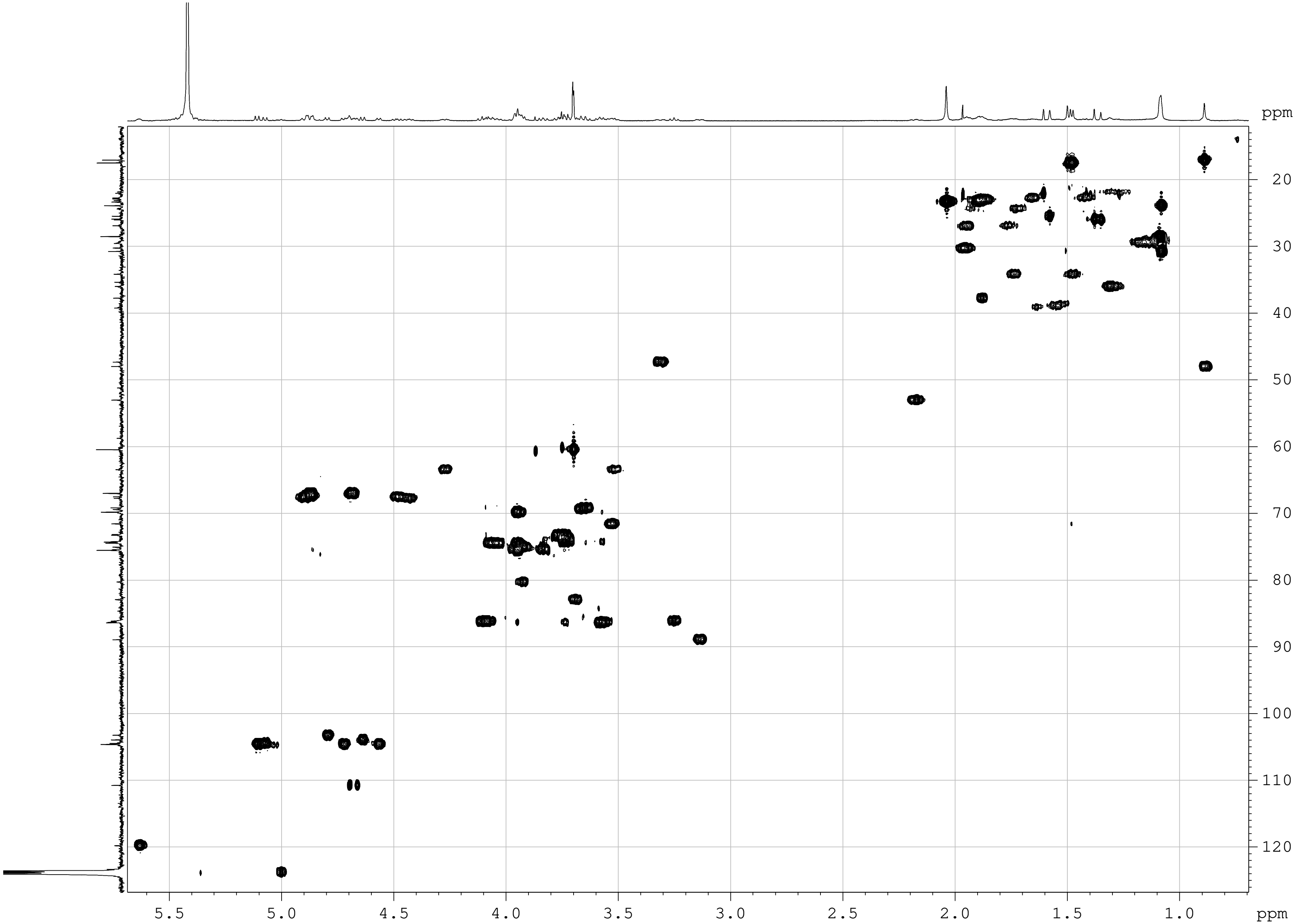

Figure S20. The HSQC (500.12 MHz) spectrum of chitonoidoside K (**3**) in C_5D_5N/D_2O (4/1)

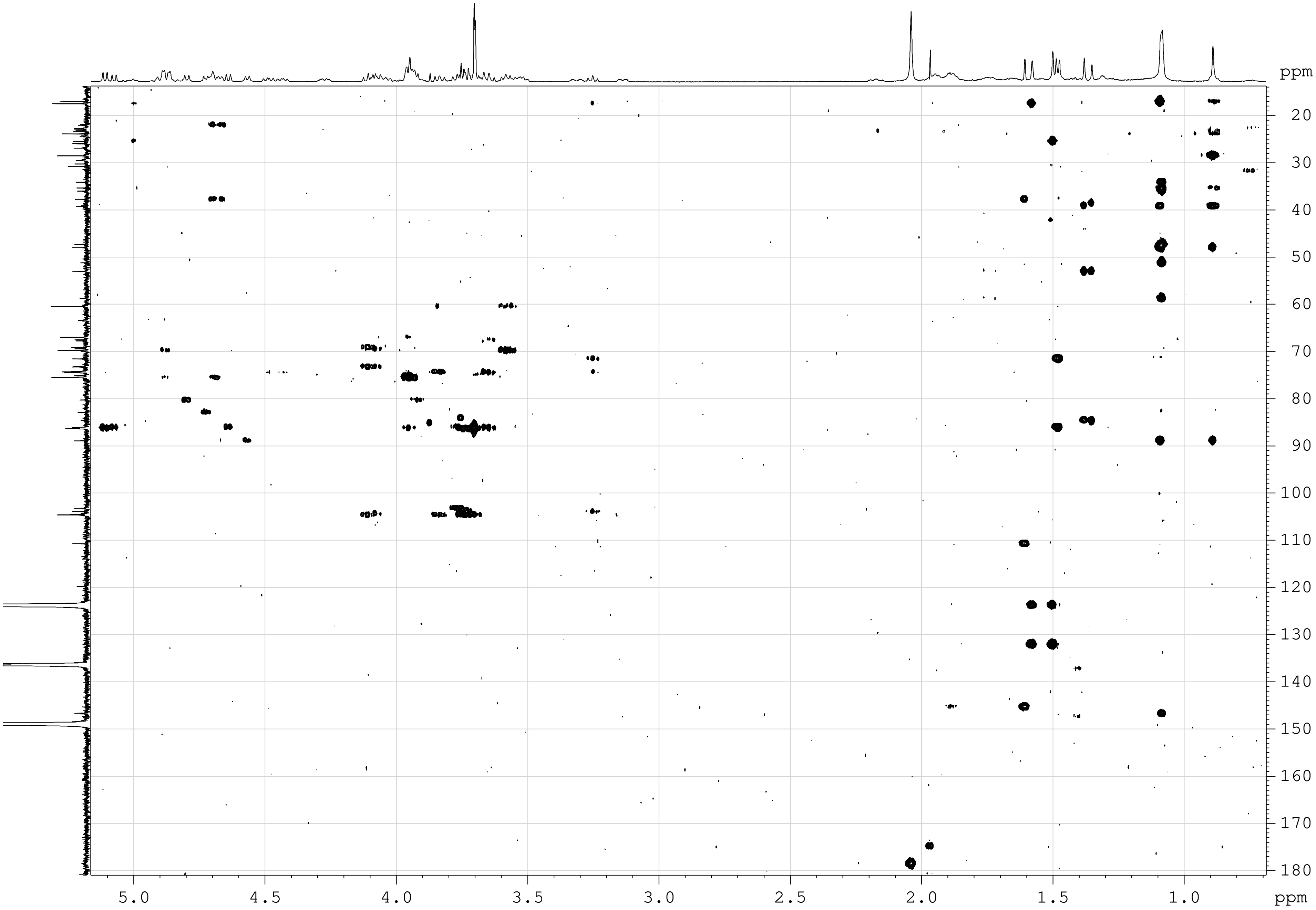

Figure S21. The HMBC (500.12 MHz) spectrum of chitonoidoside K (**3**) in C_5D_5N/D_2O (4/1)

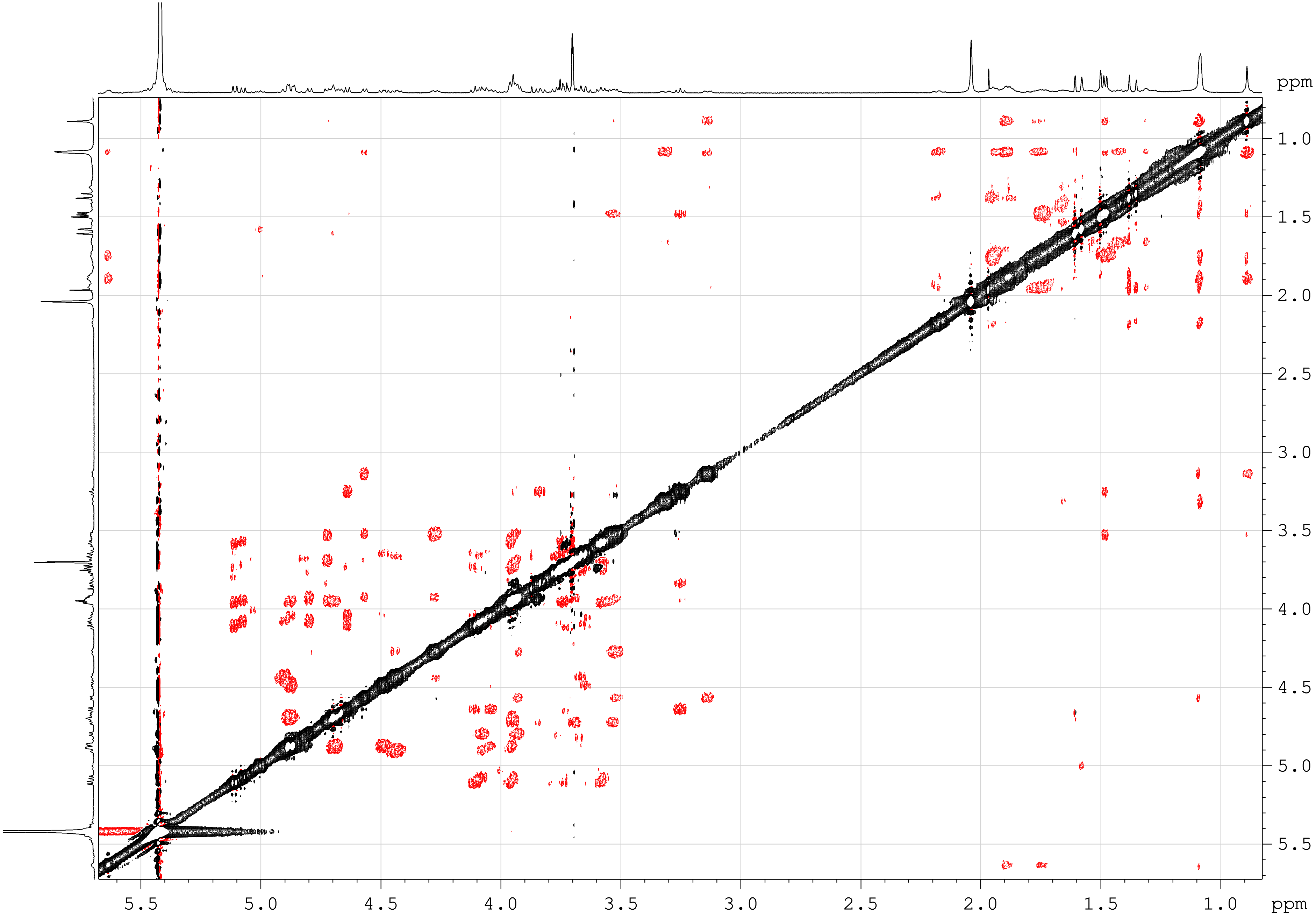

Figure S22. The ROESY (500.12 MHz) spectrum of chitonoidoside K (**3**) in C_5D_5N/D_2O (4/1)

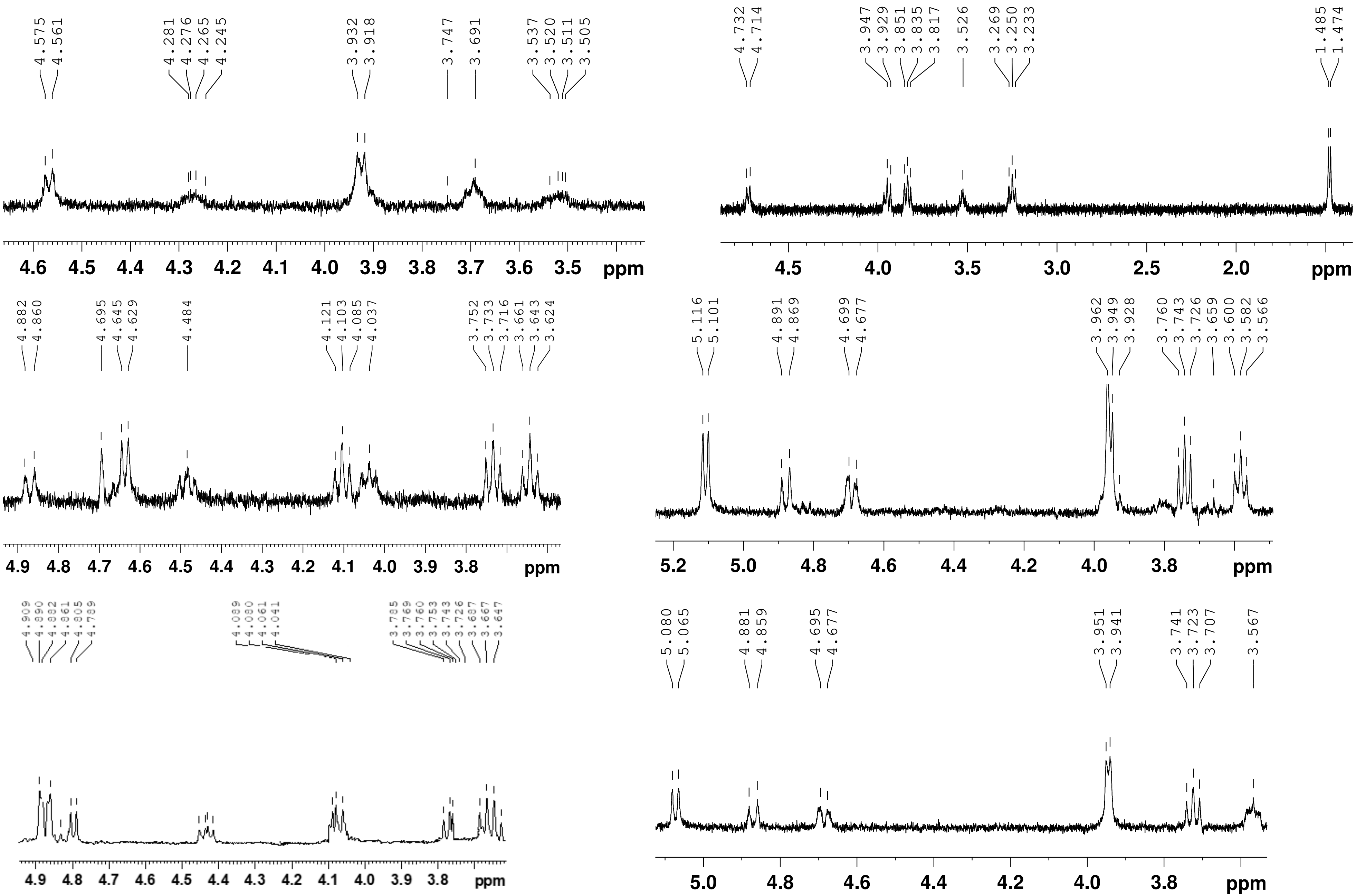

Figure S23. 1 D TOCSY (500.12 MHz) spectra of Xyl1, Qui2, Glc3, MeGlc4, Glc5 and MeGlc6 of chitonoidoside K (**3**) in C_5D_5N/D_2O (4/1)

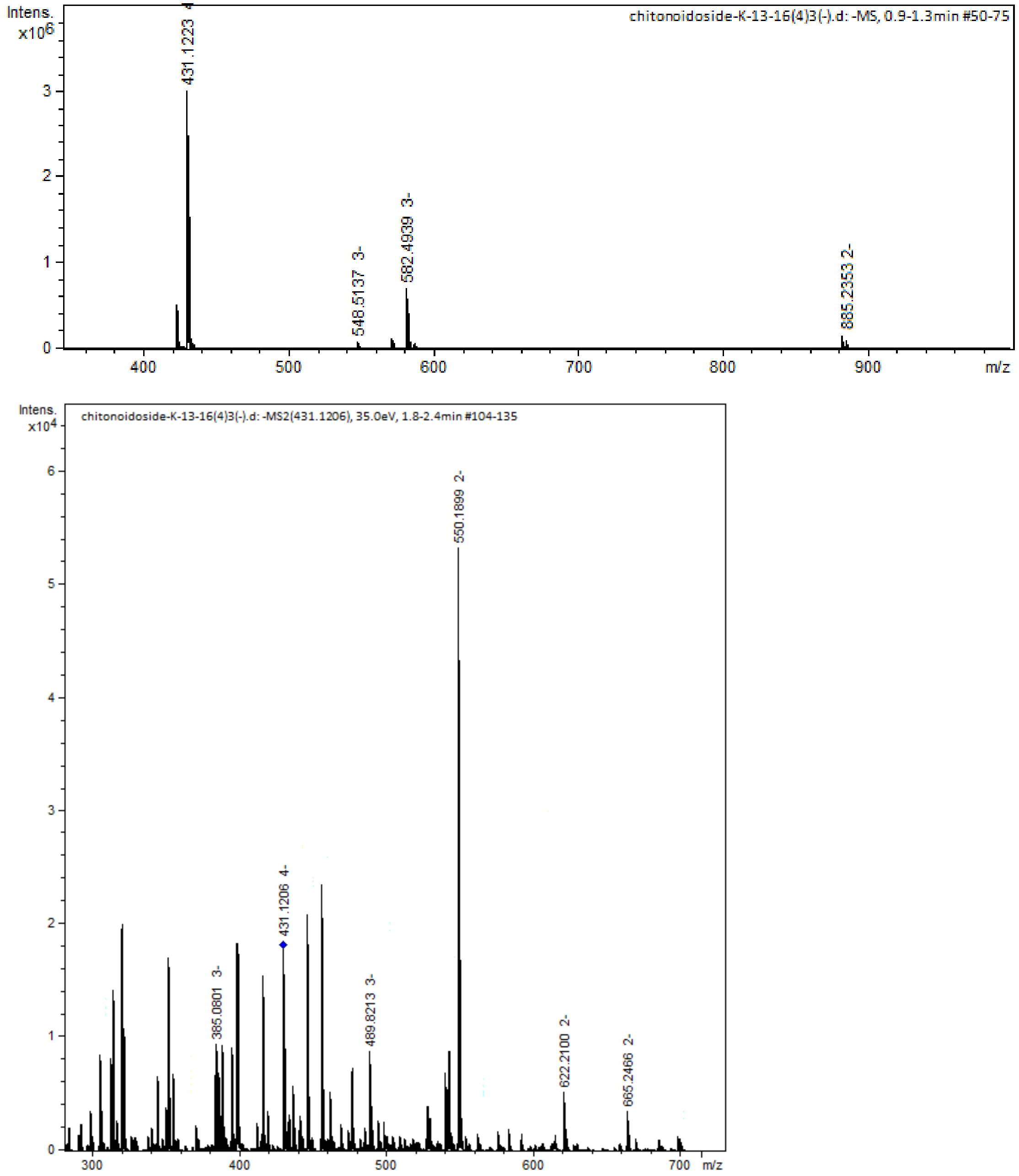

Figure S24. HR-ESI-MS and ESI-MS/MS spectra of chitonoidoside K (**3**)

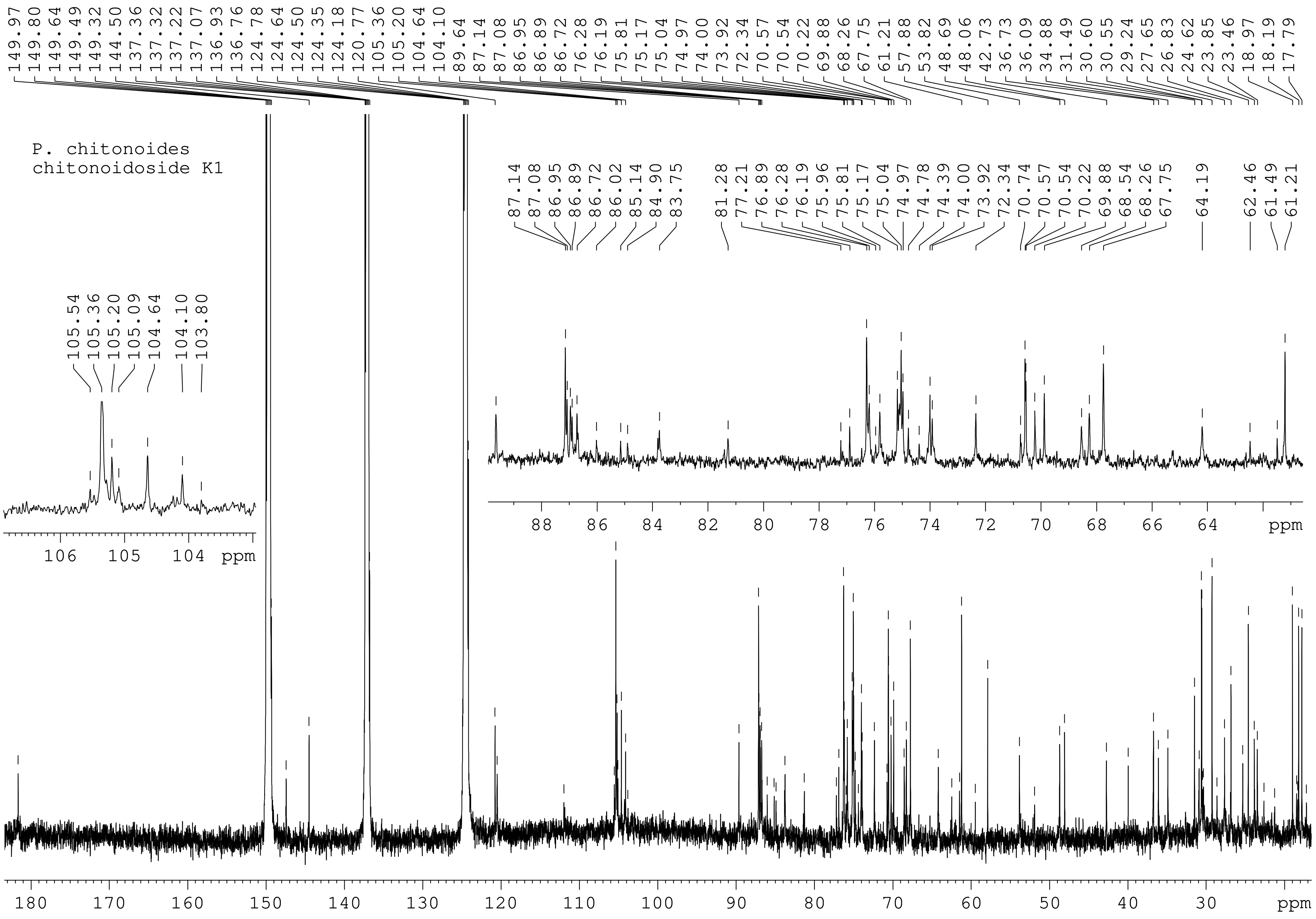

Figure S25. The ^{13}C NMR (125.67 MHz) spectrum of chitonoidoside K$_1$ (**4**) in C_5D_5N/D_2O (4/1)

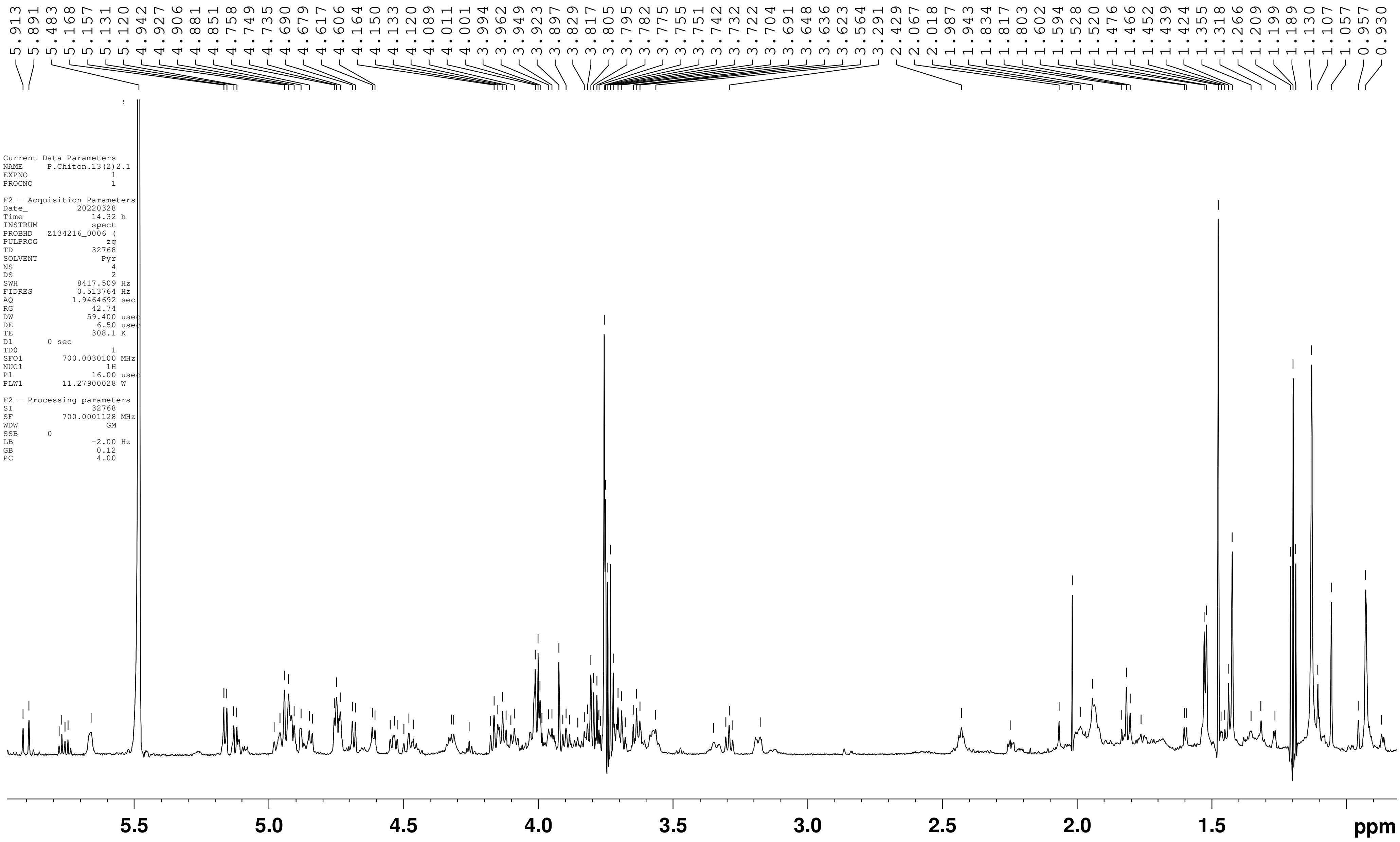

Figure S26. The ^{1}H NMR (500.12 MHz) spectrum of chitonoidoside K_1 (**4**) in C_5D_5N/D_2O (4/1)

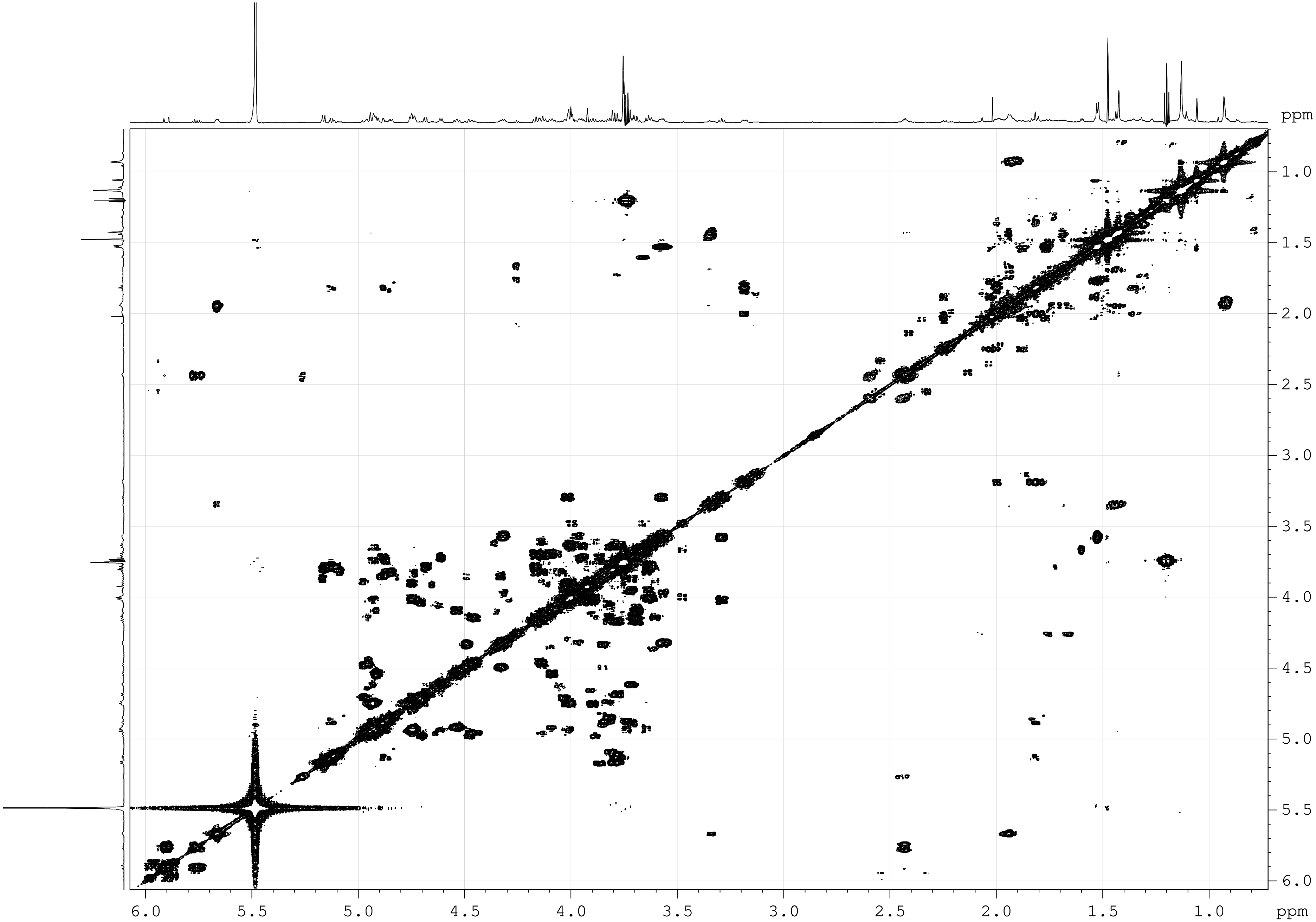

Figure S27. The COSY (500.12 MHz) spectrum of chitonoidoside K_1 (**4**) in C_5D_5N/D_2O (4/1)

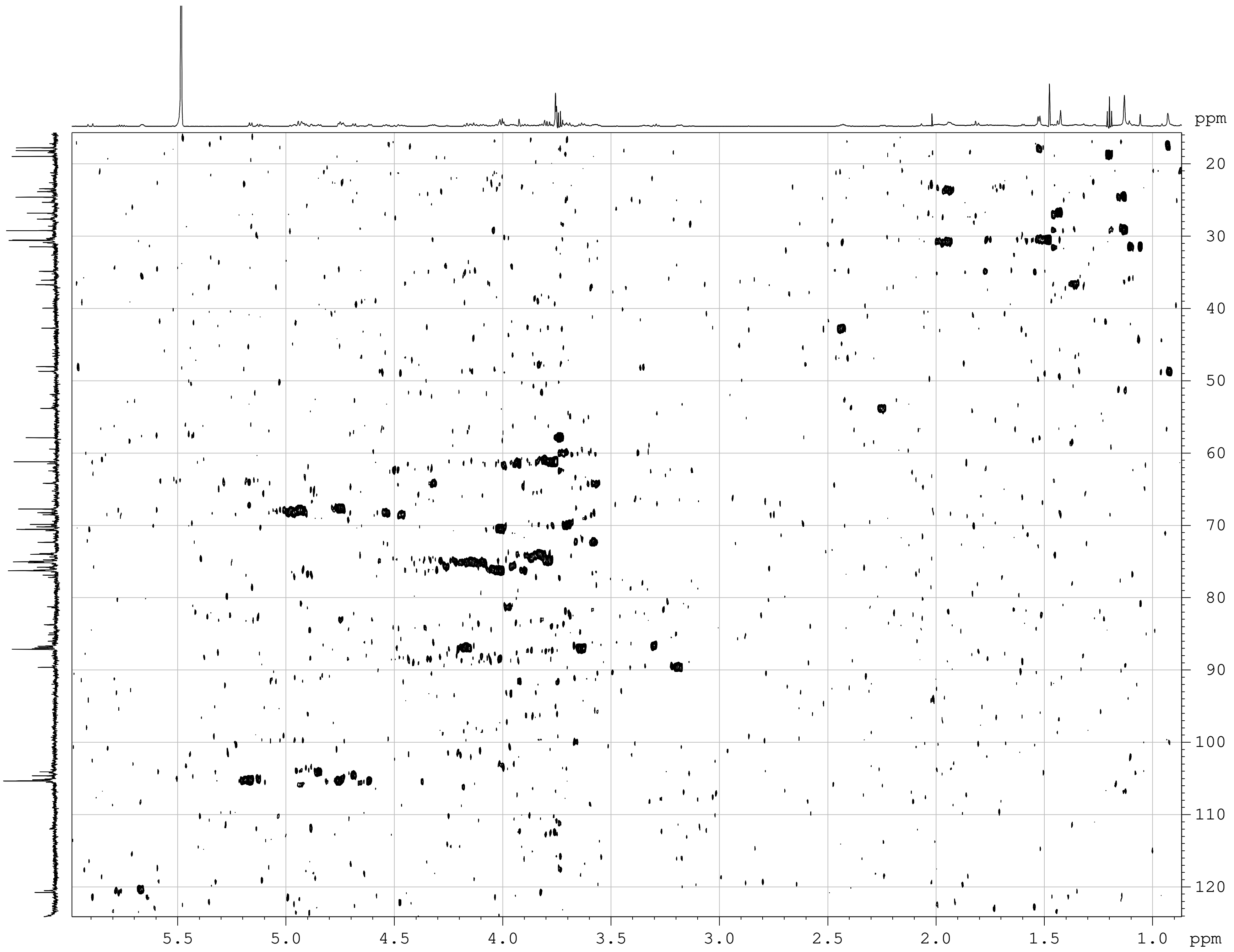

Figure S28. The HSQC (500.12 MHz) spectrum of chitonoidoside K$_1$ (**4**) in C_5D_5N/D_2O (4/1)

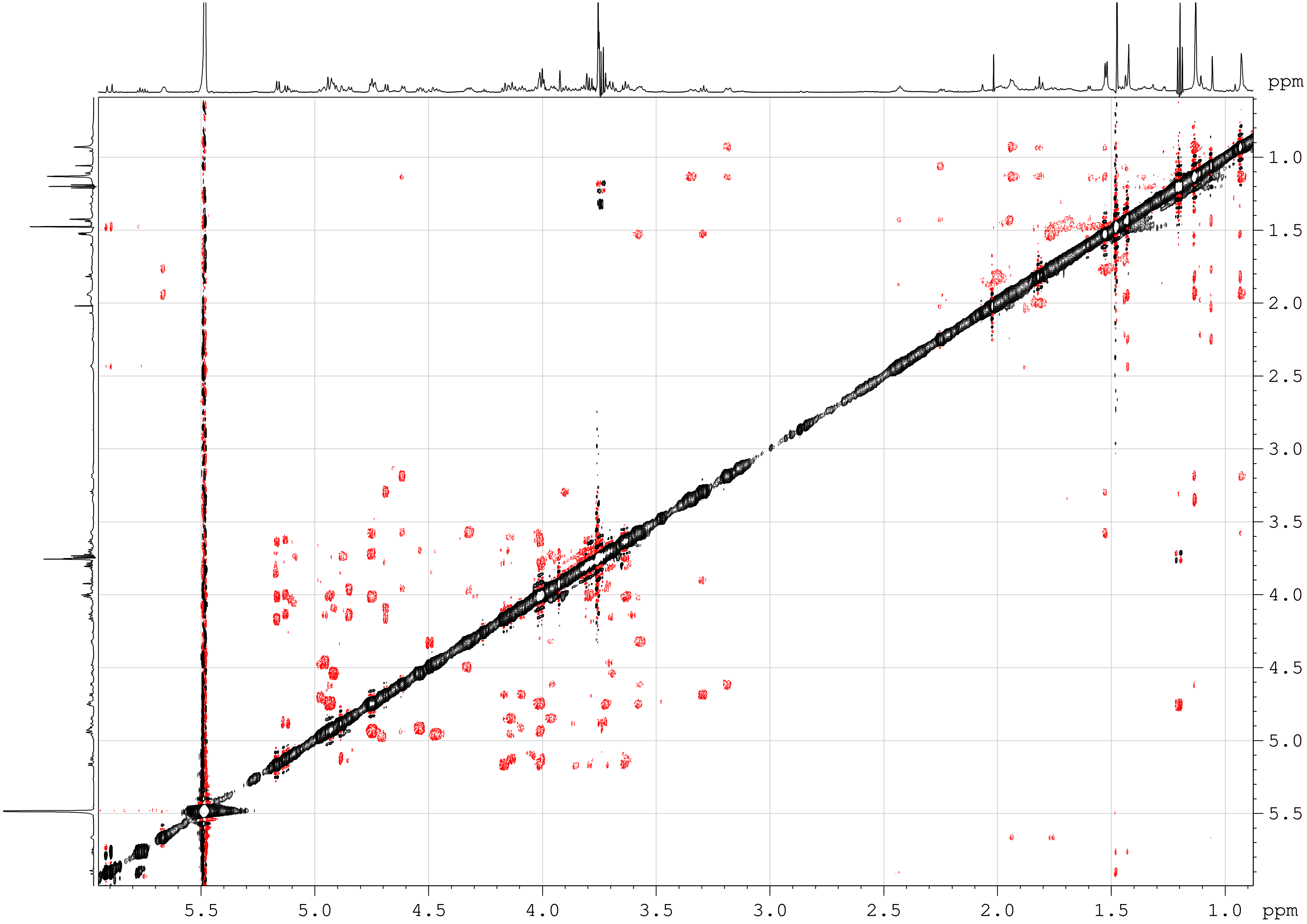

Figure S29. The ROESY (500.12 MHz) spectrum of chitonoidoside K1 (**4**) in C_5D_5N/D_2O (4/1)

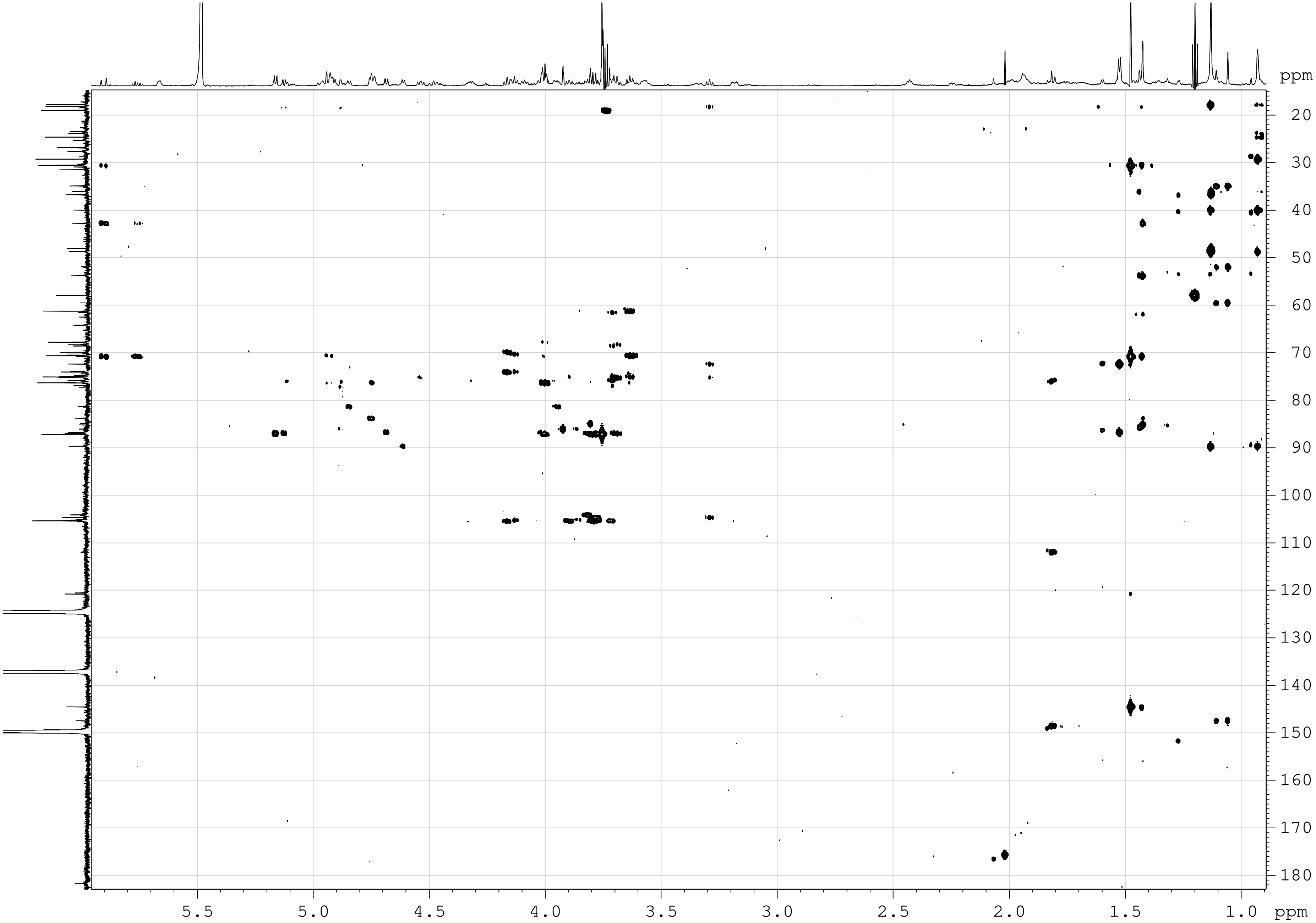

Figure S30. The HMBC (500.12 MHz) spectrum of chitonoidoside K_1 (**4**) in C_5D_5N/D_2O (4/1)

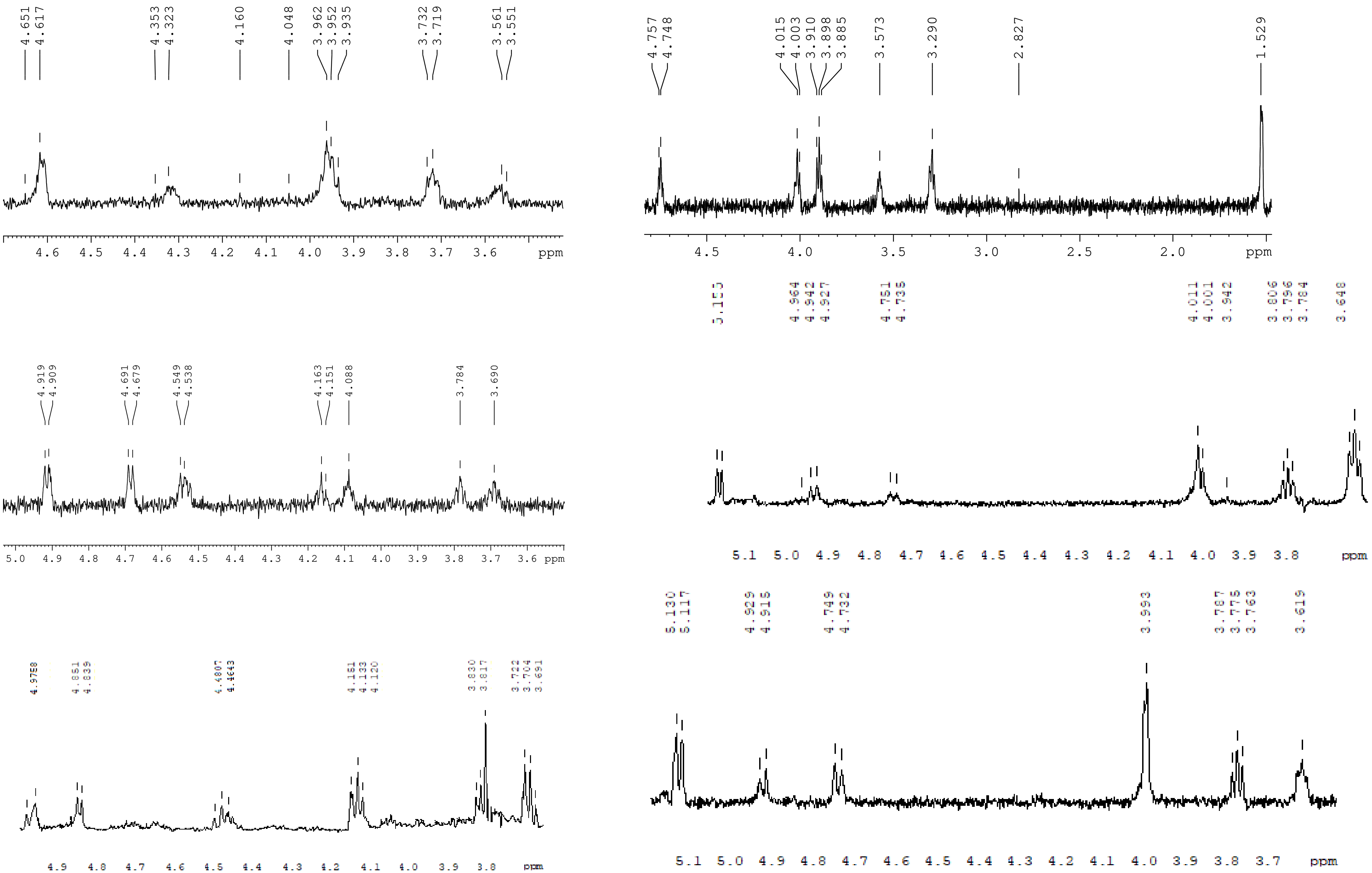

Figure S31. 1D TOCSY (500.12 MHz) spectra of Xyl1, Qui2, Glc3, MeGlc4, Glc5 and MeGlc6 of chitonoidoside K1 (**4**) in C_5D_5N/D_2O (4/1)

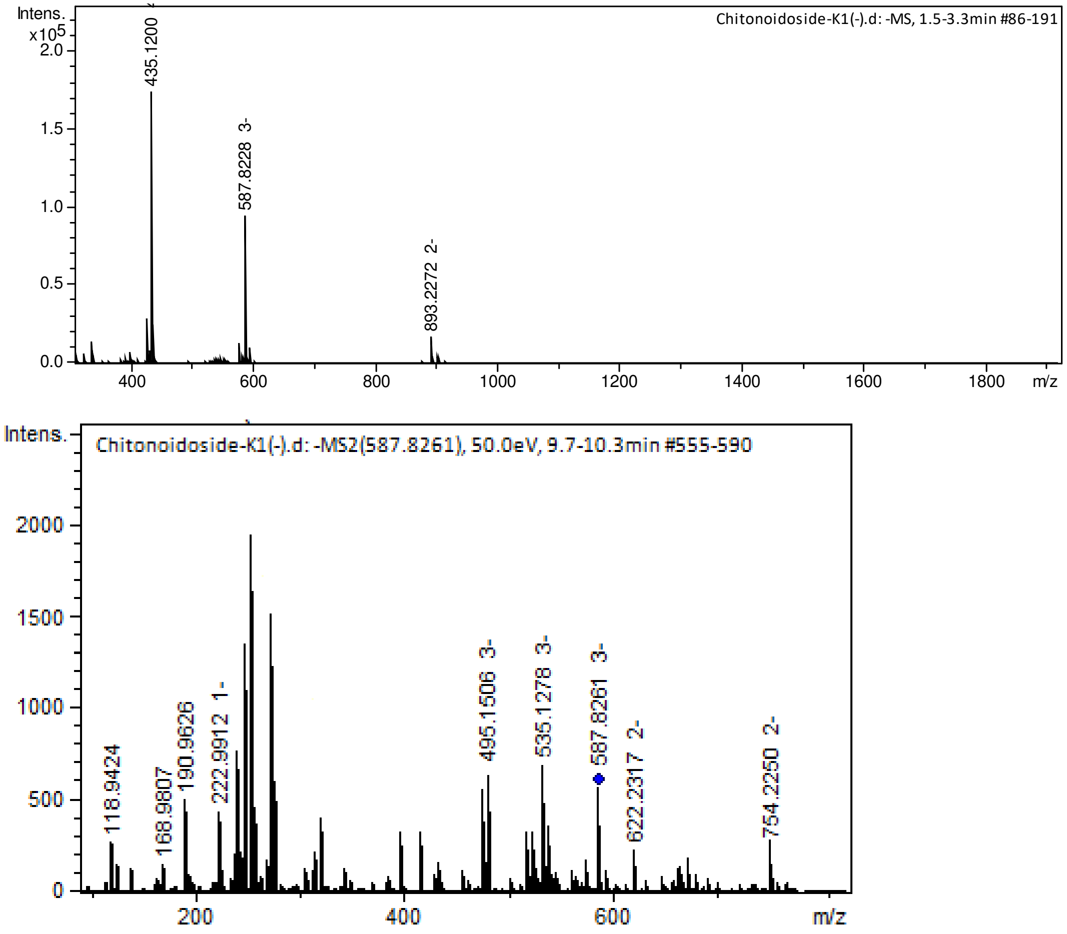

Figure S32. HR-ESI-MS and ESI-MS/MS spectra of chitonoidoside K_1 (**4**)

Table S3. ^{13}C and ^{1}H NMR chemical shifts, HMBC and ROESY correlations of the carbohydrate chain of chitonoidoside K_1 (**4**).

Atom	δ_C mult.[a]	δ_H mult. (*J* in Hz)[b,c,d]	HMBC	ROESY
Xyl1 (1→C-3)				
1	105.2 CH	4.61 d (7.0)	C: 3	H-3; H-3, 5 Xyl1
2	**83.7** CH	3.71 t (7.6)	C: 1 Qui2; 1 Xyl1	H-1 Qui2; H-4 Xyl1
3	75.8 CH	3.96 m	C: 4 Xyl1	H-1, 5 Xyl1
4	**81.3** CH	3.97 m		H-1 Glc5
5	64.2 CH_2	4.32 dd (4.1; 12.3)	C: 3 Xyl1	
		3.56 m		
Qui2 (1→2Xyl1)				
1	105.4 CH	4.75 d (7.0)	C: 2 Xyl1; 2 Qui2	H-2 Xyl1; H-3, 5 Qui2
2	76.2 CH	3.90 t (8.7)	C: 1 Qui2	H-4 Qui2
3	76.3 CH	4.01 t (8.7)	C: 2, 4 Qui2	H-5 Qui2
4	**86.7** CH	3.29 t (8.7)	C: 1 Glc3; 5 Qui2	H-1 Glc3; H-2 Qui2
5	72.3 CH	3.57 dd (6.1; 8.7)		H-1, 3 Qui2
6	18.5 CH_3	1.53 d (6.1)	C: 4, 5 Qui2	H-4, 5 Qui2
Glc3 (1→4Qui2)				
1	104.6 CH	4.69 d (8.5)	C: 4 Qui2	H-4 Qui2; H-3, 5 Glc3
2	74.8 CH	3.78 t (8.5)	C: 1, 3 Glc3	
3	**86.9** CH	4.16 t (8.5)	C: 1 MeGlc4; 2, 4 Glc3	H-1 MeGlc4; H-1 Glc3
4	69.9 CH	3.69 t (8.5)	C: 3, 5, 6 Glc3	H-6 Glc3
5	75.2 CH	4.09 m		H-1 Glc3
6	*68.3* CH_2	4.92 dd (11.3)		
		4.54 dd (5.7; 11.3)	C: 5 Glc3	H-4 Glc3
MeGlc4 (1→3Glc3)				
1	105.4 CH	5.16 d (7.1)	C: 3 Glc3	H-3 Glc3; H-3, 5 MeGlc4
2	75.0 CH	3.80 t (8.5)	C: 1, 3 MeGlc4	
3	87.1 CH	3.64 t (8.5)	C: 2, 4 MeGlc4, OMe	H-1, 5 MeGlc4
4	70.6 CH	4.00 m	C: 3, 5 MeGlc4	H-6 MeGlc4
5	76.3 CH	4.00 m		H-1, 3 MeGlc4
6	*67.7* CH_2	4.94 d (11.3)	C: 4, 5 MeGlc4	
		4.74 d (11.3)		
OMe	61.2 CH_3	3.76 s	C: 3 MeGlc4	
Glc5 (1→4Xyl1)				
1	104.1 CH	4.85 d (8.9)	C: 4 Xyl1	H-4 Xyl1; H-3, 5 Glc5
2	74.0 CH	3.83 m	C: 1, 3 Glc5	
3	**87.1** CH	4.13 t (8.9)	C: 1 MeGlc6; 2 Glc5	H-1 MeGlc6; H-1 Glc5
4	70.2 CH	3.71 m	C: 3, 5, 6 Glc5	H-6 Glc5
5	75.0 CH	4.13 m	C: 4 Glc5	
6	*68.5* CH_2	4.97 brd (11.8)		
		4.47 brd (11.8)		H-4 Glc5
MeGlc6 (1→3Glc5)				
1	105.2 CH	5.13 d (8.9)	C: 3 Glc5	H-3 Glc5; H-3, 5 MeGlc6
2	75.0 CH	3.77 t (8.9)	C: 1, 3 MeGlc6	
3	87.1 CH	3.62 t (8.9)	C: 2, 4 MeGlc6, OMe	H-1, 5 MeGlc6
4	70.6 CH	3.99 m	C: 3, 5 MeGlc6	H-2, 6 MeGlc6
5	76.3 CH	3.99 m		H-1 MeGlc6
6	*67.7* CH_2	4.92 d (10.8)	C: 4, 5 MeGlc6	
		4.74 d (10.8)		
OMe	61.4 CH_3	3.76 s	C: 3 MeGlc6	

[c] Bold = interglycosidic positions.

[d] Italic – sulfate positions.

[a]Recorded at 125.67 MHz in C_5D_5N/D_2O (4/1). [b]Recorded at 500.12 MHz in C_5D_5N/D_2O (4/1)

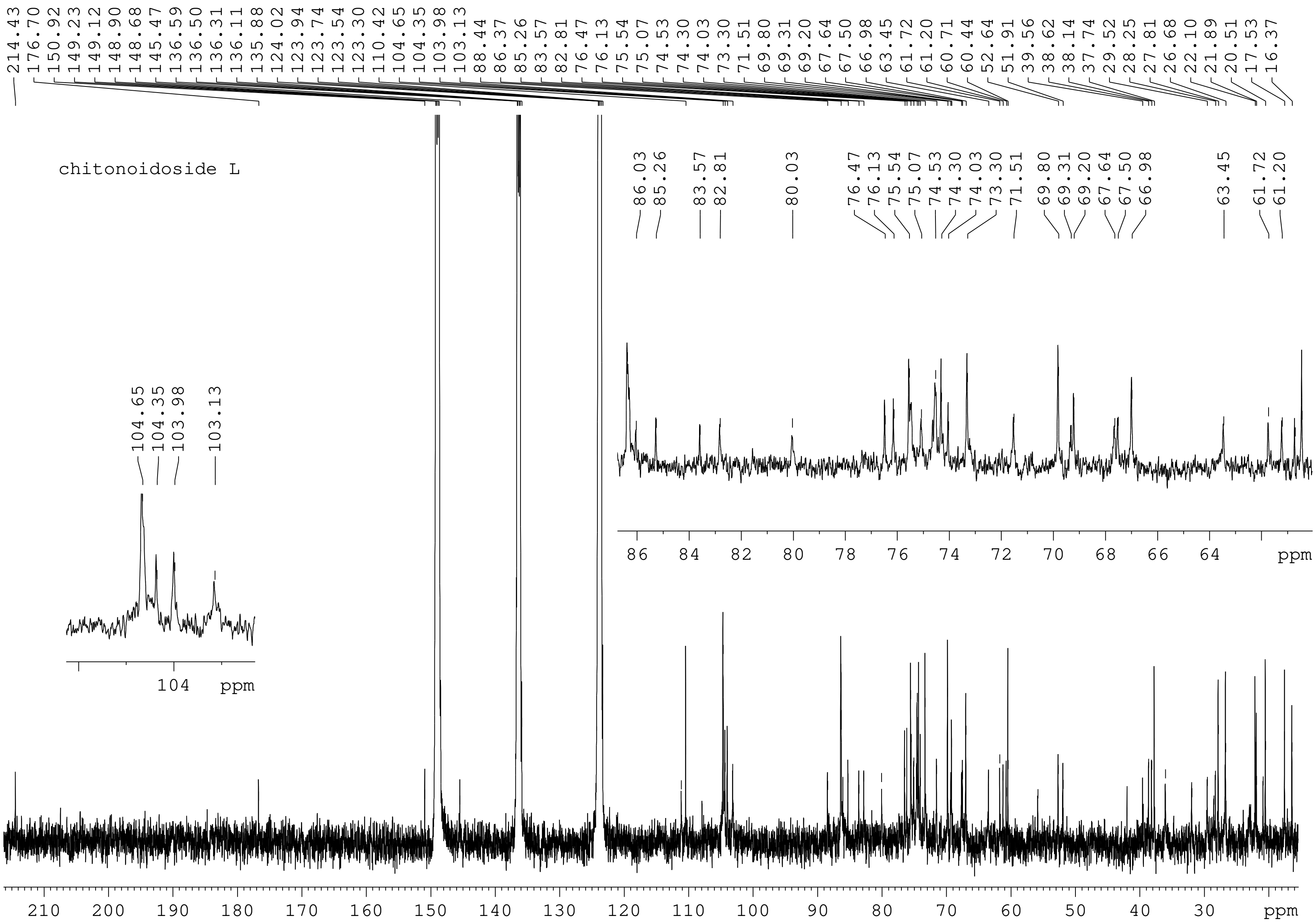

Figure S33. The ^{13}C NMR (125.67 MHz) spectrum of chitonoidoside L (**5**) in C_5D_5N/D_2O (4/1)

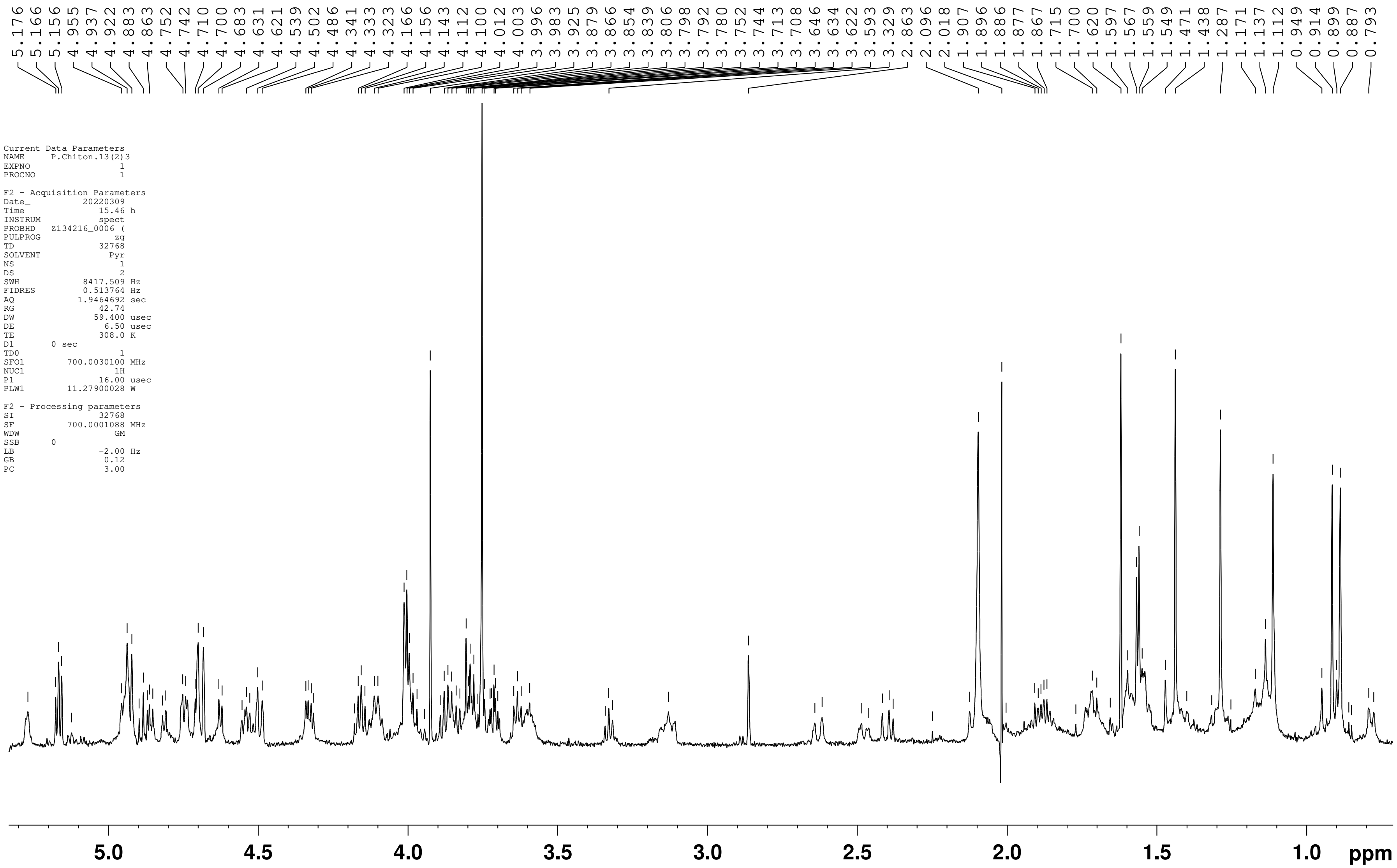

Figure S34. The ^{1}H NMR (500.12 MHz) spectrum of chitonoidoside L (**5**) in C_5D_5N/D_2O (4/1)

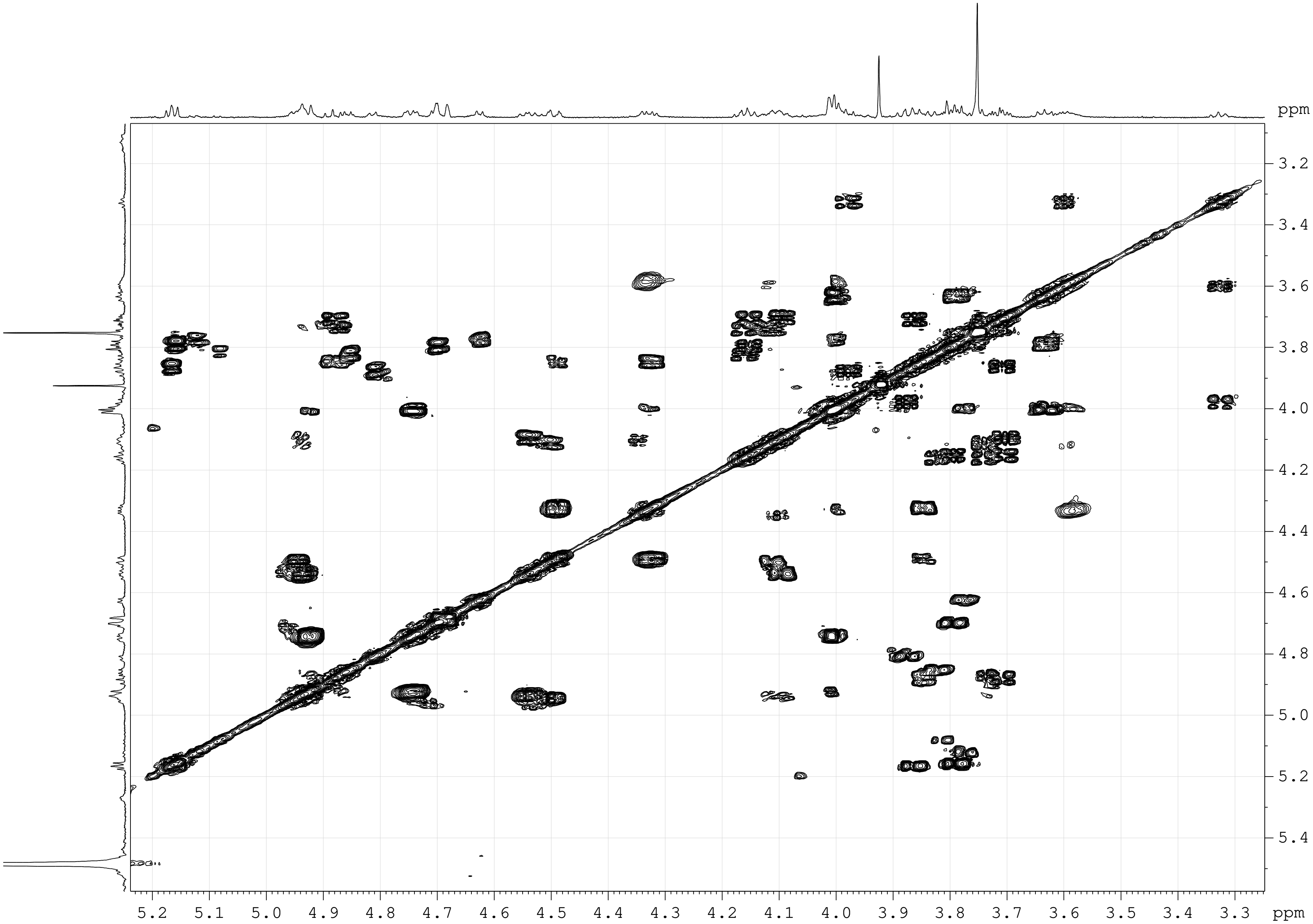

Figure S35. The COSY (500.12 MHz) spectrum of chitonoidoside L (**5**) in C_5D_5N/D_2O (4/1)

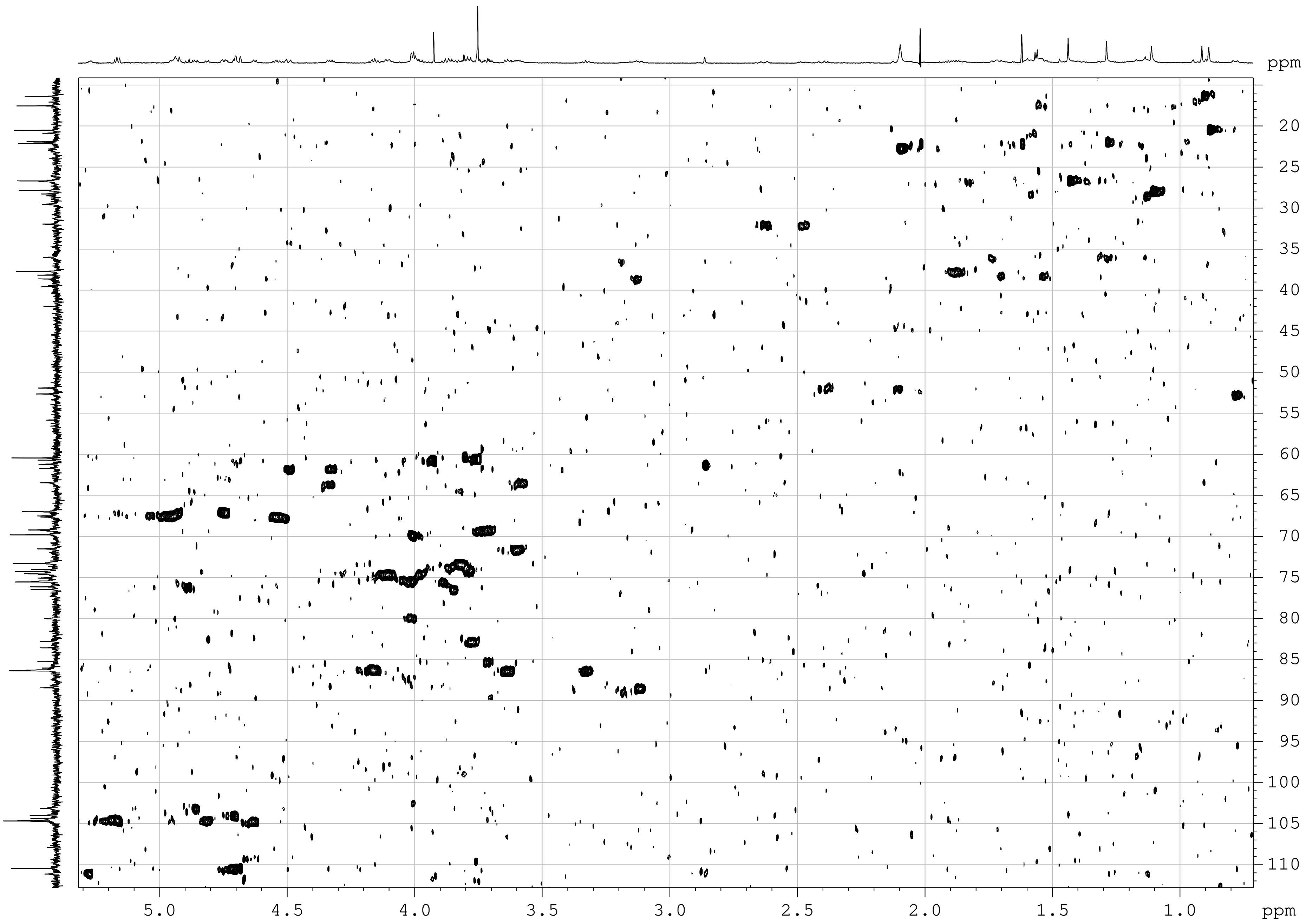

Figure S36. The HSQC (500.12 MHz) spectrum of chitonoidoside L (**5**) in C_5D_5N/D_2O (4/1)

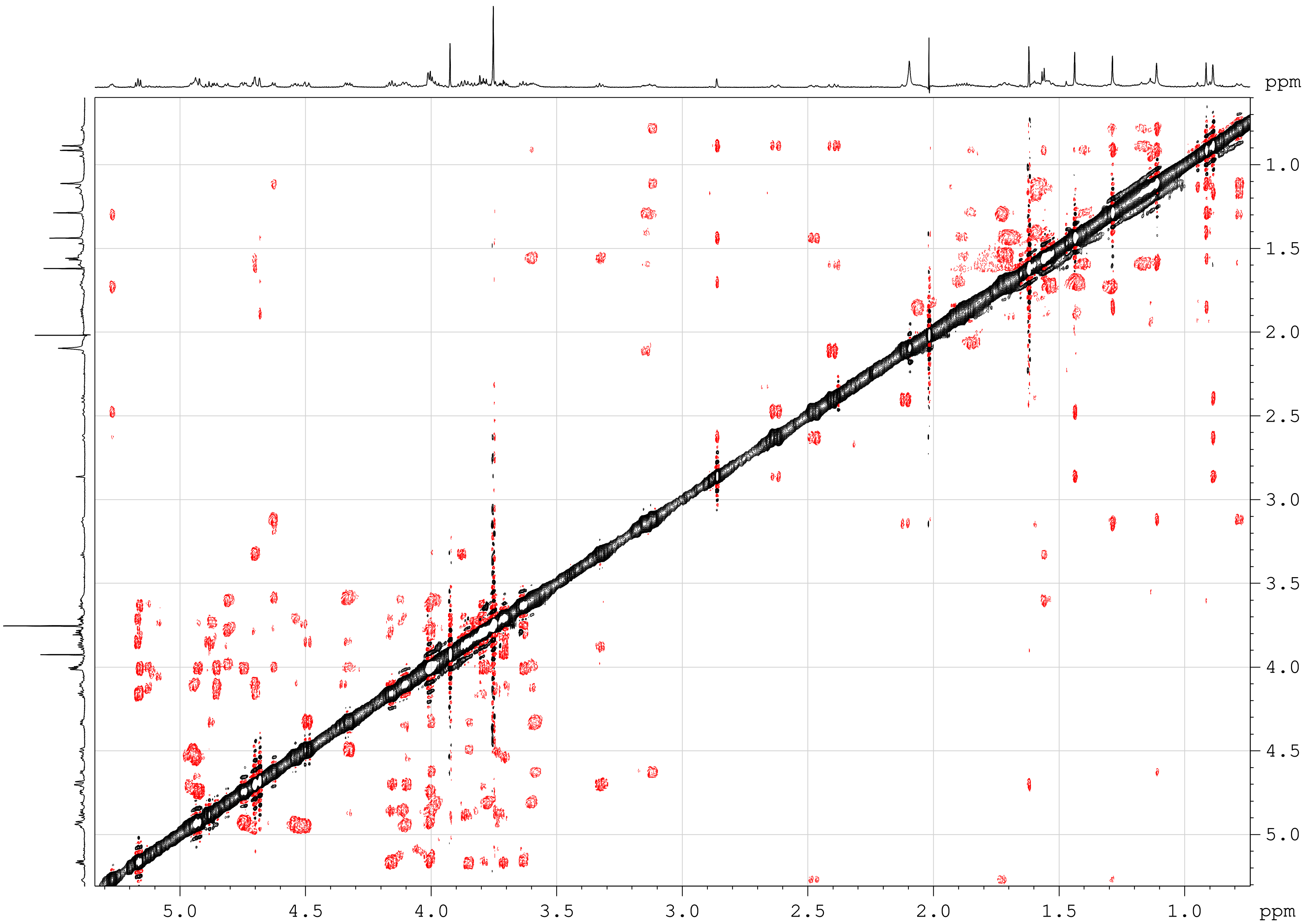

Figure S37. The ROESY (500.12 MHz) spectrum of chitonoidoside L (**5**) in C_5D_5N/D_2O (4/1)

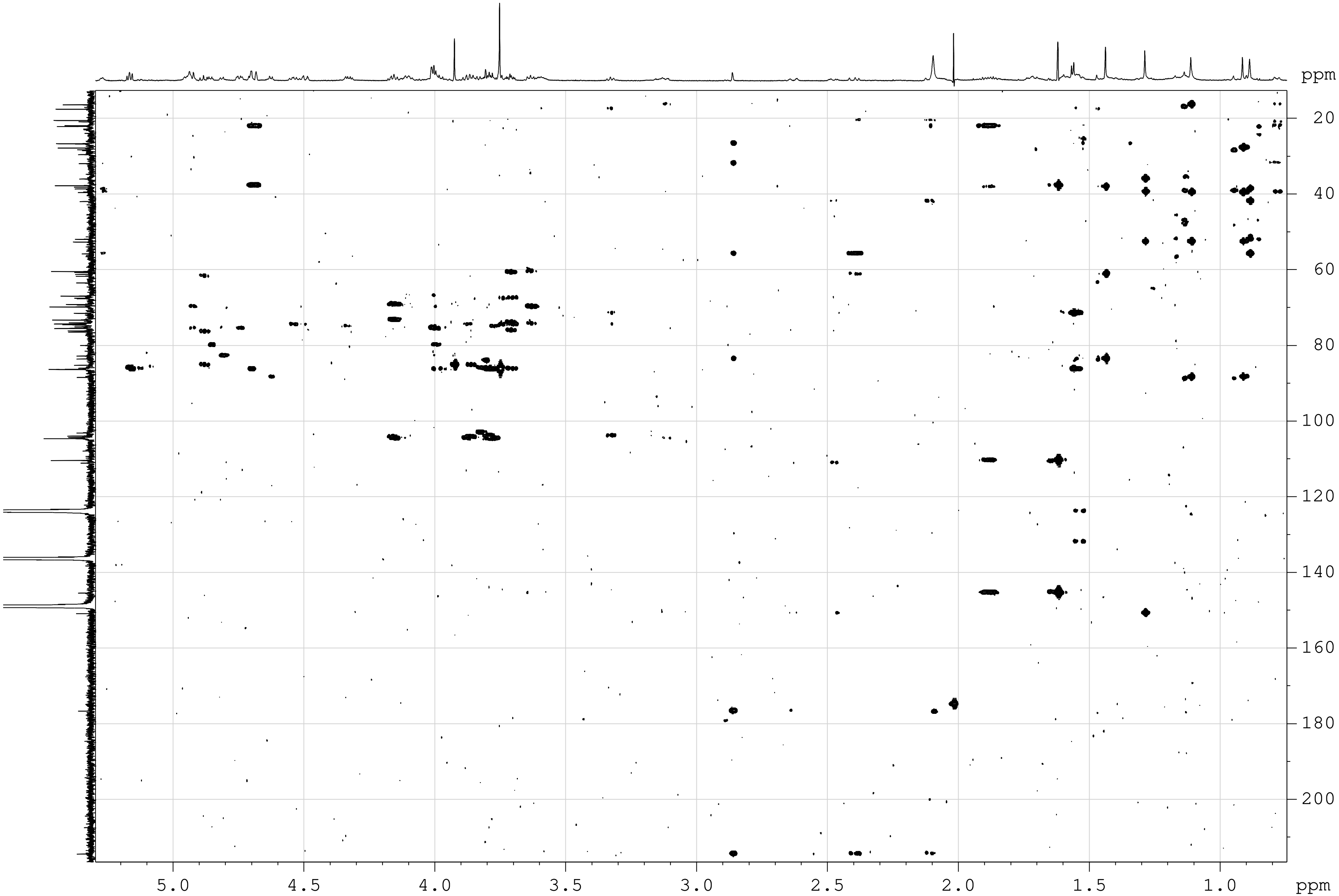

Figure S38. The HMBC (500.12 MHz) spectrum of chitonoidoside L (**5**) in C_5D_5N/D_2O (4/1)

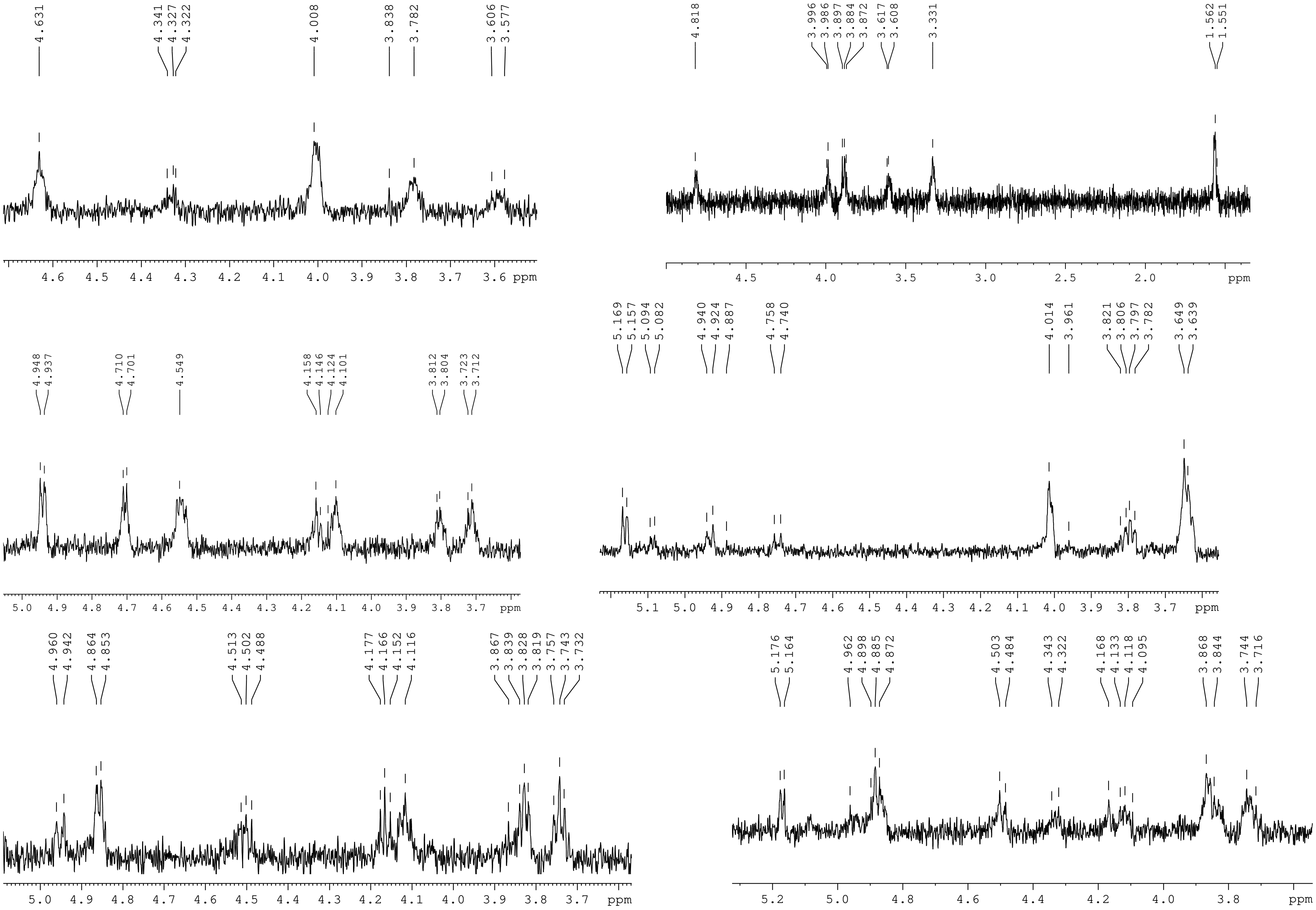

Figure S39. 1D TOCSY (500.12 MHz) spectra of Xyl1, Qui2, Glc3, MeGlc4, Glc5 and MeGlc6 of chitonoidoside L (**5**) in C_5D_5N/D_2O (4/1)

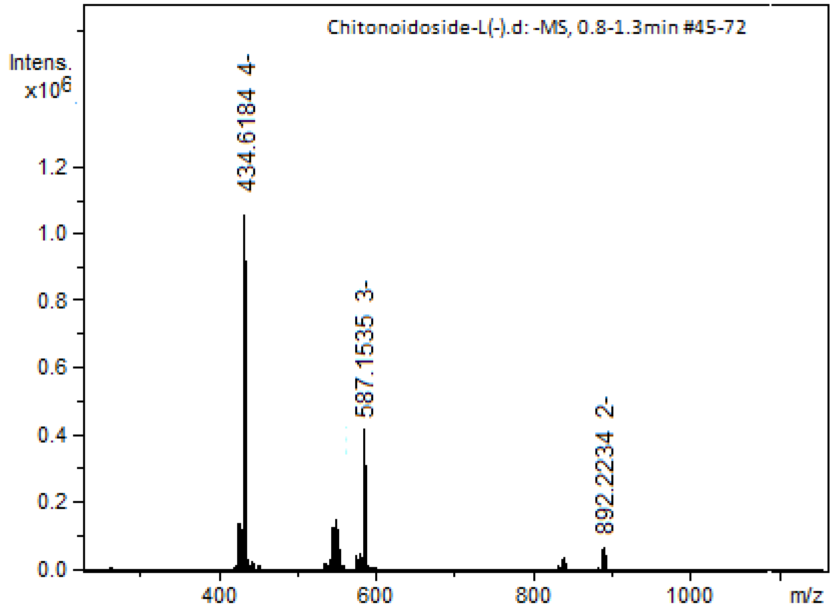

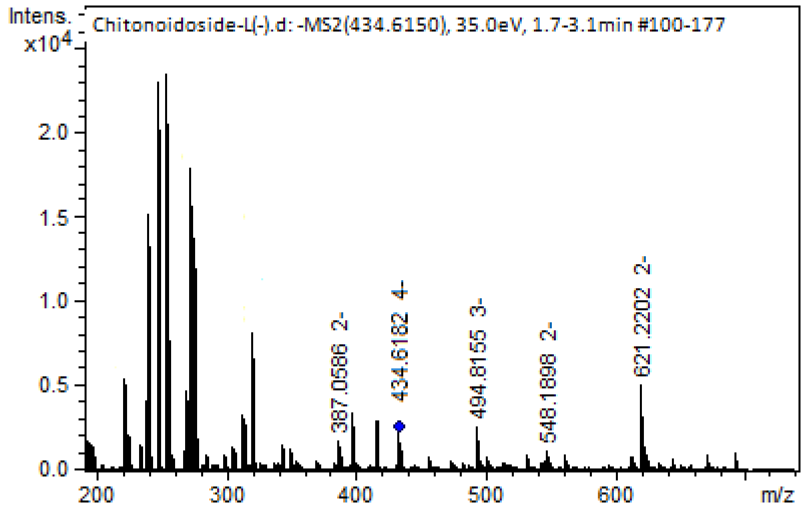

Figure S40. HR-ESI-MS and ESI-MS/MS spectra of chitonoidoside L (**5**)

Table S4. ^{13}C and ^{1}H NMR chemical shifts and HMBC and ROESY correlations of aglycone moiety of chitonoidoside L (**5**).

Position	δ_C mult.[a]	δ_H mult. (*J* in Hz)[b]	HMBC	ROESY
1	36.0 CH_2	1.74 m		H-119
		1.30 m		
2	26.7 CH_2	2.05 m		
		1.83 m		
3	88.4 CH	3.12 dd (3.6; 11.3)	C: 4, 30, 1 Xyl1	H-1, H-5, H-31, H1-Xyl1
4	39.6 C			
5	52.6 CH	0.78 brd (11.7)	C: 6, 10, 19, 30	H-1, H-3, H-31
6	20.9 CH_2	1.58 m		
		1.39 m		H-8, H-19
7	28.3 CH_2	1.58 m		
		1.13 m		H-5, H-32
8	38.6 CH	3.13 m		H-6, H-15, H-19
9	150.9 C			
10	39.5 C			
11	111.1 CH	5.27 m	C: 10, 13	H-1, H-19
12	31.9 CH_2	2.63 d (17.0)	C: 18	H-17, H-32
		2.48 dd (6.0; 17.0)	C: 11, 14	H-21
13	55.8 C			
14	42.0 C			
15	51.9 CH_2	2.41 d (15.5)	C: 13, 16, 17, 32	H-7, H-32
		2.11 d (15.5)	C: 14, 16, 18, 32	H-8
16	214.4 C			
17	61.2 CH	2.86 s	C: 12, 13, 16, 18, 20, 21	H-12, H-21, H-22, H-32
18	176.7 C			
19	21.9 CH_3	1.29 s	C: 1, 5, 9, 10	H-1, H-2, H-8, H-30
20	83.6 C			
21	26.7 CH_3	1.44 s	C: 17, 20, 22	H-12, H-17, H-22
22	38.1 CH_2	1.70 m		H-21
		1.54 m		
23	22.0 CH_2	1.66 m		
		1.43 m		
24	37.7 CH_2	1.87 m	C: 22, 23, 26	
25	145.5 C			
26	110.4 CH_2	4.70 brs	C: 24, 27	H-21, H-22, H-24, H-27
		4.68 brs	C: 24, 27	H-21, H-22, H-24, H-27
27	22.1 CH_3	1.62 s	C: 24, 26	
30	16.4 CH_3	0.92 s	C: 3, 4, 5, 31	H-2, H-6, H-31,
31	27.8 CH_3	1.11 s	C: 3, 4, 5, 30	H-3, H-5, H-6, H-30
32	20.5 CH_3	0.89 s	C: 8, 13, 14, 15	H-7, H-12, H-15, H-17

[a] Recorded at 125.67 MHz in C_5D_5N. [b] Recorded at 500.12 MHz in C_5D_5N.
